# FAM69C functions as a kinase for eIF2α and promotes stress granule assembly

**DOI:** 10.15252/embr.202255641

**Published:** 2023-03-16

**Authors:** Zhongyan Wu, Fan Mei, Yangyang Gan, Anhang Liu, Jiapan Hu, Yan Jin, Yuxin Yin

**Affiliations:** ^1^ Institute of Systems Biomedicine, Department of Pathology, School of Basic Medical Sciences Peking University Health Science Center Beijing China; ^2^ Institute of Precision Medicine Peking University Shenzhen Hospital Shenzhen China

**Keywords:** eIF2α, FAM69C, inflammasome, microglia, stress granule, Immunology, Neuroscience, Signal Transduction

## Abstract

Stress granules are dynamic cytoplasmic ribonucleoprotein granules that assemble in response to cellular stress. Aberrant formation of stress granules has been linked to neurodegenerative diseases. However, the molecular mechanisms underlying the initiation of stress granules remain elusive. Here we report that the brain‐enriched protein kinase FAM69C promotes stress granule assembly through phosphorylation of eukaryotic translation initiation factor 2 (eIF2α). FAM69C physically interacts with eIF2α and functions as a stress‐specific kinase for eIF2α, leading to stress‐induced protein translation arrest and stress granule assembly. Primary microglia derived from *Fam69c* knockout mice exhibit aberrant stress granule assembly in response to oxidative stress and ATP. Defective stress granule assembly in microglia correlates with the formation of ASC specks and NLRP3 inflammasome activation, whereas induction of stress granule precludes inflammasome formation. Consistently, increased NLRP3 levels, caspase‐1 cleavage and *Il18* expression corroborate microglia‐associated neuroinflammation in aged *Fam69c* knockout mice. Our study demonstrates that FAM69C is critical for stress granule assembly and suggests its role in the regulation of microglia function.

## Graphical abstract

FAM69C is a novel stress‐specific kinase for eIF2α and promotes stress granule assembly, which precludes NLRP3 inflammasome activation in microglia.

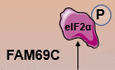

## Introduction

Translational control in eukaryotic cells is critical for cell survival during stress responses (Sonenberg & Hinnebusch, 2009). Regulation of protein translation is imposed at the initiation stage. The phosphorylation of the α‐subunit of eukaryotic translation initiation factor 2 (eIF2α) decides the integrity of the preinitiation complex and translation initiation (Ivanov *et al*, 2019). There are four known kinases that are responsible for the phosphorylation of eIF2α under specific conditions, including PKR‐like ER kinase (PERK), double‐stranded RNA‐dependent protein kinase (PKR), heme‐regulated eIF2α kinase (HRI), and general control non‐derepressible protein 2 (GCN2) (Pakos‐Zebrucka *et al*, 2016).

Stress granules (SGs) form when translation initiation is inhibited during a variety of stress responses, including heat shock (HS), nutrient deprivation, hypo‐osmotic conditions, and oxidative stress (Protter & Parker, 2016). SGs have been proposed to stabilize mRNAs, regulate mRNA translation and have impact on physiological and pathological processes (Buchan & Parker, 2009). SG assembly entraps viral mRNAs and inhibits viral gene expression in antiviral immune response (McCormick & Khaperskyy, 2017). Phosphorylation of eIF2α and SG assembly promote the survival of cells under stress (Aulas *et al*, 2017). In tumor cells, SG assembly inhibits mTORC1 and apoptosis through SG component astrin (Thedieck *et al*, 2013). SG assembly protects neuron and other long‐lived cells from death under stress (Farley & Watkins, 2018). Mutation of *FUS* (Baron *et al*, 2013; Bosco *et al*, 2010) and expanded *C9ORF72* hexanucleotide repeat (Boeynaems *et al*, 2017) in neurodegenerative diseases are associated with defects in the disassembly of SGs, resulting in the phosphorylation of cytosolic TDP43 and inhibition of nuclear import (Gasset‐Rosa *et al*, 2019; Wolozin & Ivanov, 2019).

Neurodegeneration is characterized by progressive loss of neuron activity and function, ultimately leading to neuron death. It is well known that pathologic protein aggregation triggers neurodegeneration in neurodegenerative disease. Amyloidosis, tauopathies, a‐synucleinopathies, and TDP‐43 proteinopathies represent the predominant type of pathological hallmarks (Dugger & Dickson, 2017; Vaquer‐Alicea & Diamond, 2019). Pathologic protein aggregates have been considered to be the cause of neurodegeneration, while the immune changes in neurodegenerative diseases were considered as a secondary or reactive response to protein aggregates and neurodegeneration. With the development of sequencing technology, the microglial‐specific gene *TREM2* R47H mutation identified with genome‐wide association studies (GWASs) suggest that dysregulated immune responses contribute to the disease etiology (Jonsson *et al*, 2013).

Microglia are the main resident innate immune cells in the central nervous system (CNS). Like macrophages, microglia activation can be categorized as either classical (M1) or alternative (M2), representing the proinflammatory and anti‐inflammatory states of microglia (Colonna & Butovsky, 2017). Microglia not only recognize pathogen associated molecular patterns (PAMPs) but also can be activated by danger‐associated molecular patterns (DAMPs) or protein aggregates (Labzin *et al*, 2018). Extracellular misfolded and aggregated proteins can activate microglia through pattern recognition receptors. Activation of Toll‐like receptors (TLRs) triggers NF‐κB‐dependent proinflammatory gene expression and the induction of proinflammatory cytokines (Lehnardt, 2010). Moreover, microglia express various purinergic receptors for ATP, such as P2Y12 and P2X4, to detect cell injuries (Colonna & Butovsky, 2017). NLRP3 inflammasome mediates M1 macrophage polarization (Zhang *et al*, 2020) and has been found in various neurodegenerative diseases including Alzheimer's disease (Heneka *et al*, 2015), frontotemporal dementia (McCauley & Baloh, 2019), and Parkinson's disease (Marogianni *et al*, 2020). NLRP3 inflammasomes induce the cleavage of caspase‐1, which is responsible for the maturation and secretion of the inflammatory cytokines Interleukin‐1β (IL‐1β) and IL‐18, and additional pyroptosis (Kanneganti *et al*, 2006; Voet *et al*, 2019). Moreover, previous studies implicated that NLRP3 inflammasomes are essential for the development and progression of amyloid‐β (Aβ) pathology and tau pathology in mouse models (Venegas *et al*, 2017; Ising *et al*, 2019).

As the predominant types of stress responses, crosstalk between stress granule and NLRP3 inflammasome has been studied. On one hand, inflammation upregulated eIF2α phosphorylation and stress granule formation in heat‐shocked intestinal epithelial cells (Hu *et al*, 2010), whereas anti‐inflammatory cytokine treatment reversed eIF2α phosphorylation and SGs formation (Herman *et al*, 2019). On the other hand, SG assembly inhibits the formation of NLRP3 inflammasomes through the sequestration of HSP90 and DDX3X in SGs (Mayor *et al*, 2007; Samir *et al*, 2019). Additionally, SG assembly directly affects phagocytosis of microglia through the sequestration of SYK (Ghosh & Geahlen, 2015).

In our previous studies, we demonstrated that FAM69C is a brain‐enriched kinase that regulates synaptic plasticity and memory and is associated with neurodegenerative dementia (Mei *et al*, 2022). In this study, we explore the function of FAM69C as an eIF2α kinase that promotes stress granule assembly and suggest its role in the regulation of microglia function.

## Results

### FAM69C is a stress‐specific kinase for eIF2α

**Figure 1:**
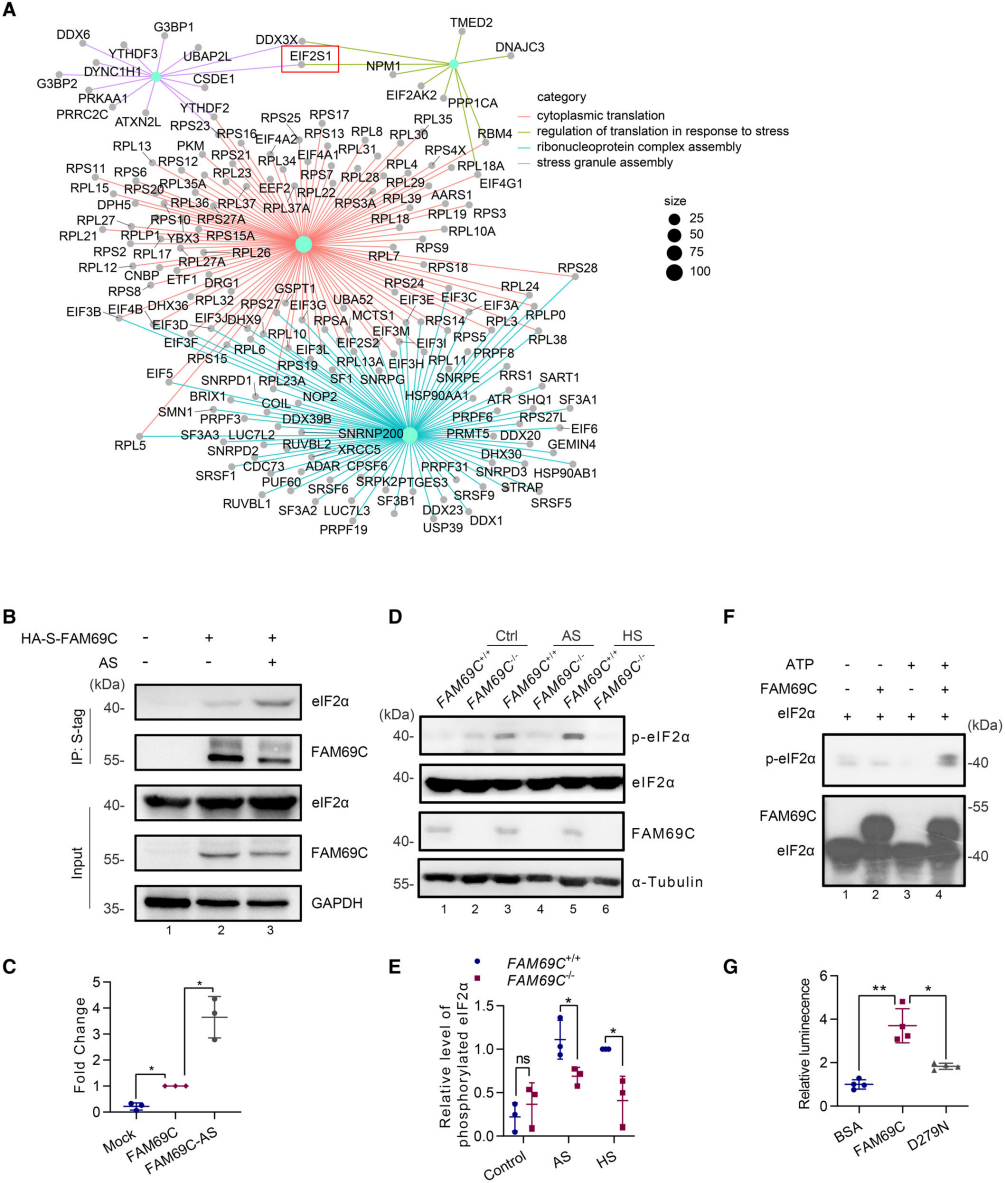
FAM69C is a stress‐specific kinase for eIF2α A
Cnetplot of enriched pathways for ‘cytoplasmic translation’, ‘regulation of translation in response to stress’, ‘ribonucleoprotein complex assembly’ and ‘stress granule assembly’. S‐tag pull‐down of FAM69C in SH‐SY5Y cells and consequent mass spectrometry identified a series of potential proteins interacting with FAM69C.B
Immunoblot analysis of immunoprecipitates with endogenous eIF2α and HA‐S‐tagged FAM69C. SH‐SY5Y cells were transfected to express HA‐S‐FAM69C, followed by 0.5 mM AS treatment for 30 min. HA‐S‐FAM69C was pulled‐downed with S‐protein beads, and the S‐protein immunoprecipitants were immunoblotted for eIF2α and HA. Representative blots, *n* = 3 biologically independent experiments.C
Quantitative analysis of eIF2α in the FAM69C Pull‐down assay. *P value < 0.05, paired two‐tailed *t*‐test, *n* = 3 biologically independent experiments. Mean ± SD. For FAM69C versus mock, *P* value = 0.0103; For FAM69C versus FAM69C‐AS, *P* value = 0.0289.D
Immunoblot analysis of phosphorylated eIF2α in *FAM69C* ^+/+^ and *FAM69C* ^−/−^ SH‐SY5Y cells. Cells were treated with 0.5 mM AS, 42°C heat shock for 30 min, and lysates were immunoblotted with p‐eIF2α (S51) antibody. Representative blots, *n* = 3 biologically independent experiments.E
Quantitative analysis of the phosphorylation level of eIF2α normalized with total eIF2α. *P value < 0.05, paired two‐tailed *t*‐test, *n* = 3 biologically independent experiments. Mean ± SD. For AS, *P* value = 0.0196; For HS, *P* value = 0.0335.F
Immunoblot analysis of phosphorylated eIF2α in the *in vitro* phosphorylation assay. 10 μg recombinant eIF2α protein was mixed with or without 1 μg FAM69C protein in reaction buffer (40 mM Tris–HCl (pH7.5), 20 mM MgCl_2_, 1 mM ATP) at 37°C for 30 min. Phosphorylated eIF2α was detected with the p‐eIF2α (S51) antibody. Representative blots, *n* = 3 biologically independent experiments.G
The relative luminescence in ADP‐Glo kinase assay. ADP Generated by FAM69C and the kinase‐dead mutant D279N in the *in vitro* phosphorylation assay was quantified with ADP‐Glo kinase assay, BSA was used as a control. **P value = 0.0084, *P value = 0.0239, paired two‐tailed *t*‐test, *n* = 4 biologically independent experiments. Mean ± SD.

In our previous work, we characterized a brain‐enriched kinase FAM69C and demonstrated that FAM69C deficiency is associated with neurodegenerative dementia. To unveil the biological function of FAM69C, we performed S‐tag pulldown assay to identify its interactive proteins. Mass spectrometry analyses identified a total of 1,355 potential candidates associated with FAM69C protein (as assessed by > 5‐fold intensity as compared to HA‐S‐tag control, Dataset EV1). Biological pathway analyses indicated that these candidates were enriched in pathways relative to stress granule (SG) assembly, regulation of translation in response to stress, ribonucleoprotein complex assembly, and cytoplasmic translation (Fig 1A). These results indicate that FAM69C is potentially involved in stress responses. Notably, *EIF2S1*‐encoded eIF2α protein is a key component of the preinitiation complex that initiates protein translation (Wolozin & Ivanov, 2019), and the reversible phosphorylation of eIF2α mediates stress‐induced protein synthesis arrest and SG assembly (McCormick & Khaperskyy, 2017). Thus, we speculated that eIF2α was a critical molecule that coupled regulation of translation in response to stress and SG assembly, the two FAM69C‐associated biological pathways. Next, we examined the physical interaction of FAM69C and eIF2α. Under the basal condition, immunoprecipitation in SH‐SY5Y cells indicated that the endogenous eIF2α interacted with FAM69C. Further, we treated SH‐SY5Y cells with SG inducer sodium arsenite (AS) and observed that the interaction of FAM69C and eIF2α was significantly strengthened (Fig 1B and C).

**Figure EV1:**
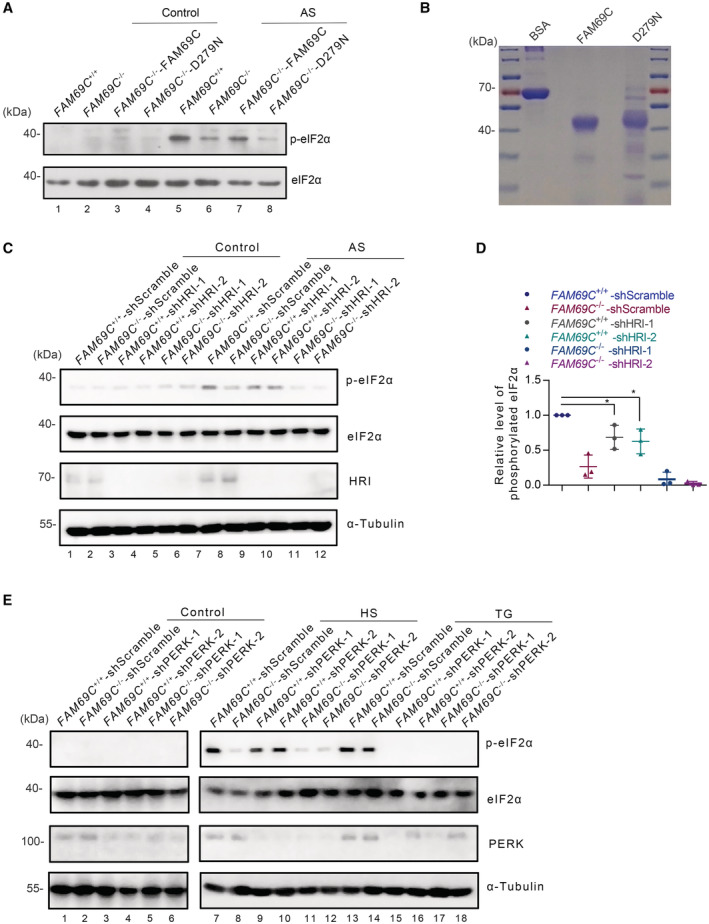
FAM69C is a stress‐specific kinase for eIF2α A
Immunoblot analysis of phosphorylated eIF2α in SH‐SY5Y cells. FAM69C^−/−^ SH‐SY5Y cells were transfected to express wildtype FAM69C and FAM69C D279N. Cells were treated with 0.5 mM AS for 30 min, and lysates were immunoblotted with the p‐eIF2α (S51) antibody.B
Coomassie Blue Staining showed the protein level of BSA, FAM69C and D279N proteins used in the ADP‐Glo kinase assay.C
Immunoblot analysis of phosphorylated eIF2α in HRI‐knockdown SH‐SY5Y cells. Cells were treated with or without 0.5 mM AS for 30 min, and lysates were immunoblotted with p‐eIF2α (S51), eIF2α, HRI, and α‐Tubulin antibody.D
Quantitative analysis of the phosphorylation level of eIF2α normalized with total eIF2α under AS treatment. *P value < 0.05, paired *t*‐test, *n* = 3 biologically independent experiments. Mean ± SD. For *FAM69C* ^−/−^ shHRI‐1, *P* value = 0.0431; For *FAM69C* ^−/−^ shHRI‐2, *P* value = 0.0337.E
Immunoblot analysis of phosphorylated eIF2α in PERK‐knockdown SH‐SY5Y cells. Cells were treated with 42°C heat shock (30 min), 4 μM TG (6 h). The cell lysates were immunoblotted with p‐eIF2α (S51), eIF2α, PERK, and α‐Tubulin antibody.

Based on the evident interaction between eIF2α and FAM69C, we next determined whether eIF2α might be a substrate of FAM69C. We treated *FAM69C* ^+/+^ and *FAM69C* ^−/−^ SH‐SY5Y cells with AS or heat shock (HS), which were the two conditions that strictly depend on eIF2α for SG induction. Notably, western blotting revealed that levels of phosphorylated eIF2α at S51 in *FAM69C* ^+/+^ cells were significantly increased upon AS or HS treatment, whereas loss of *FAM69C* deteriorated eIF2α phosphorylation (Fig 1D and E). By contrast, the D279N mutant, in which the kinase activity was impaired by substituting the critical amino acid Asp 279 in the catalytic loop with Asn, reduced eIF2α phosphorylation (Fig EV1A). Further, we determined whether FAM69C directly phosphorylates eIF2α using recombinant FAM69C^44–419^ and full‐length eIF2α proteins. In the *in vitro* phosphorylation assay, FAM69C protein directly phosphorylated eIF2α, as indicated by western blotting (Fig 1F). Consistently, the ADP‐Glo kinase assay corroborated that FAM69C phosphorylates eIF2α through its kinase activity (Figs 1G and EV1B). These data indicated that FAM69C functions as a kinase for eIF2α.

**Figure 2:**
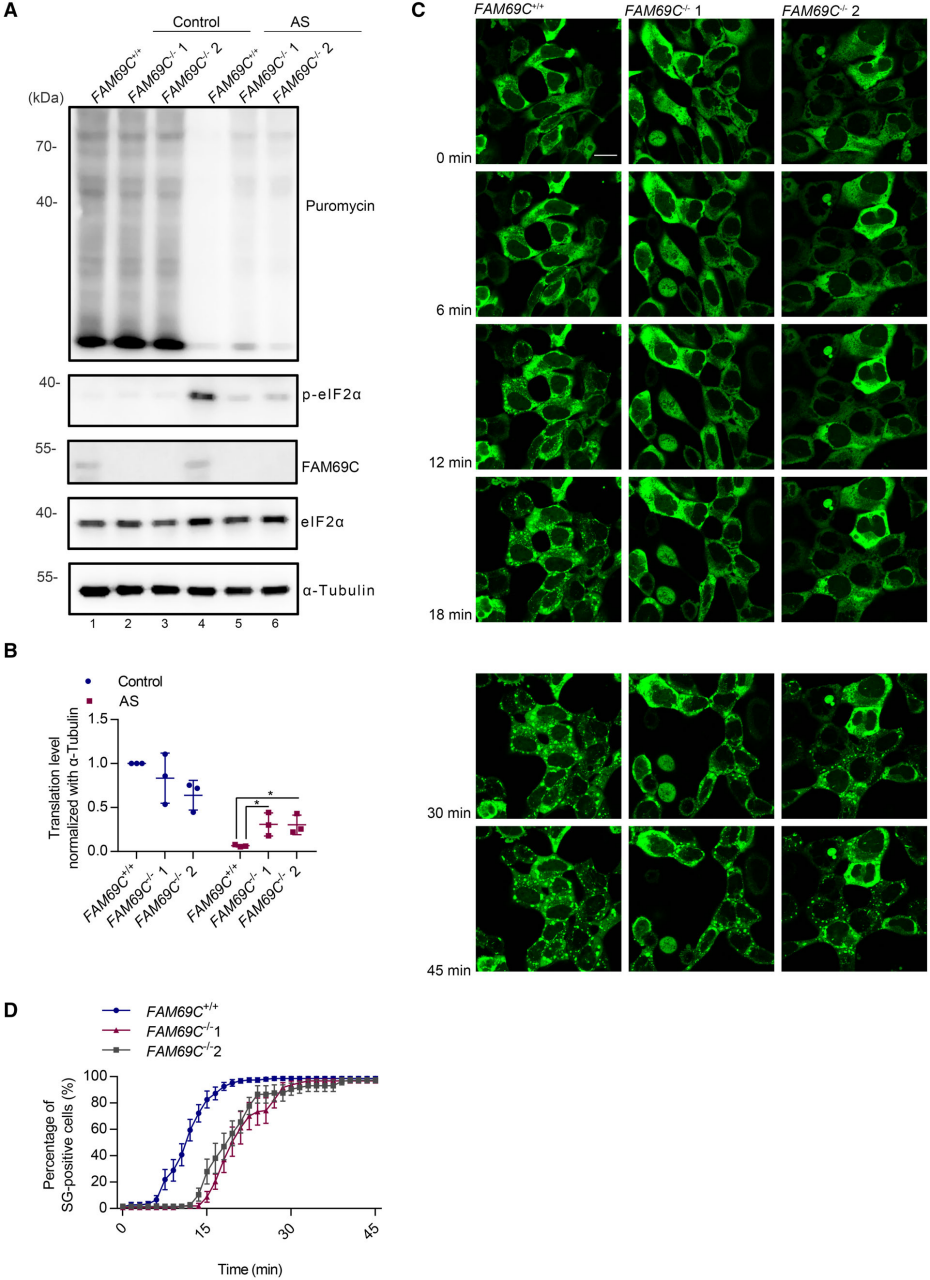
FAM69C mediates stress‐induced translation arrest and promotes stress granule assembly A
Immunoblot analysis of *de novo* synthesized proteins in SH‐SY5Y cells. Puromycin incorporation assay of *FAM69C* ^+/+^ and 2 *FAM69C* ^−/−^ SH‐SY5Y cell lines treated with 0.5 mM AS for 30 min followed by 50 μg/ml puromycin for 15 min. An anti‐puromycin antibody (Puro) was used to visualize *de novo* synthesized proteins. A representative image is shown, *n* = 3 biologically independent experiments.B
Quantitative analysis of puromycin labeled *de novo* synthesized proteins. *P value < 0.05, paired two‐tailed *t*‐test, *n* = 3 biologically independent experiments. Mean ± SD. For *FAM69C* ^+/+^ versus *FAM69C* ^−/−^ 1, *P* value = 0.0416; For *FAM69C* ^+/+^ versus *FAM69C* ^−/−^ 2, *P* value = 0.0346.C
Live‐cell imaging showing the delayed stress granule assembly in *FAM69C* ^−/−^ SH‐SY5Y cells. Time‐lapse images of *FAM69C* ^+/+^ and *FAM69C* ^−/−^ SH‐SY5Y cells stably expressing GFP‐tagged G3BP1 were acquired after cells were exposed to 0.1 mM AS for 45 min. Representative images, *n* > 3 biologically independent experiments. Scale bar = 10 μm.D
Quantification of cells with stress granules at different times. For *FAM69C* ^+/+^, *n* = 8 experiments; *FAM69C* ^−/−^ 1, *n* = 8 experiments; *FAM69C* ^−/−^ 2, *n* = 9 experiments. Mean ± SEM.

Given that four known kinases phosphorylate eIF2α under specific condition, we determined whether FAM69C is a stress‐specific kinase for eIF2α. We examined three conditions that relate to SG assembly, including oxidative stress, heat shock, and ER stress. First, we knocked down *HRI* in the *FAM69C* ^+/+^ and *FAM69C* ^−/−^ SH‐SY5Y cells and tested the levels of phosphorylated eIF2α in response to oxidative stress (Fig EV1C and D). Western blotting showed that knockout of *FAM69C* substantially reduced phosphorylated eIF2α upon AS treatment (Fig EV1C, lane 7 versus lane 8). Further reduction in HRI abolished eIf2α phosphorylation (Fig EV1C, lanes 11, 12 versus lanes 9, 10). These data indicated that both FAM69C and HRI are the physiologically relevant kinases that phosphorylate eIF2α under AS‐induced oxidative stress. Second, we knocked down *PERK* in the *FAM69C* ^+/+^ and *FAM69C* ^−/−^ cells. Upon heat shock, levels of phosphorylated eIF2α were significantly increased in *FAM69C* ^+/+^ cells, as compared with that in *FAM69C* ^−/−^ cells (Fig EV1E, lane 7 versus lane 8). Further, knock down of *PERK* did not impose much effect on the levels of phosphorylated eIF2α (Fig EV1E, lanes 9, 10 versus lanes 11, 12). These data revealed that FAM69C, but not PERK is responsible for phosphorylating eIF2α upon heat shock. Third, we determined the kinase for eIF2α under ER stress. In thapsigargin (TG) treatment, levels of phosphorylated eIF2α were considerably increased, which was independent of FAM69C (Fig EV1E, lane 13 versus lane 14). However, knock down of *PERK* completely inhibited eIF2α phosphorylation (Fig EV1E, lanes 15, 16, 17, 18 versus lanes 13, 14). These data indicated that PERK, but not FAM69C is the responsible kinase for eIF2α under ER stress. Taken together, we propose that FAM69C is a stress‐specific kinase that phosphorylates eIF2α under oxidative stress and heat shock, but not ER stress.

### FAM69C mediates stress‐induced translation arrest and promotes stress granule assembly

Given that FAM69C is a kinase of eIF2α and phosphorylated eIF2α rapidly arrests protein synthesis, we thus examined the role of FAM69C in stress‐induced translation inhibition by RiboPuromycylation assay. In *FAM69C* ^+/+^ SH‐SY5Y cells, western blotting revealed that protein translation was severely inhibited in response to AS treatment (Fig 2A, lane 1 versus lane 4). However, FAM69C deficiency resulted in partially blocked protein translation，which was consistent with reduced levels of phosphorylated eIF2α (Fig 2A, lane 4 versus lanes 5, 6, and Fig 2B). These data indicated that FAM69C deficiency led to aberrant stress‐induced translation inhibition.

**Figure EV2:**
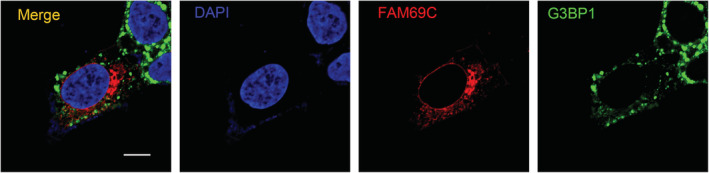
Subcellular localization of FAM69C Confocal imaging of SH‐SY5Y cells transfected with FAM69C and G3BP1, to visualize the localization of FAM69C and stress granules. Scale bar = 10 μm.

Based on the fact that SG assembly parallels stress‐induced translation inhibition, we examined the role of FAM69C on SG formation. We constructed an SH‐SY5Y cell line with stably expressed GFP‐tagged G3BP1 for live‐cell imaging. *FAM69C* ^+/+^ and *FAM69C* ^−/−^ SH‐SY5Y cells were treated with AS to induce SGs, and the formation of SGs was continuously monitored for 45 min. Interestingly, FAM69C deficiency resulted in a significant delay in the rate of SG assembly, as compared to that of *FAM69C* ^+/+^ cells (Fig 2C and D). Immunofluorescence indicated that FAM69C did not colocalize with G3BP1 (Fig EV2A), suggesting that FAM69C was not a component of SG. These data indicated that FAM69C promotes SG assembly through upstream eIF2α signaling.

**Figure 3:**
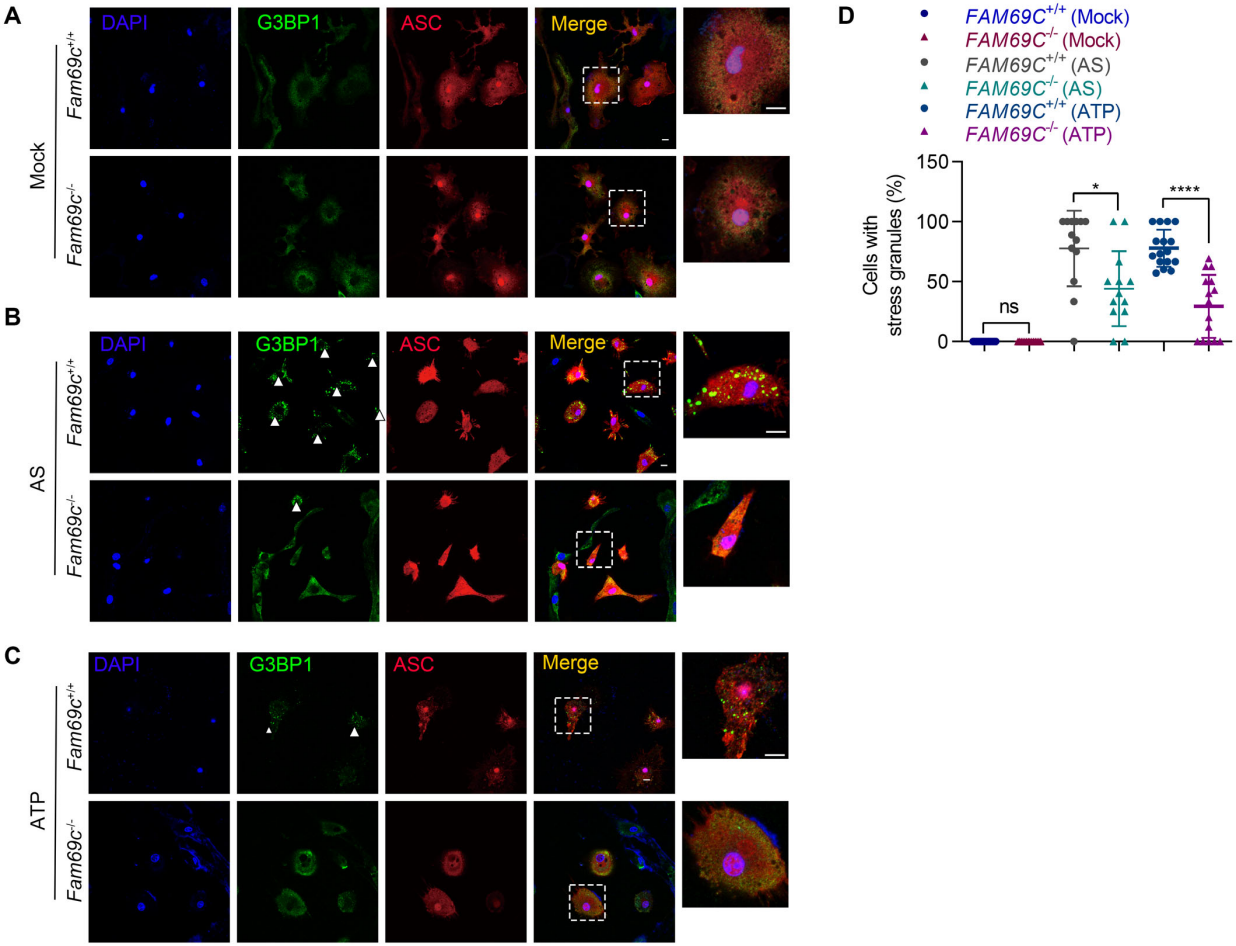
FAM69C facilitates stress granule assembly in microglia A–C
Confocal imaging of primary cultured *Fam69c* ^+/+^ and *Fam69c* ^−/−^ microglia treated with mock, 0.5 mM AS (20 min), and 5 mM ATP (40 min) to visualize the formation of stress granules. *Fam69c* ^−/−^ microglia showed impaired stress granule (indicated by arrowheads) assembly compared with *Fam69c* ^+/+^ microglia when treated with AS and ATP. Representative images, *n* = 3 biologically independent experiments. Scale bar = 10 μm.D
Quantitative analysis of microglia with stress granules. For AS, *P value = 0.0118, unpaired two‐tailed *t*‐test, *n* = 13 fields from three biologically independent experiments. For ATP, ****P value < 0.0001, unpaired two‐tailed *t*‐test, *n* = 15 fields from three biologically independent experiments. Mean ± SD.

### FAM69C facilitates stress granule assembly in microglia

**Figure EV3:**
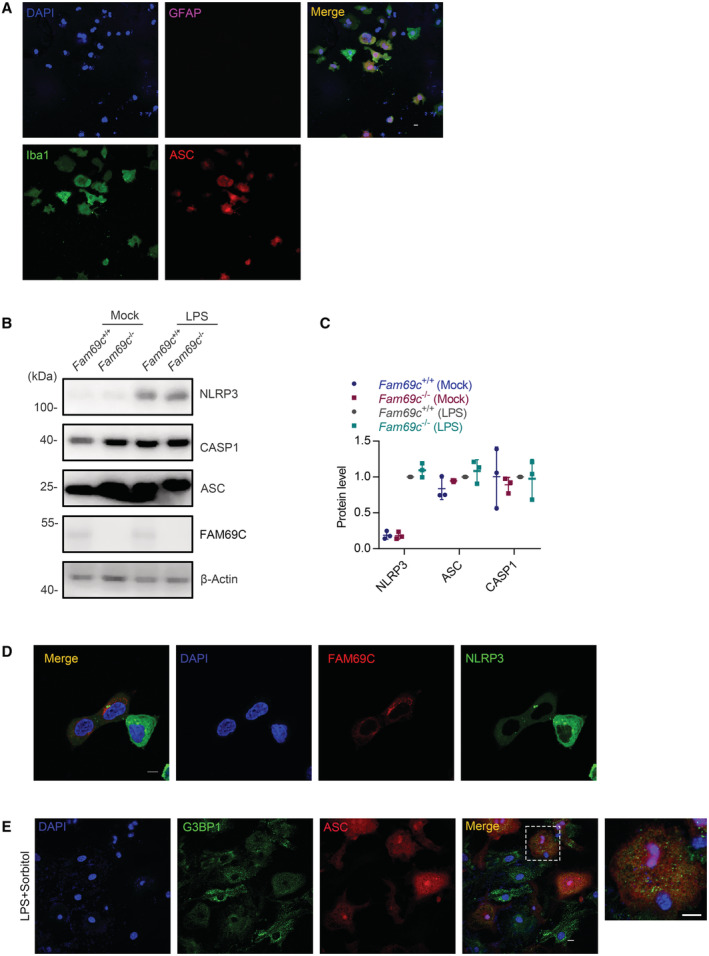
Features in primary culture studies A
Confocal imaging of isolated primary microglia. GFAP (Purple) was used as a marker of astrocyte, and Iba1 (Green) was used as a marker of microglia. All of the Iba1‐positive microglia are ASC‐positive (Red). Scale bar = 10 μm. Representative images, *n* = 8 fields from three biologically independent experiments.B
Immunoblot analysis of protein levels of core NLRP3 inflammasome components in primary cultured *Fam69c* ^+/+^ and *Fam69c* ^−/−^ microglia after LPS priming (1 μg/ml LPS for 3 h). Representative blots, *n* = 3 biologically independent experiments.C
Quantitative analysis of protein level of NLRP3, ASC and CASP1 normalized with β‐Actin in *Fam69c* ^+/+^ and *Fam69c* ^−/−^ microglia. *n* = 3 biologically independent experiments. Mean ± SD.D
Confocal microscopy imaging of SH‐SY5Y cells transfected with FAM69C and NLRP3, to visualize the localization of FAM69C and NLRP3 specks. Scale bar = 10 μm.E
Confocal imaging of primary cultured microglia treated with 1 μg/ml LPS (3 h), followed by 0.4 M sorbitol (1 h), to visualize the formation of stress granules. Representative images, *n* = 3 biologically independent experiments. Scale bar = 10 μm.

Microglia, as the primary immune cell in the brain, actively respond to stress conditions and are linked to a variety of neurodegenerative diseases. We thus determined the role of FAM69C in the regulation of stress granule in microglia. Primary microglia derived from wild‐type and *Fam69c* ^−/−^ mice were treated with a series of conditions (Fig EV3A), and SGs were labeled with endogenous G3BP1. Under the basal condition, there were no SGs in either genotype (Fig 3A). Upon AS treatment, *Fam69c* ^+/+^ microglia form a greater number of SGs, as compared to that in the *Fam69c* ^−/−^ microglia (Fig 3B and D). In addition to oxidative stress, microglia sense damage‐associated ATP through purinoceptors, a process that controls microglial chemotaxis to injury (Li & Barres, 2018). Remarkably, exposure to ATP induces robust SGs formation in *Fam69c* ^+/+^ microglia, whereas less SG assembly was observed in *Fam69c* ^−/−^ microglia (Fig 3C and D). Taken together, we suggest that both AS and ATP induce FAM69C‐dependent SGs formation in microglia.

**Figure 4:**
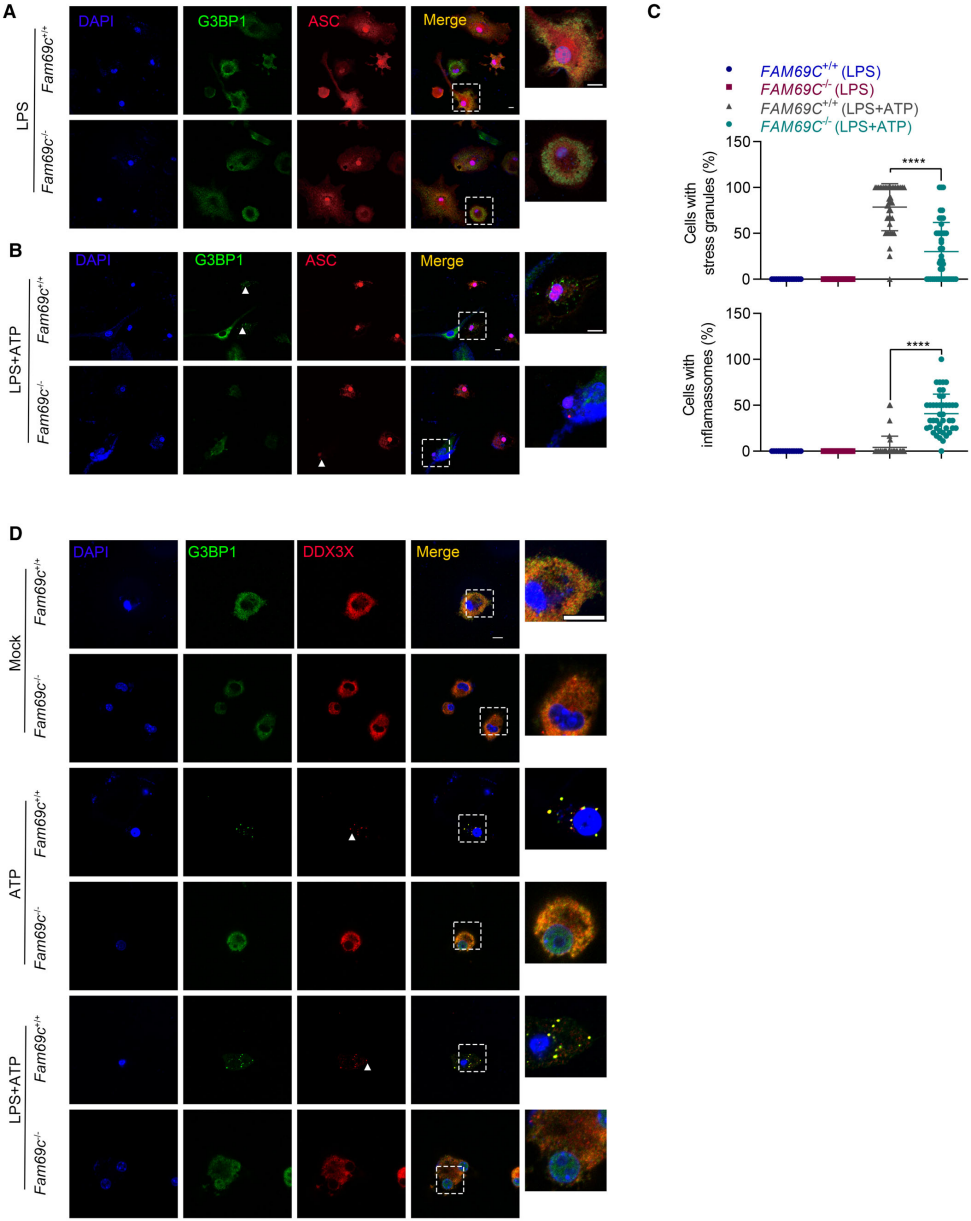
Stress granule assembly in microglia is correlated with reduced NLRP3 inflammasome activation A, B
Confocal imaging of LPS (1 μg/ml, 3 h) primed primary cultured *Fam69c* ^+/+^ and *Fam69c* ^−/−^ microglia treated with or without 5 mM ATP for 40 min to visualize the formation of stress granules (green, indicated by arrowheads) and inflammasomes (red, indicated by arrowheads). LPS‐primed *Fam69c* ^−/−^ microglia showed impaired ATP‐induced stress granule assembly compared with *Fam69c* ^+/+^ microglia when treated with ATP. Meanwhile, more cells with ASC specks were found in *Fam69c* ^−/−^ microglia than that in *Fam69c* ^+/+^ microglia. Representative images, *n* > 3 biologically independent experiments. Scale bar = 10 μm.C
Quantitative analysis of microglia with stress granules and inflammasomes treated with LPS, LPS and ATP. ****P value < 0.0001, unpaired two‐tailed *t*‐test. For LPS, *n* = 15 fields from three biologically independent experiments; For LPS + ATP, *n* = 40 fields from six biologically independent experiments. Mean ± SD.D
Confocal imaging of primary cultured *Fam69c* ^+/+^ and *Fam69c* ^−/−^ microglia treated with mock, 5 mM ATP (40 min), 1 μg/ml LPS (3 h) and 5 mM ATP (40 min) to visualize DDX3X (red, indicated by arrowhead) localization. G3BP1 was used as a marker of stress granules. *Fam69c* ^+/+^ microglia form stress granules and lead to the sequestration of DDX3X when treated with ATP. Scale bar = 10 μm.

### FAM69C‐dependent stress granule assembly in microglia is correlated with reduced NLRP3 inflammasome activation

Given that reversible SG assembly plays a protective role for eukaryotes cells to survive stresses, we examined the cellular effect of SG assembly in microglia. Microglia, a type of brain‐resident macrophage, is activated by misfolded proteins, oxidative stress, and ATP in neurodegenerative diseases and exerts multifaceted functions (Song & Colonna, 2018). The cytosolic innate immune signaling receptor NLRP3 senses these damage‐associated molecular patterns (DAMPs) and mediates inflammasome activation (Heneka *et al*, 2018). Specifically, NLRP3 inflammasome activation involves a priming signal that results in the NF‐κB‐dependent transcriptional upregulation of NLRP3 and pro‐IL‐1β/18, and the following activation signal that induces the oligomerization and activation of NLRP3 (Voet *et al*, 2019). We thus primed microglia with lipopolysaccharide (LPS) and observed no difference in the priming step of NLRP3 activation between *Fam69c* ^+/+^ and *Fam69c* ^−/−^ microglia (Fig EV3B and C). Meanwhile, LPS treatment did not affect SG assembly (Fig 4A). Next, we induced SG formation in microglia by adding ATP after 3 h of LPS priming. A greater number of SGs were found in *Fam69c* ^+/+^ microglia, compared with that in *Fam69c* ^−/−^ microglia. Interestingly, we observed significantly fewer ASC specks in *Fam69c* ^+/+^ microglia than that in *Fam69c* ^−/−^ microglia (Fig 4B and C). These data indicated that FAM69C‐dependent SG formation is correlated with reduced NLRP3 inflammasome activation.

Further, we explored the potential mechanism underlying the negative correlation of SG assembly and NLRP3 inflammasome. First, we determined whether FAM69C was a component of inflammasome. Immunofluorescence showed that FAM69C was not colocalized with NLRP3 (Fig EV3D). As previously reported that sequestration of DDX3X in stress granule prevents its interaction with NLRP3 and inflammasome activation (Samir *et al*, 2019), we determined the status of DDX3X sequestration in *Fam69c* ^+/+^ and *Fam69c* ^−/−^ microglia. As expected, ATP exposure induces SG assembly and DDX3X sequestration in SG in *Fam69c* ^+/+^ microglia, as compared with control (Fig 4D). By contrast, loss of *FAM69C* failed to sequester DDX3X. Consistently, LPS‐primed *Fam69c* ^+/+^ microglia forms SGs with complete sequestration of DDX3X, whereas *Fam69c* ^−/−^ microglia showed no signs of such sequestration (Fig 4D). Thus, we speculate that FAM69C facilitates SG assembly, which sequesters DDX3X to prevent NLRP3 inflammasome activation.

### FAM69C‐independent inflammasome activation

**Figure 5:**
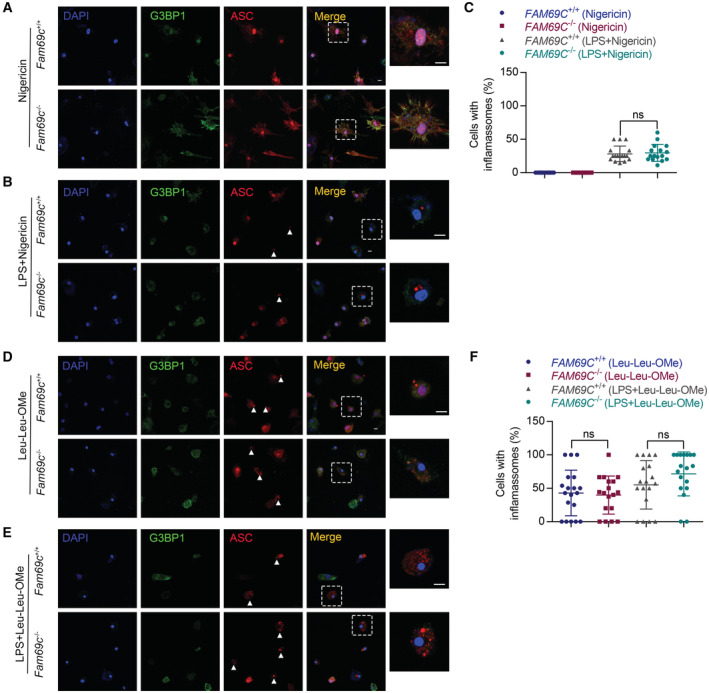
FAM69C‐independent inflammasome activation A, B
Confocal imaging of primary cultured *Fam69c* ^+/+^ and *Fam69c* ^−/−^ microglia treated with 20 μM nigericin (40 min), 1 μg/ml LPS (3 h) and 20 μM nigericin (40 min), to visualize the formation of stress granules and inflammasomes. Microglia form inflammasomes (indicated by arrowheads) and did not form stress granules when treated with nigericin. Representative images, *n* = 3 biologically independent experiments. Scale bar = 10 μm.C
Quantification of cells with inflammasomes. Primary cultured *Fam69c* ^+/+^ and *Fam69c* ^−/−^ microglia were treated with 20 μM nigericin (40 min), 1 μg/ml LPS (3 h) and 20 μM nigericin (40 min). ns = no significant, unpaired two‐tailed *t*‐test, *n* = 16 fields from three biologically independent experiments. Mean ± SD.D, E
Confocal imaging of primary cultured *Fam69c* ^+/+^ and *Fam69c* ^−/−^ microglia treated with 1 mM Leu‐Leu‐Ome (3 h), 1 μg/ml LPS (3 h) and 1 mM Leu‐Leu‐Ome (3 h), to visualize the formation of stress granules and inflammasomes. Microglia form inflammasomes (indicated by arrowheads) and did not form stress granules when treated with Leu‐Leu‐Ome. Representative images, *n* = 3 biologically independent experiments. Scale bar = 10 μm.F
Quantification of cells with stress granules and inflammasomes. Primary cultured *Fam69c* ^+/+^ and *Fam69c* ^−/−^ microglia were treated with 1 mM Leu‐Leu‐Ome (3 h), 1 μg/ml LPS (3 h) and 1 mM Leu‐Leu‐Ome (3 h). ns = no significant, unpaired two‐tailed *t*‐test, *n* = 18 fields from three biologically independent experiments. Mean ± SD.

It has been well characterized that NLRP3 inflammasome is induced by various inducers, through potassium‐dependent and potassium‐independent mechanisms (Mangan *et al*, 2018). Based on the findings that FAM69C regulates ATP‐induced SGs in microglia and correlated with reduced inflammasome activation, we examined the role of FAM69C in other induction conditions. Nigericin is one of the most commonly used inflammasome inducers, which permeabilizes the cell membrane specifically to potassium. Without priming, *Fam69c* ^+/+^ and *Fam69c* ^−/−^ microglia did not show inflammasome activation upon nigericin treatment (Fig 5A). After LPS priming, nigericin induced a comparable number of ASC specks in *Fam69c* ^+/+^ and *Fam69c* ^−/−^ microglia, in the absence of SG formation (Fig 5B and C). To corroborate these findings, we employed the lysosomotropic reagent L‐leucyl‐L‐leucine methyl ester (Leu‐Leu‐OMe) to disrupt lysosomes. Likewise, Leu‐Leu‐OMe did not induce SGs, but activated comparable number of NLRP3 inflammasome independent of *FAM69C* genotype both in the primed and unprimed microglia (Fig 5D–F). Taken together, we proposed that FAM69C‐associated inflammasome inhibition in microglia is dependent on conditions that induce SG assembly. To this end, ATP exposure, but not other inflammasome inducers, is related to the crosstalk between FAM69C‐dependent SG assembly and inflammasome activation in microglia.

### SG assembly precludes NLRP3 inflammasome activation

**Figure 6:**
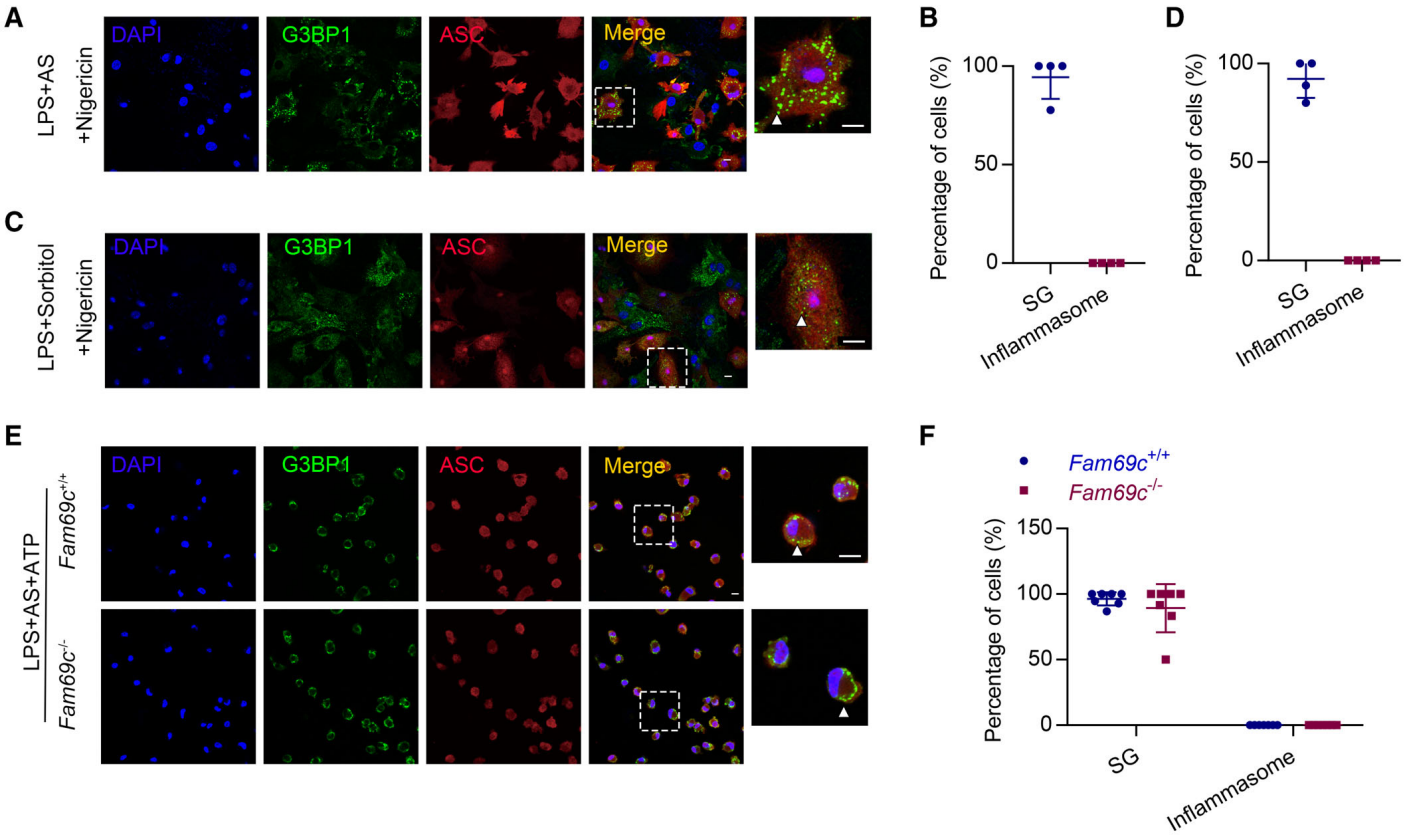
SG assembly precludes NLRP3 inflammasome activation A–D
AS‐ and Sorbitol‐induced stress granules suppressed the formation of nigericin‐induced inflammasomes in microglia. (A, C) Confocal imaging of primary cultured microglia treated with 1 μg/ml LPS (3 h), followed by 0.5 mM AS (40 min) or 0.4 M sorbitol (1 h) and 20 μM nigericin (40 min), to visualize the formation of stress granules (indicated by arrowheads) and inflammasomes. Representative images, *n* = 3 biologically independent experiments. Scale bar = 10 μm. (B, D) Quantification of cells with stress granules and inflammasomes. Primary cultured microglia were treated with LPS + AS + Nigericin (B) and LPS + Sorbitol + Nigericin (D), *n* = 4 fields from two biologically independent experiments. Mean ± SD.E
Confocal imaging of primary cultured *Fam69c* ^+/+^ and *Fam69c* ^−/−^ microglia treated with 1 μg/ml LPS (3 h), followed by 0.5 mM AS (40 min) and 5 mM ATP (40 min), to visualize the formation of stress granules and inflammasomes. AS‐induced stress granules (indicated by arrowheads) suppressed the formation of ATP‐induced inflammasomes in *Fam69c* ^−/−^ microglia. Representative images, *n* = 2 biologically independent experiments. Scale bar = 10 μm.F
Quantification of cells with stress granules and inflammasomes. Primary cultured *Fam69c* ^+/+^ and *Fam69c* ^−/−^ microglia were treated with LPS + AS+ATP. ns = no significant, unpaired two‐tailed *t*‐test, *n* = 7 fields from two biologically independent experiments. Mean ± SD.

To examine whether the correlation between SG assembly and inflammasome inhibition is a general observation, we induced SG assembly in microglia and followed by treatment with the inflammasome inducer. To this end, microglia were primed with LPS, pretreated with AS to induce SG assembly, and followed by nigericin treatment (Fig 6A). Remarkably, SG assembly did preclude NLRP3 inflammasome activation (Fig 6B). Sorbitol, like AS, was able to induce SG assembly in primed microglia (Fig EV3E). Consistently, we found that the presence of sorbitol‐induced SGs inhibited nigericin‐induced inflammasome formation (Fig 6C and D).

Next, we tried to rescue exacerbated inflammasome activation in *Fam69c* ^−/−^ microglia. We prolonged the AS treatment from 20 to 40 min and followed by ATP treatment. Prolonged AS treatment was sufficient for the finalization of delayed SG assembly in *Fam69c* ^−/−^ microglia (Fig 6E). With the presence of SGs, inflammasome activation represented by ASC specks was completely suppressed in *Fam69c* ^−/−^ microglia (Fig 6F). Taken together, we proposed that SG assembly precludes NLRP3 inflammasome formation.

### Aged FAM69C‐deficient mice show inflammasome activation

As the predominant type of innate immune cells in the brain, microglia are the main source of proinflammatory cytokines. Cytokines released by microglia are key mediators of neuroinflammation, which can induce and regulate a wide range of cellular responses (Colonna & Butovsky, 2017). The homeostasis of microglia is thus critical for normal brain function. Moreover, proinflammatory cytokines lead to dysfunctional microglia and dampen the clearance of misfolded proteins in neurodegenerative disease.

**Figure 7:**
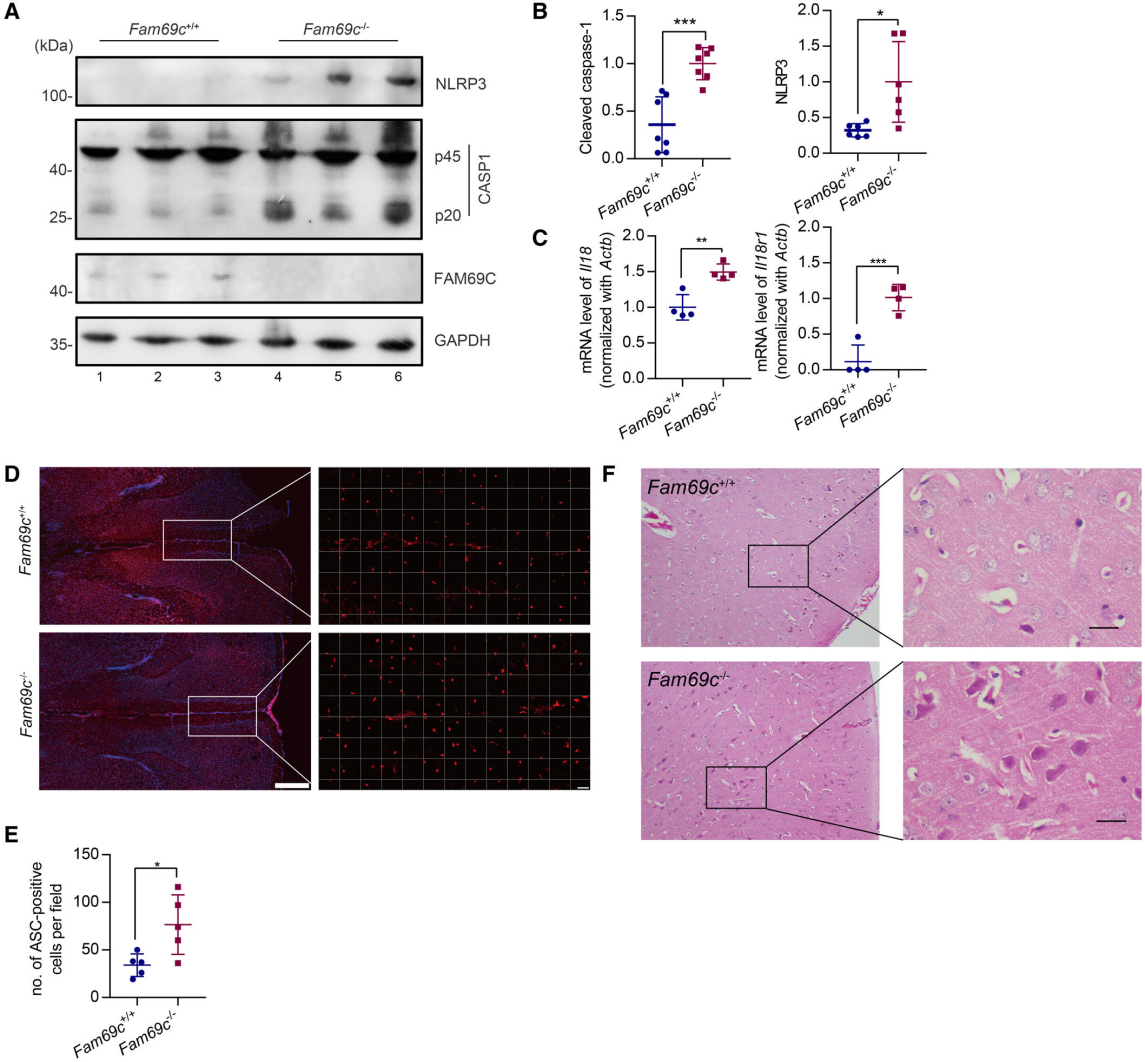
Aged FAM69C‐deficient mice show inflammasome activation A
Immunoblot analysis of NLRP3 and cleaved caspase1 (p20) in cortex of 12‐month‐old *Fam69c* ^+/+^ and *Fam69c* ^−/−^ mice. Data are from three different mice with each genotype.B
Quantification of NLRP3 and cleaved caspase1 (p20) normalized with GAPDH in the cortex of 12‐month‐old *Fam69c* ^+/+^ and *Fam69c* ^−/−^ mice. For caspase1 (p20), ***P value = 0.0003, *n* = 7 mice/genotype; For NLRP3, *P value = 0.0159, *n* = 6 mice/genotype. Unpaired two‐tailed *t*‐test, Mean ± SD.C
Quantification of *Il18* and *Il18r1* expression normalized with *Actb* in the cortex of 12‐month‐old *Fam69c* ^+/+^ and *Fam69c* ^−/−^ mice. ***P value = 0.0009; **P value = 0.0035, unpaired two‐tailed *t*‐test, *n* = 4 mice/group. Mean ± SD.D
Representative image of ASC staining of *Fam69c* ^+/+^ and *Fam69c* ^−/−^ mouse mPFC. 12‐month‐old *Fam69c* ^+/+^ and *Fam69c* ^−/−^ mice were sacrificed for Frozen sections and ASC immunofluorescence. The density of ASC‐positive cells in the mPFC of *Fam69c* ^−/−^ mice was higher than *Fam69c* ^−/−^ mice. Scale bars, 500 μm (left), 50 μm (right). Representative images (*n* = 5).E
Quantitative analysis of ASC‐positive cells in mPFC of *Fam69c* ^+/+^ and *Fam69c* ^−/−^ mouse with Image J software. *P value = 0.0215, unpaired two‐tailed *t*‐test, *n* = 5 mice/ genotype. Mean ± SD.F
Hematoxylin and eosin staining of 10‐month‐old *Fam69c* ^+/+^ and *Fam69c* ^−/−^ mouse brain. *Fam69c* ^−/−^ mice showed more eosinophilic neurons in the cortex than *Fam69c* ^−/−^ mice. The image is representative of three pairs of mice. Scale bar = 25 μm.

**Figure EV4:**
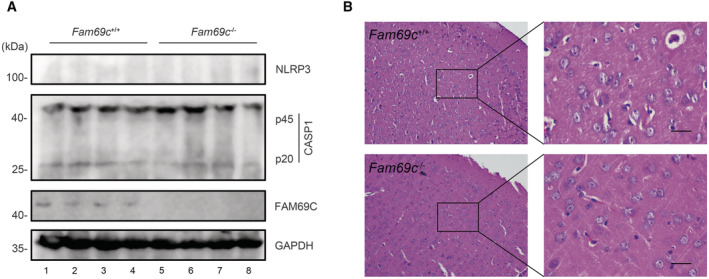
Features in young mice A
Immunoblot analysis of NLRP3 and cleaved caspase1 (p20) in cortex of 3‐month‐old *Fam69c* ^+/+^ and *Fam69c* ^−/−^ mice. Data are from four different mice with each genotype.B
Hematoxylin and eosin staining of 3‐month‐old *Fam69c* ^+/+^ and *Fam69c* ^−/−^ mouse brain. The image is representative of three pairs of mice. Scale bar = 25 μm.

Based on these findings in primary microglia derived from *Fam69c* ^+/+^ and *Fam69c* ^−/−^ mice, we determined the effect of FAM69C in microglia‐mediated inflammation *in vivo*. Given that aging parallels the induction of inflammation, we classified mice into 3‐month‐old young group and 12‐month‐old aged group. Cortical tissues from the aged *Fam69c* ^−/−^ mice showed significantly increased NLRP3 levels and caspase‐1 cleavage, indicating inflammasome activation (Fig 7A and B). Moreover, we examined caspase‐1‐mediated proteolytic activation of cytokines. qRT‐PCR demonstrated that the expression of *Il18* and its receptor *Il18r1* were significantly upregulated in the aged *Fam69c* ^−/−^ mice (Fig 7C). Further, immunofluorescence revealed an increased number of ASC‐expression microglia in the medial prefrontal cortex (mPFC) of aged *Fam69c* ^−/−^ mice than that from *Fam69c* ^+/+^ mice (Fig 7D and E). Consistently, we observed a greater number of eosinophilic neurons in the cortex of aged *Fam69c* ^−/−^ mice, as compared with the *Fam69c* ^+/+^ mice (Fig 7F). By contrast, no inflammasome activation was observed in the 3‐month‐old young *Fam69c* ^−/−^ mice (Fig EV4A). HE staining showed no evident difference between *Fam69c* ^−/−^ mice and age‐matched young *Fam69c* ^+/+^ mice (Fig EV4B). These data indicated that FAM69C deficiency together with aging parallels microglia‐associated inflammasome activation.

Taken together, these data suggested that aged FAM69C‐deficient mice show inflammasome activation.

## Discussion

In our previous study, we identified that FAM69C is a brain‐enriched kinase linked to neurodegenerative diseases (Mei *et al*, 2022). The substrates of FAM69C and its biological function are largely unknown. Here, we found that FAM69C functions as an eIF2α kinase and promotes stress granule assembly. Specifically, we characterized the impact of FAM69C‐dependent stress granule assembly on microglia function. This study suggests the potential protective role of FAM69C in aging and neurodegenerative diseases.

**Figure EV5:**
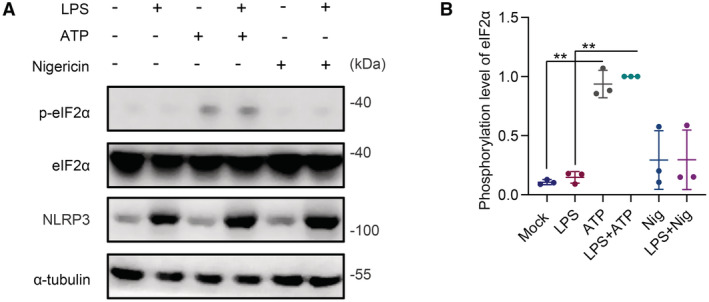
ATP‐induced phosphorylation of eIF2α in BV2 cells A
Immunoblot analysis of phosphorylated eIF2α in BV2 cells. Primed (1 μg/ml LPS for 3 h) or unprimed BV2 cells were treated with mock, 5 mM ATP or 20 μM nigericin for 40 min, and lysates were immunoblotted with p‐eIF2α (S51) antibody. Representative blots, *n* = 3 biologically independent experiments.B
Quantitative analysis of the phosphorylation level of eIF2α normalized with total eIF2α. **P value < 0.01, paired two‐tailed *t*‐test, *n* = 3 biologically independent experiments. Mean ± SD. For ATP versus mock, *P* value = 0.0047; For LPS + ATP versus LPS, *P* value = 0.0011.

The cellular stress response has a vital role in the regulation of brain homeostasis (Farley & Watkins, 2018). eIF2α is a critical molecule in stress responses, related to integrated stress response and stress granule assembly. Phosphorylation of eIF2α suppresses general protein translation and subsequently promotes SG assembly, which is composed of condensates of RNA‐binding proteins and untranslated messenger RNAs. Here, we found that ATP is able to induce SG assembly in microglia. We provided evidence that ATP treatment leads to increased levels of phosphorylated eIF2α. By contrast, the inflammasome inducer nigericin was not able to induce phosphorylation of eIF2α (Fig EV5A and B). Previous studies have employed both ATP and nigericin as inflammasome inducers in macrophages (Iyer *et al*, 2009; Samir *et al*, 2019). Here, we showed the difference between ATP and nigericin in the activation of eIF2α signaling and induction of SG assembly in microglia. Further, we provided evidence that FAM69C functions as a novel stress‐specific kinase for eIF2α, in addition to the four known eIF2α kinases. We delineate the role of FAM69C in the regulation of SG assembly through eIF2α signaling.

Previous studies have shown that SG assembly and inflammasome activation determine contrasting live‐or‐die fates of stressed macrophages (Samir *et al*, 2019). As for the molecular mechanisms underlying the interplay between SG assembly and inflammasome activation, two molecules have been well studied. HSP 90 and DDX3X are conserved components of SGs, but also play a direct role in NLRP3 inflammasome activation. Specifically, sequestration of HSP90 and DDX3X in SGs inhibits the formation of NLRP3 inflammasomes (Mayor *et al*, 2007; Samir *et al*, 2019). In our study, FAM69C facilitates ATP‐induced stress granule assembly in microglia, which leads to the sequestration of DDX3X in SG. Meanwhile, we observed decreased inflammasome activation in *Fam69c* ^+/+^ microglia as compared with *Fam69c* ^−/−^ microglia. These data suggest that FAM69C may indirectly regulate inflammasome activation through DDX3X. Further, we provided evidence that induction of SG assembly in *Fam69c* ^−/−^ microglia rescued exacerbated inflammasome activation. Our work illustrates that FAM69C promotes SG assembly in microglia and regulates microglia function, albeit its impact on reduced inflammasome activation is not direct.

Activation of microglia contributes to neuroinflammation (Hammond *et al*, 2019). Pathological misfolded protein aggregates and DAMPs released from dead neurons can activate microglia through inflammasomes. Innate immunity contributes to the development of neurodegenerative diseases. Inflammasome activation in *Fam69c* ^−/−^ mice was manifested by increased NLRP3 expression, caspase‐1 cleavage, and *Il18* expression. Changes of another key interleukin IL‐1β involved in inflammasome activation were not seen in qRT‐PCR, which may be due to the relatively lower expression of *Il1b* than *Il18*. In the future study, we may isolate microglia, rather than the whole tissue lysates from the aged *Fam69c* ^−/−^ brain, and measure the expression of *Il1b* .

In conclusion, we discovered that the brain‐enriched kinase FAM69C promotes SG assembly through the phosphorylation of eIF2a. Stress‐induced stress granule assembly in microglia precludes NLRP3 inflammasome activation. These findings reveal a new molecular mechanism for the initiation of stress granules and demonstrate the role of FAM69C in the regulation of microglia function.

## Materials and methods

### Animals and cell lines

All animals were maintained in a special pathogen‐free facility, and the animal study protocols used were approved by the ethics committee of Peking University Health Science Center (approval number LA2017142). The 293T, Hela, and SH‐SY5Y cell lines were obtained from the American Type Culture Collection. The BV2 microglial cell line was purchased from Procell Co., Ltd. and has been authenticated recently (#CL‐0493, Wuhan, China).

### *Fam69c* ^−/−^ mice and *FAM69C* ^−/−^ cell line

*Fam69c* ^−/−^ mice and *FAM69C* ^−/−^ SH‐SY5Y cell line used in this study were generated using the CRISPR design tool, which has been described previously by Mei *et al* (2022).

### Antibodies

The following antibodies were used in this study: ASC (#67824, CST), α‐tubulin (#RM2007, Ray antibody Biotech), β‐actin (#PM053, MBL), Caspase‐1 (p20) (AG‐20B‐0042, AdipoGen), DDX3X (ab271002, Abcam), eIF2α (#5324, CST), Phospho‐eIF2α (Ser51) (#3398, CST), FAM69C (home‐made), FLAG (#F3165, Sigma‐Aldrich), G3BP1 (sc‐365338, Santacruz), GAPDH (#RM2002, Ray antibody Biotech), GFAP (#12389, CST), GFP (#RM1008, Ray antibody Biotech), GM130 (#2296, CST), HA (#H3663, Sigma‐Aldrich), HRI (sc‐365239, Santacruz), Iba1 (019–19741, Wako), PERK (#5683, CST), Puromycin (MABE341, Millipore).

### Co‐immunoprecipitation

Cells were lysed with Co‐IP lysis buffer (150 mM NaCl, 1 mM EDTA, 20 mM Tris–HCl pH 8.0, 0.5% NP40, and 0.1 mM PMSF). Then, the cell lysates were incubated with S‐protein agarose (Novagen) for 90 min. The beads were washed with 0.3% NP40 three times and eluted with SDS‐PAGE loading buffer (50 mM Tris–HCl pH 6.8, 2% SDS, 0.1% bromophenol blue, 10% glycerol, and 5% 2‐mercaptoethanol) by boiling for 10 min, followed by immunoblot analysis.

### Establishment of stable knockdown cell lines

To generate stable HRI and PERK‐silenced cells, shRNA against HRI and PERK were cloned into pLKO.1 Vector. HEK293T cells were cotransfected with pLKO.1 recombinant construct and the packaging plasmids pMD2.G and psPAX2 for 48 h. Media were collected and filtered with a 0.22 μm filter. The cell culture supernatants containing lentivirus were used to infect *FAM69C* ^+/+^ and *FAM69C* ^−/−^ SH‐SY5Y cell line. After infection for 48 h, the stable silenced cells were screened with 2 μg/ml puromycin. The target sequences by shRNAi are listed below:

shHRI‐1, AGCTACTTTGCCAGACGTTTA;
shHRI‐2, GGATTGGATAGTCGAGAGAAA;
shPERK‐1, CCGTAGTAAGAAATGGATCAT;
shPERK‐2, GCACACAGATTACAGTCAGAT.

### HE staining and ASC staining

Mice were sacrificed for HE staining and ASC staining. The paraffin‐embedded brain was sectioned into 5 μm slices, followed by HE staining. For ASC staining, frozen sections were used. The sections were blocked in 5% BSA, 0.3% Triton X‐100 and PBS (blocking buffer) for 30 min followed by 3 h of room temperature incubation with the ASC antibody (#67824, CST) in the blocking buffer. Sections were washed three times in PBS and incubated with Alexa Fluor 555 conjugated secondary antibodies for 60 min, followed by DAPI for 10 min. To quench autofluorescence, the sections were incubated for 20 min in 0.1% Sudan Black B in 75% ethanol and washed extensively in PBS.

### Immunoblot

For immunoblot, cultured cells were lysed in 0.5% NP40 lysis buffer, and mouse tissues were lysed in RIPA buffer (50 mM Tris–HCl [pH 7.4], 150 mM NaCl, 2 mM EDTA, 1% NP‐40, 0.5% sodium deoxycholate, 0.1% SDS, 100 mM PMSF). For phosphorylated protein detection, PIC inhibitor (Roche) and phosphatase inhibitor (10 mM NaF, 1 mM Na_3_VO_4_) were added additionally.

Proteins were separated by SDS‐PAGE and blotted on PVDF membranes. The membranes were blocked with 5% nonfat milk in PBS‐T (1% Tween 20)/TBS‐T (20 mM Tris, Ph8.0, 150 mM NaCl with 0.5% Tween 20) for 1 h at RT and incubated with the indicated antibodies overnight at 4°C. After washing with PBS‐T/TBS‐T buffer three times, membranes were incubated with HRP‐conjugated secondary antibodies for 1 h at RT.

### Immunofluorescence

Cells seeded on glass coverslips were fixed with 4% PFA at room temperature for 15 min, followed by blocking in 1% bovine serum albumin containing 0.3% tritonX‐100 at room temperature for 1 h, and incubation in the diluted primary antibody at 4°C overnight. After being incubated with secondary antibodies at room temperature for 30 min and stained with 0.5 μg/ml DAPI for 10 min, coverslips were mounted with fluorescence decay resistant medium. Images were acquired with a Nikon TCS A1 microscope.

### *In vitro* phosphorylation assay

10 μg recombinant eIF2α protein was mixed with or without 1 μg FAM69C protein in reaction buffer (40 mM Tris–HCl (pH7.5), 20 mM MgCl2, 1 mM ATP) at 37° for 30 min.

FAM69C was obtained as described by Mei (Mei *et al*, 2022). For recombinant expression of eIF2α, DNA fragments encoding human eIF2α were cloned into a pET‐28a (+) vector with His tag on the N terminal. BL21 cells transfected with eIF2α were treated with 0.1 mM IPTG for 20 h and then lysed by ultrasonic. eIF2α was enriched with Ni Beads.

### Live‐cell imaging

Cells stably expressing GFP‐tagged G3BP1 were established for live‐cell imaging. *FAM69C* ^+/+^ or *FAM69C* ^−/−^ cells were seeded on cell culture dishes (Bioptechs Inc) 24 h before imaging. The heater was used to keep the temperature at 37°C. Multipoint images were taken every 90 s with the 488 nm laser after 0.1 mM AS treatment immediately.

### Membrane and cytoplasm isolation

HEK 293T Cells expressing C‐terminal GFP tagged FAM69C were grown to confluency in a 150 mm dish and treated with or without 0.5 mM sodium arsenite (AS) for 1 h. Membrane and cytoplasm were isolated with Applygene P1201. For density gradient centrifugation, cells were lysed in CER buffer (Applygene P1201) for 15 s. The lysate was then centrifuged for 5 min at 800 *g*. 1 ml Supernatant was layered on a linear sucrose gradient (15–45%, 400 μl/layer, 11 layer), centrifuged at 100,040 *g* in a Beckman sw55Ti rotor at 4°C for 40 min. Fractions 1–10 (500 μl each) were harvested from the top to the bottom. 15 μl from each fraction was analyzed by western blot. Fraction 1 represents the lowest density fraction, and fraction 10 represents the highest density.

### Primary microglia culture

Newborn mice were sacrificed for mixed glial culture, and primary glial cells were further cultured in DMEM supplemented with 10% FCS, 100 U/ml penicillin/streptomycin, and 20 ng/ml GM‐CSF. Microglial cells were used after 14 days of primary cultivation. To assess the formation of ASC specks, LPS‐primed microglia were activated with nigericin (20 μM) or ATP (5 mM) for 40 min. Cells were then fixed for further immunofluorescence assays.

### qRT‐PCR

mRNA from mice brains was reverse transcribed into cDNA with All‐In‐One RT MasterMix (Abm), and quantitative real‐time polymerase chain reaction (qPCR) was carried out on a 7,500 Fast real‐time PCR system.

The following primers for the *interleukin‐18 (Il18)*, *interleukin‐18 receptor 1 (Il18r1)*, *interleukin‐1 beta (Il1b)*, *interleukin‐1 receptor type 1 (Il1r1)*, and *Beta‐actin (Actb)* genes were used:

*Il18*: sense 5′‐GACTCTTGCGTCAACTTCAAGG‐3′, antisense 5′‐ CAGGCTGTCTTTTGTCAACGA‐3′;
*Il18r1*: sense 5′‐ACTTTTGCTGTGGAGACGTTAC‐3′, antisense 5′‐ CCGGCTTTTCTCTATCAGTGAAT‐3′;
*Il1b*: sense 5′‐GAAATGCCACCTTTTGACAGTG‐3′, antisense 5′‐TGGATGCTCTCATCAGGACAG‐3′;
*Il1r1*: sense 5′‐ GGGAAGCAATATCCGGTCACA‐3′, antisense 5′‐TGACGTTGCAGATCAGTTGTATC‐3′;
*Actb*: sense 5′‐GGCTGTATTCCCCTCCATCG‐3′, antisense 5′‐CCAGTTGGTAACAATGCCATGT‐3′.

### RiboPuromycylation

*FAM69C* ^+/+^ and *FAM69C* ^−/−^ cells were treated with or without 0.5 mM AS for 30 min, followed by puromycin (50 μg/ml) treatment for 15 min. Puromycin‐labeled proteins were identified with immunoblot.

### S‐tag pull‐down assay

SH‐SY5Y cells stably expressing S‐HA‐tagged FAM69C were grown to confluency in a 150 mm dish for S‐tag pull‐down. Cells were lysed for 30 min in 0.5% NP40 lysis buffer, and FAM69C was recognized and pulled down by the S‐protein Agarose beads. The FAM69C‐bounded S‐protein Agarose beads were washed 3 times with 0.3% NP40 buffer, followed by a mass spectrometry assay. ClueGO‐v2.5.8 (Bindea *et al*, 2009) was used for GO enrichment analysis of potential interacting proteins.

### Transfections

Transient transfections were performed with polyethylenimine (PEI) and Opti‐MEM. For stable transfection construction, psPAX2, pMD2.G, and pCDH‐CMV‐MCS‐EF1‐puro‐G3BP1‐GFP were cotransfected into HEK293T to produce lentivirus. Cell culture supernatants containing lentivirus were collected, filtered, and then added into *FAM69C* ^+/+^ and *FAM69C* ^−/−^ SH‐SY5Y cell line. After infection for 48 h, the stable cell lines were screened by puromycin.

## Disclosure and competing interests statement

The authors declare that they have no conflict of interest.

## Supporting information



Dataset EV1

Expanded View Figures PDF

PDF+

## Data Availability

The data set (and computer code) produced in this study is available in the following database: Mass spectrometry data: Proteomics Identification Database PXD037697 (https://www.ebi.ac.uk/pride/archive/projects/PXD037697). Any additional information required to reanalyze the data reported in this paper is available from the lead contact upon request.
