# Supplementary material for: FAM69C functions as a kinase for eIF2α and promotes stress granule assembly
Source: EMBO Rep. 2023 Mar 16;24(5):e55641. doi: 10.15252/embr.202255641 (PMC10157376; doi:10.15252/embr.202255641)
Supplement: Supplementary file 2 — Expanded View Figures PDF [file EMBR-24-e55641-s004.pdf]

Expanded View Figures

Figure EV1. FAM69C is a stress-specific kinase for eIF2α.

A Immunoblot analysis of phosphorylated eIF2α in SH-SY5Y cells. FAM69C$^{-/-}$ SH-SY5Y cells were transfected to express wildtype FAM69C and FAM69C D279N. Cells were treated with 0.5 mM AS for 30 min, and lysates were immunoblotted with the p-eIF2α (S51) antibody.

B Coomassie Blue Staining showed the protein level of BSA, FAM69C and D279N proteins used in the ADP-Glo kinase assay.

C Immunoblot analysis of phosphorylated eIF2α in HRI-knockdown SH-SY5Y cells. Cells were treated with or without 0.5 mM AS for 30 min, and lysates were immunoblotted with p-eIF2α (S51), eIF2α, HRI, and α-Tubulin antibody.

D Quantitative analysis of the phosphorylation level of eIF2α normalized with total eIF2α under AS treatment. *P value < 0.05, paired t-test, n = 3 biologically independent experiments. Mean ± SD. For *FAM69C*$^{-/-}$ shHRI-1, P value = 0.0431; For *FAM69C*$^{-/-}$ shHRI-2, P value = 0.0337.

E Immunoblot analysis of phosphorylated eIF2α in PERK-knockdown SH-SY5Y cells. Cells were treated with 42°C heat shock (30 min), 4 μM TG (6 h). The cell lysates were immunoblotted with p-eIF2α (S51), eIF2α, PERK, and α-Tubulin antibody.

 © 2023 The Authors

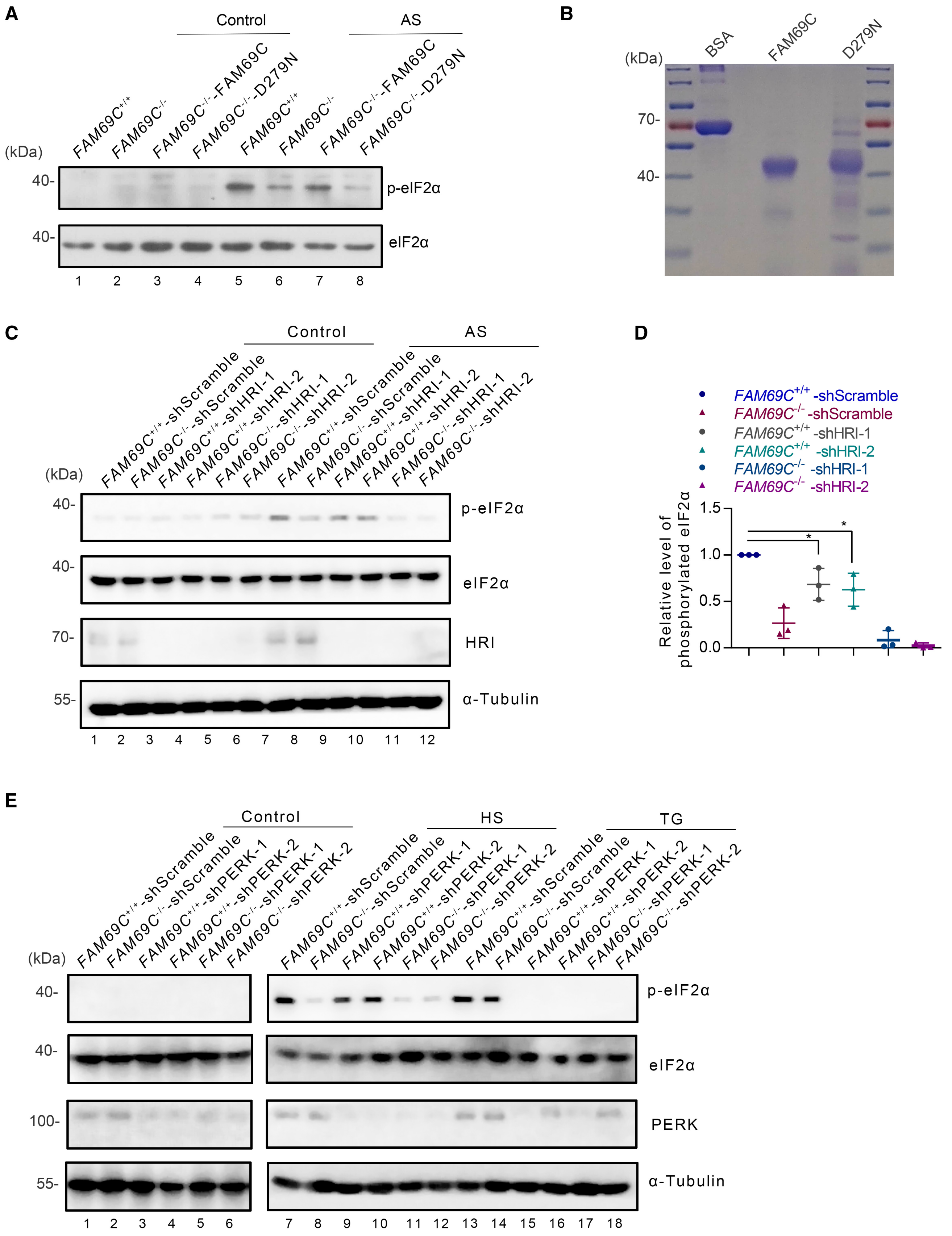
A
Control
AS
(kDa)
FAM69C+/+
FAM69C-/-
FAM69C-/--FAM69C
FAM69C-/--D279N
40-
p-eIF2α
eIF2α
1 2 3 4 5 6 7 8
B
(kDa)
BSA
FAM69C
D279N
70-
40-
C
Control
AS
FAM69C+/+-shScramble
FAM69C-/--shScramble
FAM69C+/+-shHRI-1
FAM69C+/+-shHRI-2
FAM69C-/--shHRI-1
FAM69C-/--shHRI-2
p-eIF2α
eIF2α
HRI
α-Tubulin
70-
55-
1 2 3 4 5 6 7 8 9 10 11 12
D
FAM69C+/+ -shScramble
FAM69C-/- -shScramble
FAM69C+/+ -shHRI-1
FAM69C+/+ -shHRI-2
FAM69C-/- -shHRI-1
FAM69C-/- -shHRI-2
Relative level of phosphorylated eIF2α
1.5
1.0
0.5
0.0
*
E
Control
HS
TG
FAM69C+/+-shPERK-1
FAM69C+/+-shPERK-2
FAM69C-/--shPERK-1
FAM69C-/--shPERK-2
PERK
100-
1 2 3 4 5 6 7 8 9 10 11 12 13 14 15 16 17 18

Figure EV1.

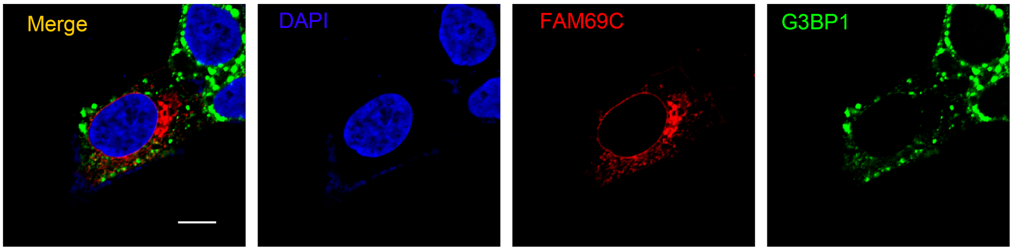

Figure EV2. Subcellular localization of FAM69C.

Confocal imaging of SH-SY5Y cells transfected with FAM69C and G3BP1, to visualize the localization of FAM69C and stress granules. Scale bar = 10 µm.

Figure EV3. Features in primary culture studies.

A Confocal imaging of isolated primary microglia. GFAP (Purple) was used as a marker of astrocyte, and Iba1 (Green) was used as a marker of microglia. All of the Iba1-positive microglia are ASC-positive (Red). Scale bar = 10 µm. Representative images, $n = 8$ fields from three biologically independent experiments.

B Immunoblot analysis of protein levels of core NLRP3 inflammasome components in primary cultured *Fam69c*$^{+/+}$ and *Fam69c*$^{-/-}$ microglia after LPS priming (1 µg/ml LPS for 3 h). Representative blots, $n = 3$ biologically independent experiments.

C Quantitative analysis of protein level of NLRP3, ASC and CASP1 normalized with β-Actin in *Fam69c*$^{+/+}$ and *Fam69c*$^{-/-}$ microglia. $n = 3$ biologically independent experiments. Mean ± SD.

D Confocal microscopy imaging of SH-SY5Y cells transfected with FAM69C and NLRP3, to visualize the localization of FAM69C and NLRP3 specks. Scale bar = 10 µm.

E Confocal imaging of primary cultured microglia treated with 1 µg/ml LPS (3 h), followed by 0.4 M sorbitol (1 h), to visualize the formation of stress granules. Representative images, $n = 3$ biologically independent experiments. Scale bar = 10 µm.

 © 2023 The Authors

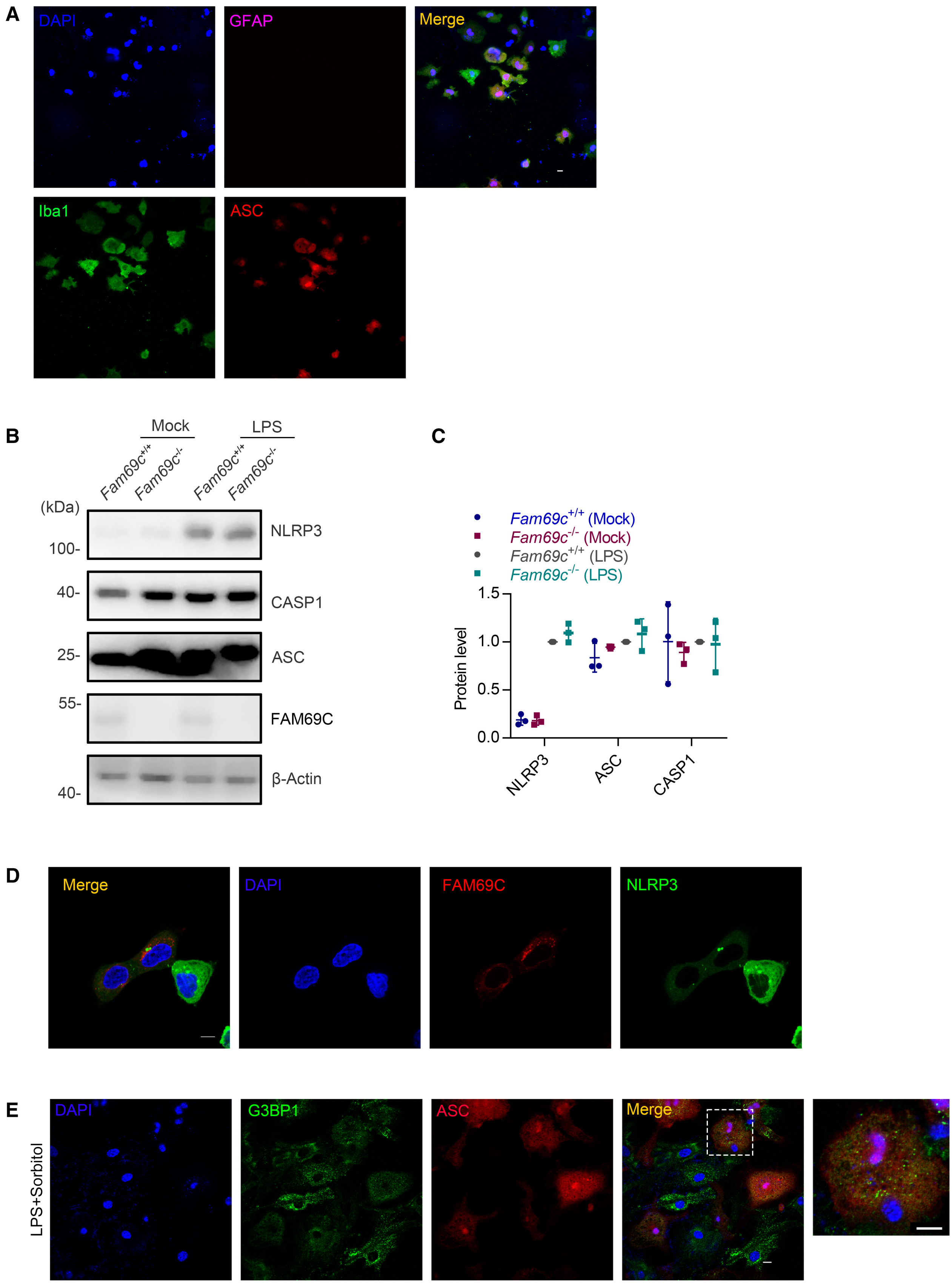
A
DAPI
GFAP
Merge
Iba1
ASC
B
Mock
LPS
Fam69c+/+
Fam69c-/-
Fam69c+/+
Fam69c-/-
(kDa)
100-
NLRP3
40-
CASP1
25-
ASC
55-
FAM69C
β-Actin
40-
C
Fam69c+/+ (Mock)
Fam69c-/- (Mock)
Fam69c+/+ (LPS)
Fam69c-/- (LPS)
Protein level
1.5
1.0
0.5
0.0
NLRP3
ASC
CASP1
D
Merge
DAPI
FAM69C
NLRP3
E
LPS+Sorbitol
DAPI
G3BP1
ASC
Merge

Figure EV3.

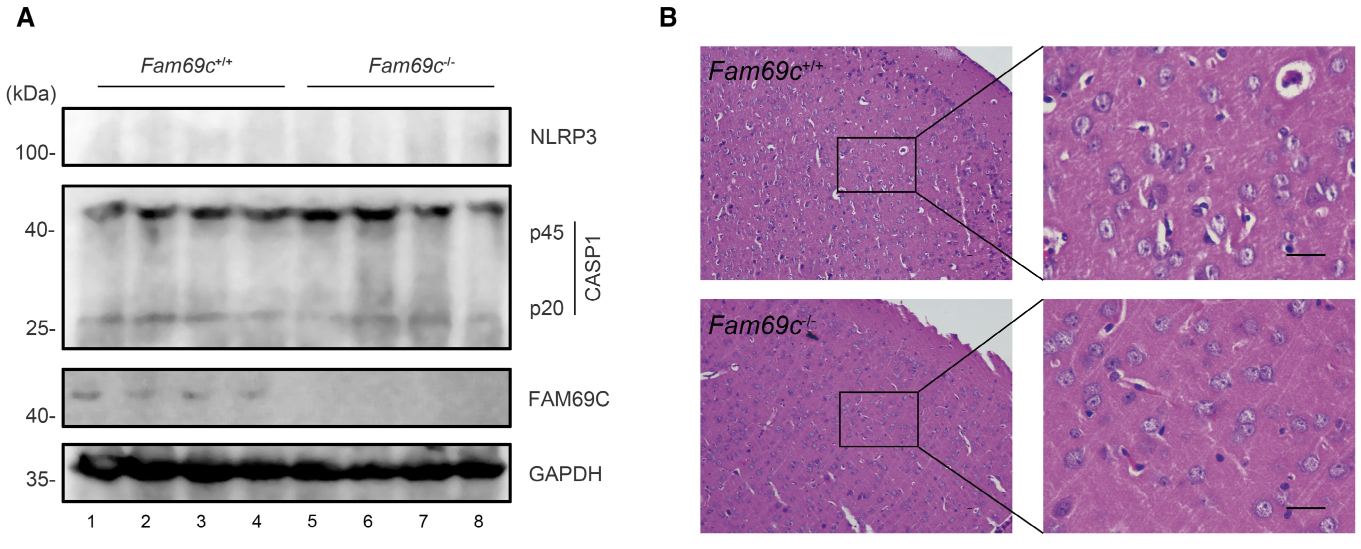

Figure EV4. Features in young mice.

A Immunoblot analysis of NLRP3 and cleaved caspase1 (p20) in cortex of 3-month-old *Fam69c*$^{+/+}$ and *Fam69c*$^{-/-}$ mice. Data are from four different mice with each genotype.

B Hematoxylin and eosin staining of 3-month-old *Fam69c*$^{+/+}$ and *Fam69c*$^{-/-}$ mouse brain. The image is representative of three pairs of mice. Scale bar = 25 μm.

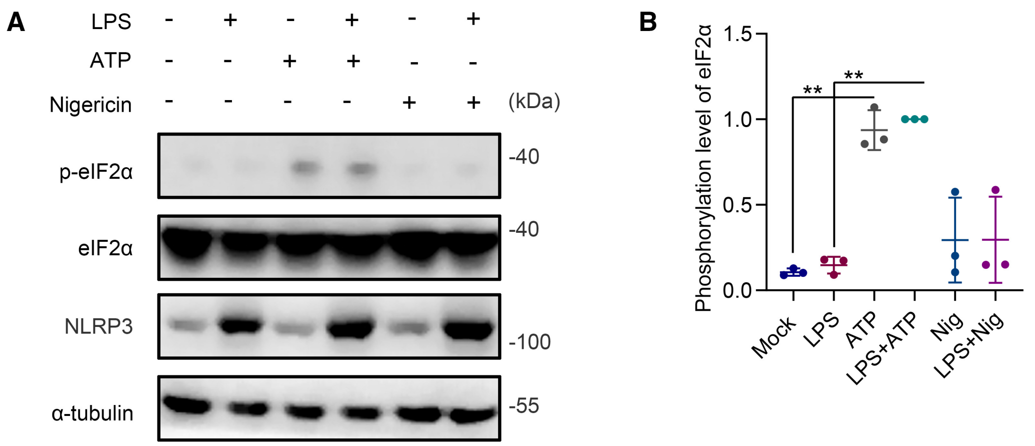

Figure EV5. ATP-induced phosphorylation of eIF2α in BV2 cells.

A Immunoblot analysis of phosphorylated eIF2α in BV2 cells. Primed (1 μg/ml LPS for 3 h) or unprimed BV2 cells were treated with mock, 5 mM ATP or 20 μM nigericin for 40 min, and lysates were immunoblotted with p-eIF2α (S51) antibody. Representative blots, $n = 3$ biologically independent experiments.

B Quantitative analysis of the phosphorylation level of eIF2α normalized with total eIF2α. $^{**}P$ value < 0.01, paired two-tailed *t*-test, $n = 3$ biologically independent experiments. Mean ± SD. For ATP versus mock, P value = 0.0047; For LPS + ATP versus LPS, P value = 0.0011.

 © 2023 The Authors
