# Supplementary material for: FAM69C functions as a kinase for eIF2α and promotes stress granule assembly
Source: EMBO Rep. 2023 Mar 16;24(5):e55641. doi: 10.15252/embr.202255641 (PMC10157376; doi:10.15252/embr.202255641)
Supplement: Supplementary file 3 — PDF+ [file EMBR-24-e55641-s001.pdf]

Article

TRANSPARENT PROCESS

OPEN ACCESS

EMBO reports

FAM69C functions as a kinase for eIF2α and promotes stress granule assembly

Zhongyan Wu[1,†], Fan Mei[1,†], Yangyang Gan[1], Anhang Liu[1], Jiapan Hu[1,2], Yan Jin[1] & Yuxin Yin[1,2,*]

Abstract

Stress granules are dynamic cytoplasmic ribonucleoprotein granules that assemble in response to cellular stress. Aberrant formation of stress granules has been linked to neurodegenerative diseases. However, the molecular mechanisms underlying the initiation of stress granules remain elusive. Here we report that the brain-enriched protein kinase FAM69C promotes stress granule assembly through phosphorylation of eukaryotic translation initiation factor 2 (eIF2α). FAM69C physically interacts with eIF2α and functions as a stress-specific kinase for eIF2α, leading to stress-induced protein translation arrest and stress granule assembly. Primary microglia derived from *Fam69c* knockout mice exhibit aberrant stress granule assembly in response to oxidative stress and ATP. Defective stress granule assembly in microglia correlates with the formation of ASC specks and NLRP3 inflammasome activation, whereas induction of stress granule precludes inflammasome formation. Consistently, increased NLRP3 levels, caspase-1 cleavage and *Il18* expression corroborate microglia-associated neuroinflammation in aged *Fam69c* knockout mice. Our study demonstrates that FAM69C is critical for stress granule assembly and suggests its role in the regulation of microglia function.

Keywords eIF2α; FAM69C; inflammasome; microglia; stress granule
Subject Categories Immunology; Neuroscience; Signal Transduction
DOI 10.15252/embr.202255641 | Received 24 June 2022 | Revised 8 February 2023 | Accepted 16 February 2023 | Published online 16 March 2023
EMBO Reports (2023) 24: e55641
See also: **A Ruggieri** (May 2023)

Introduction

Translational control in eukaryotic cells is critical for cell survival during stress responses (Sonenberg & Hinnebusch, 2009). Regulation of protein translation is imposed at the initiation stage. The phosphorylation of the α-subunit of eukaryotic translation initiation factor 2 (eIF2α) decides the integrity of the preinitiation complex and translation initiation (Ivanov *et al*, 2019). There are four known kinases that are responsible for the phosphorylation of eIF2α under specific conditions, including PKR-like ER kinase (PERK), double-stranded RNA-dependent protein kinase (PKR), heme-regulated eIF2α kinase (HRI), and general control non-derepressible protein 2 (GCN2) (Pakos-Zebrucka *et al*, 2016).

Stress granules (SGs) form when translation initiation is inhibited during a variety of stress responses, including heat shock (HS), nutrient deprivation, hypo-osmotic conditions, and oxidative stress (Protter & Parker, 2016). SGs have been proposed to stabilize mRNAs, regulate mRNA translation and have impact on physiological and pathological processes (Buchan & Parker, 2009). SG assembly entraps viral mRNAs and inhibits viral gene expression in antiviral immune response (McCormick & Khaperskyy, 2017). Phosphorylation of eIF2α and SG assembly promote the survival of cells under stress (Aulas *et al*, 2017). In tumor cells, SG assembly inhibits mTORC1 and apoptosis through SG component astrin (Thedieck *et al*, 2013). SG assembly protects neuron and other long-lived cells from death under stress (Farley & Watkins, 2018). Mutation of *FUS* (Baron *et al*, 2013; Bosco *et al*, 2010) and expanded *C9ORF72* hexanucleotide repeat (Boeynaems *et al*, 2017) in neurodegenerative diseases are associated with defects in the disassembly of SGs, resulting in the phosphorylation of cytosolic TDP43 and inhibition of nuclear import (Gasset-Rosa *et al*, 2019; Wolozin & Ivanov, 2019).

Neurodegeneration is characterized by progressive loss of neuron activity and function, ultimately leading to neuron death. It is well known that pathologic protein aggregation triggers neurodegeneration in neurodegenerative disease. Amyloidosis, tauopathies, a-synucleinopathies, and TDP-43 proteinopathies represent the predominant type of pathological hallmarks (Dugger & Dickson, 2017; Vaquer-Alicea & Diamond, 2019). Pathologic protein aggregates have been considered to be the cause of neurodegeneration, while the immune changes in neurodegenerative diseases were considered as a secondary or reactive response to protein aggregates and neurodegeneration. With the development of sequencing technology, the microglial-specific gene *TREM2* R47H mutation identified with genome-wide association studies (GWASs) suggest that dysregulated immune responses contribute to the disease etiology (Jonsson *et al*, 2013).

1 Institute of Systems Biomedicine, Department of Pathology, School of Basic Medical Sciences, Peking University Health Science Center, Beijing, China
2 Institute of Precision Medicine, Peking University Shenzhen Hospital, Shenzhen, China
*Corresponding author. Tel: +(86) 10 8280 1237; Fax: +(86) 10 8280 1380; E-mail: yinyuxin@hsc.pku.edu.cn
†These authors contributed equally to this work

© 2023 The Authors. Published under the terms of the CC BY 4.0 license

Microglia are the main resident innate immune cells in the central nervous system (CNS). Like macrophages, microglia activation can be categorized as either classical (M1) or alternative (M2), representing the proinflammatory and anti-inflammatory states of microglia (Colonna & Butovsky, 2017). Microglia not only recognize pathogen associated molecular patterns (PAMPs) but also can be activated by danger-associated molecular patterns (DAMPs) or protein aggregates (Labzin *et al*, 2018). Extracellular misfolded and aggregated proteins can activate microglia through pattern recognition receptors. Activation of Toll-like receptors (TLRs) triggers NF-κB-dependent proinflammatory gene expression and the induction of proinflammatory cytokines (Lehnardt, 2010). Moreover, microglia express various purinergic receptors for ATP, such as P2Y12 and P2X4, to detect cell injuries (Colonna & Butovsky, 2017). NLRP3 inflammasome mediates M1 macrophage polarization (Zhang *et al*, 2020) and has been found in various neurodegenerative diseases including Alzheimer's disease (Heneka *et al*, 2015), frontotemporal dementia (McCauley & Baloh, 2019), and Parkinson's disease (Marogianni *et al*, 2020). NLRP3 inflammasomes induce the cleavage of caspase-1, which is responsible for the maturation and secretion of the inflammatory cytokines Interleukin-1β (IL-1β) and IL-18, and additional pyroptosis (Kanneganti *et al*, 2006; Voet *et al*, 2019). Moreover, previous studies implicated that NLRP3 inflammasomes are essential for the development and progression of amyloid-β (Aβ) pathology and tau pathology in mouse models (Venegas *et al*, 2017; Ising *et al*, 2019).

As the predominant types of stress responses, crosstalk between stress granule and NLRP3 inflammasome has been studied. On one hand, inflammation upregulated eIF2α phosphorylation and stress granule formation in heat-shocked intestinal epithelial cells (Hu *et al*, 2010), whereas anti-inflammatory cytokine treatment reversed eIF2α phosphorylation and SGs formation (Herman *et al*, 2019). On the other hand, SG assembly inhibits the formation of NLRP3 inflammasomes through the sequestration of HSP90 and DDX3X in SGs (Mayor *et al*, 2007; Samir *et al*, 2019). Additionally, SG assembly directly affects phagocytosis of microglia through the sequestration of SYK (Ghosh & Geahlen, 2015).

In our previous studies, we demonstrated that FAM69C is a brain-enriched kinase that regulates synaptic plasticity and memory and is associated with neurodegenerative dementia (Mei *et al*, 2022). In this study, we explore the function of FAM69C as an eIF2α kinase that promotes stress granule assembly and suggest its role in the regulation of microglia function.

Results

FAM69C is a stress-specific kinase for eIF2α

In our previous work, we characterized a brain-enriched kinase FAM69C and demonstrated that FAM69C deficiency is associated with neurodegenerative dementia. To unveil the biological function of FAM69C, we performed S-tag pulldown assay to identify its interactive proteins. Mass spectrometry analyses identified a total of 1,355 potential candidates associated with FAM69C protein (as assessed by > 5-fold intensity as compared to HA-S-tag control, Dataset EV1). Biological pathway analyses indicated that these candidates were enriched in pathways relative to stress granule (SG) assembly, regulation of translation in response to stress, ribonucleoprotein complex assembly, and cytoplasmic translation (Fig 1A). These results indicate that FAM69C is potentially involved in stress responses. Notably, *EIF2S1*-encoded eIF2α protein is a key component of the preinitiation complex that initiates protein translation (Wolozin & Ivanov, 2019), and the reversible phosphorylation of eIF2α mediates stress-induced protein synthesis arrest and SG assembly (McCormick & Khaperskyy, 2017). Thus, we speculated that eIF2α was a critical molecule that coupled regulation of translation in response to stress and SG assembly, the two FAM69C-associated biological pathways. Next, we examined the physical interaction of FAM69C and eIF2α. Under the basal condition, immunoprecipitation in SH-SY5Y cells indicated that the endogenous eIF2α interacted with FAM69C. Further, we treated SH-SY5Y cells with SG inducer sodium arsenite (AS) and observed that the interaction of FAM69C and eIF2α was significantly strengthened (Fig 1B and C).

Based on the evident interaction between eIF2α and FAM69C, we next determined whether eIF2α might be a substrate of FAM69C. We treated *FAM69C*$^{+/+}$ and *FAM69C*$^{-/-}$ SH-SY5Y cells with AS or heat shock (HS), which were the two conditions that strictly depend on eIF2α for SG induction. Notably, western blotting revealed that levels of phosphorylated eIF2α at S51 in *FAM69C*$^{+/+}$ cells were significantly increased upon AS or HS treatment, whereas loss of *FAM69C* deteriorated eIF2α phosphorylation (Fig 1D and E). By contrast, the D279N mutant, in which the kinase activity was impaired by substituting the critical amino acid Asp 279 in the catalytic loop with Asn, reduced eIF2α phosphorylation (Fig EV1A). Further, we determined whether FAM69C directly phosphorylates eIF2α using recombinant FAM69C$^{44–419}$ and full-length eIF2α proteins. In the *in vitro* phosphorylation assay, FAM69C protein directly phosphorylated eIF2α, as indicated by western blotting (Fig 1F). Consistently, the ADP-Glo kinase assay corroborated that FAM69C phosphorylates eIF2α through its kinase activity (Figs 1G and EV1B). These data indicated that FAM69C functions as a kinase for eIF2α.

Given that four known kinases phosphorylate eIF2α under specific condition, we determined whether FAM69C is a stress-specific kinase for eIF2α. We examined three conditions that relate to SG assembly, including oxidative stress, heat shock, and ER stress. First, we knocked down *HRI* in the *FAM69C*$^{+/+}$ and *FAM69C*$^{-/-}$ SH-SY5Y cells and tested the levels of phosphorylated eIF2α in response to oxidative stress (Fig EV1C and D). Western blotting showed that knockout of *FAM69C* substantially reduced phosphorylated eIF2α upon AS treatment (Fig EV1C, lane 7 versus lane 8). Further reduction in HRI abolished eIf2α phosphorylation (Fig EV1C, lanes 11, 12 versus lanes 9, 10). These data indicated that both FAM69C and HRI are the physiologically relevant kinases that phosphorylate eIF2α under AS-induced oxidative stress. Second, we knocked down *PERK* in the *FAM69C*$^{+/+}$ and *FAM69C*$^{-/-}$ cells. Upon heat shock, levels of phosphorylated eIF2α were significantly increased in *FAM69C*$^{+/+}$ cells, as compared with that in *FAM69C*$^{-/-}$ cells (Fig EV1E, lane 7 versus lane 8). Further, knock down of *PERK* did not impose much effect on the levels of phosphorylated eIF2α (Fig EV1E, lanes 9, 10 versus lanes 11, 12). These data revealed that FAM69C, but not PERK is responsible for phosphorylating eIF2α upon heat shock. Third, we determined the kinase for eIF2α under ER stress. In thapsigargin (TG) treatment, levels of phosphorylated eIF2α were considerably increased, which was independent of FAM69C (Fig EV1E, lane 13 versus lane 14). However, knock down of *PERK* completely

 © 2023 The Authors

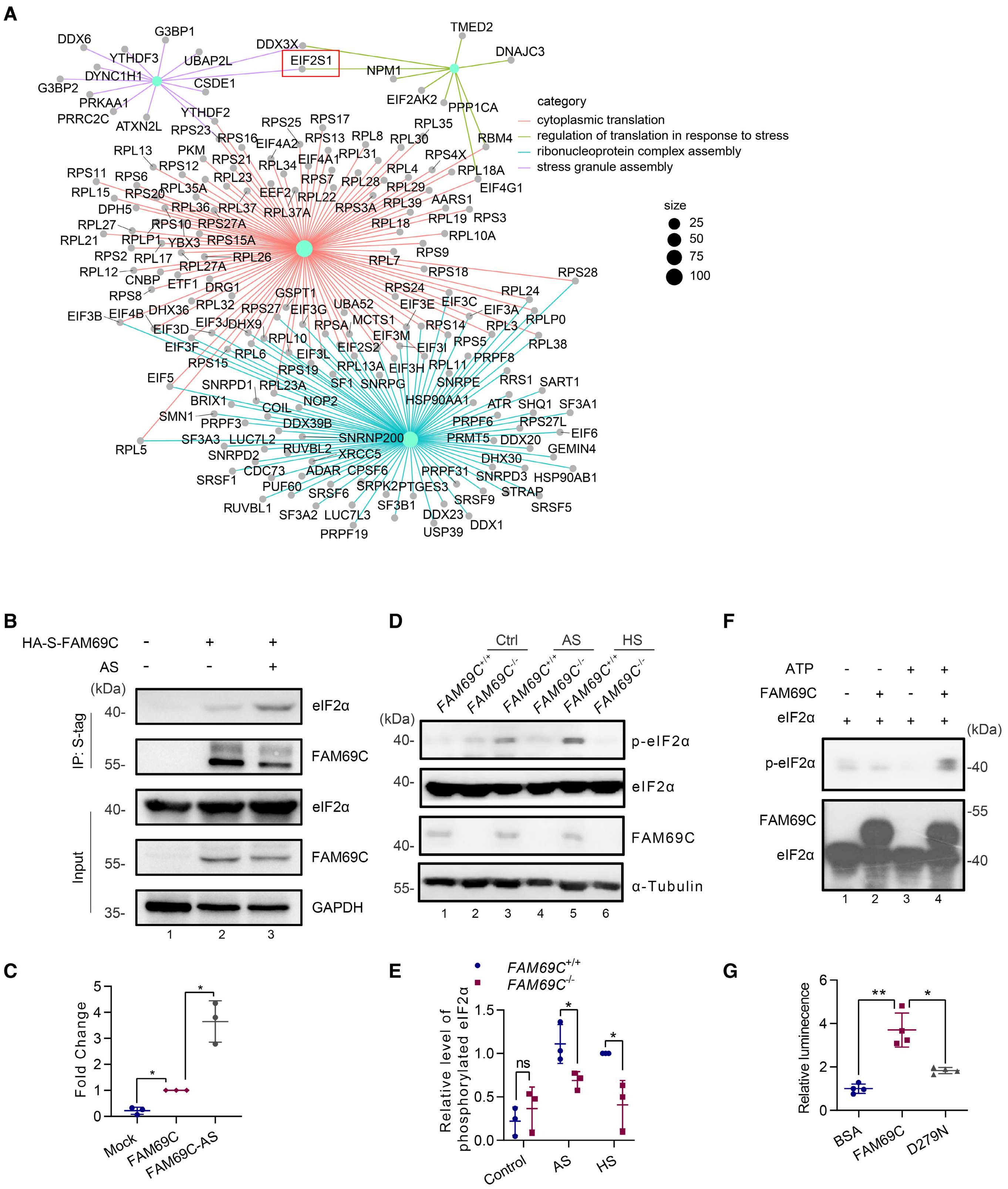
A
category
cytoplasmic translation
regulation of translation in response to stress
ribonucleoprotein complex assembly
stress granule assembly
size
25
50
75
100
EIF2S1
B
HA-S-FAM69C
AS
(kDa)
IP: S-tag
Input
eIF2α
FAM69C
GAPDH
C
Fold Change
Mock
FAM69C
FAM69C-AS
D
Ctrl
AS
HS
p-eIF2α
eIF2α
FAM69C
α-Tubulin
E
Relative level of phosphorylated eIF2α
Control
AS
HS
ns
F
ATP
FAM69C
eIF2α
p-eIF2α
G
Relative luminecence
BSA
FAM69C
D279N

Figure 1.

Figure 1. FAM69C is a stress-specific kinase for eIF2α.

A Cnetplot of enriched pathways for 'cytoplasmic translation', 'regulation of translation in response to stress', 'ribonucleoprotein complex assembly' and 'stress granule assembly'. S-tag pull-down of FAM69C in SH-SY5Y cells and consequent mass spectrometry identified a series of potential proteins interacting with FAM69C.

B Immunoblot analysis of immunoprecipitates with endogenous eIF2α and HA-S-tagged FAM69C. SH-SY5Y cells were transfected to express HA-S-FAM69C, followed by 0.5 mM AS treatment for 30 min. HA-S-FAM69C was pulled-downed with S-protein beads, and the S-protein immunoprecipitants were immunoblotted for eIF2α and HA. Representative blots, $n = 3$ biologically independent experiments.

C Quantitative analysis of eIF2α in the FAM69C Pull-down assay. **P* value < 0.05, paired two-tailed *t*-test, $n = 3$ biologically independent experiments. Mean ± SD. For FAM69C versus mock, *P* value = 0.0103; For FAM69C versus FAM69C-AS, *P* value = 0.0289.

D Immunoblot analysis of phosphorylated eIF2α in *FAM69C*$^{+/+}$ and *FAM69C*$^{-/-}$ SH-SY5Y cells. Cells were treated with 0.5 mM AS, 42°C heat shock for 30 min, and lysates were immunoblotted with p-eIF2α (S51) antibody. Representative blots, $n = 3$ biologically independent experiments.

E Quantitative analysis of the phosphorylation level of eIF2α normalized with total eIF2α. **P* value < 0.05, paired two-tailed *t*-test, $n = 3$ biologically independent experiments. Mean ± SD. For AS, *P* value = 0.0196; For HS, *P* value = 0.0335.

F Immunoblot analysis of phosphorylated eIF2α in the *in vitro* phosphorylation assay. 10 μg recombinant eIF2α protein was mixed with or without 1 μg FAM69C protein in reaction buffer (40 mM Tris–HCl (pH7.5), 20 mM $MgCl_2$, 1 mM ATP) at 37°C for 30 min. Phosphorylated eIF2α was detected with the p-eIF2α (S51) antibody. Representative blots, $n = 3$ biologically independent experiments.

G The relative luminescence in ADP-Glo kinase assay. ADP Generated by FAM69C and the kinase-dead mutant D279N in the *in vitro* phosphorylation assay was quantified with ADP-Glo kinase assay, BSA was used as a control. ***P* value = 0.0084, **P* value = 0.0239, paired two-tailed *t*-test, $n = 4$ biologically independent experiments. Mean ± SD.

inhibited eIF2α phosphorylation (Fig EV1E, lanes 15, 16, 17, 18 versus lanes 13, 14). These data indicated that PERK, but not FAM69C is the responsible kinase for eIF2α under ER stress. Taken together, we propose that FAM69C is a stress-specific kinase that phosphorylates eIF2α under oxidative stress and heat shock, but not ER stress.

FAM69C mediates stress-induced translation arrest and promotes stress granule assembly

Given that FAM69C is a kinase of eIF2α and phosphorylated eIF2α rapidly arrests protein synthesis, we thus examined the role of FAM69C in stress-induced translation inhibition by RiboPuromycylation assay. In *FAM69C*$^{+/+}$ SH-SY5Y cells, western blotting revealed that protein translation was severely inhibited in response to AS treatment (Fig 2A, lane 1 versus lane 4). However, FAM69C deficiency resulted in partially blocked protein translation, which was consistent with reduced levels of phosphorylated eIF2α (Fig 2A, lane 4 versus lanes 5, 6, and Fig 2B). These data indicated that FAM69C deficiency led to aberrant stress-induced translation inhibition.

Based on the fact that SG assembly parallels stress-induced translation inhibition, we examined the role of FAM69C on SG formation. We constructed an SH-SY5Y cell line with stably expressed GFP-tagged G3BP1 for live-cell imaging. *FAM69C*$^{+/+}$ and *FAM69C*$^{-/-}$ SH-SY5Y cells were treated with AS to induce SGs, and the formation of SGs was continuously monitored for 45 min. Interestingly, FAM69C deficiency resulted in a significant delay in the rate of SG assembly, as compared to that of *FAM69C*$^{+/+}$ cells (Fig 2C and D). Immunofluorescence indicated that FAM69C did not colocalize with G3BP1 (Fig EV2A), suggesting that FAM69C was not a component of SG. These data indicated that FAM69C promotes SG assembly through upstream eIF2α signaling.

FAM69C facilitates stress granule assembly in microglia

Microglia, as the primary immune cell in the brain, actively respond to stress conditions and are linked to a variety of neurodegenerative diseases. We thus determined the role of FAM69C in the regulation of stress granule in microglia. Primary microglia derived from wild-type and *Fam69c*$^{-/-}$ mice were treated with a series of conditions (Fig EV3A), and SGs were labeled with endogenous G3BP1. Under the basal condition, there were no SGs in either genotype (Fig 3A). Upon AS treatment, *Fam69c*$^{+/+}$ microglia form a greater number of SGs, as compared to that in the *Fam69c*$^{-/-}$ microglia (Fig 3B and D). In addition to oxidative stress, microglia sense damage-associated ATP through purinoceptors, a process that controls microglial chemotaxis to injury (Li & Barres, 2018). Remarkably, exposure to ATP induces robust SGs formation in *Fam69c*$^{+/+}$ microglia, whereas less SG assembly was observed in *Fam69c*$^{-/-}$ microglia (Fig 3C and D). Taken together, we suggest that both AS and ATP induce FAM69C-dependent SGs formation in microglia.

FAM69C-dependent stress granule assembly in microglia is correlated with reduced NLRP3 inflammasome activation

Given that reversible SG assembly plays a protective role for eukaryotes cells to survive stresses, we examined the cellular effect of SG assembly in microglia. Microglia, a type of brain-resident macrophage, is activated by misfolded proteins, oxidative stress, and ATP in neurodegenerative diseases and exerts multifaceted functions (Song & Colonna, 2018). The cytosolic innate immune signaling receptor NLRP3 senses these damage-associated molecular patterns (DAMPs) and mediates inflammasome activation (Heneka *et al*, 2018). Specifically, NLRP3 inflammasome activation involves a priming signal that results in the NF-κB-dependent transcriptional upregulation of NLRP3 and pro-IL-1β/18, and the following activation signal that induces the oligomerization and activation of NLRP3 (Voet *et al*, 2019). We thus primed microglia with lipopolysaccharide (LPS) and observed no difference in the priming step of NLRP3 activation between *Fam69c*$^{+/+}$ and *Fam69c*$^{-/-}$ microglia (Fig EV3B and C). Meanwhile, LPS treatment did not affect SG assembly (Fig 4A). Next, we induced SG formation in microglia by adding ATP after 3 h of LPS priming. A greater number of SGs were found in *Fam69c*$^{+/+}$ microglia, compared with that in *Fam69c*$^{-/-}$ microglia. Interestingly, we observed significantly fewer ASC specks in *Fam69c*$^{+/+}$ microglia than that in *Fam69c*$^{-/-}$ microglia (Fig 4B and C). These data indicated that FAM69C-dependent SG formation is correlated with reduced NLRP3 inflammasome activation.

Further, we explored the potential mechanism underlying the negative correlation of SG assembly and NLRP3 inflammasome. First, we determined whether FAM69C was a component of inflammasome. Immunofluorescence showed that FAM69C was not colocalized with NLRP3 (Fig EV3D). As previously reported that sequestration of

 © 2023 The Authors

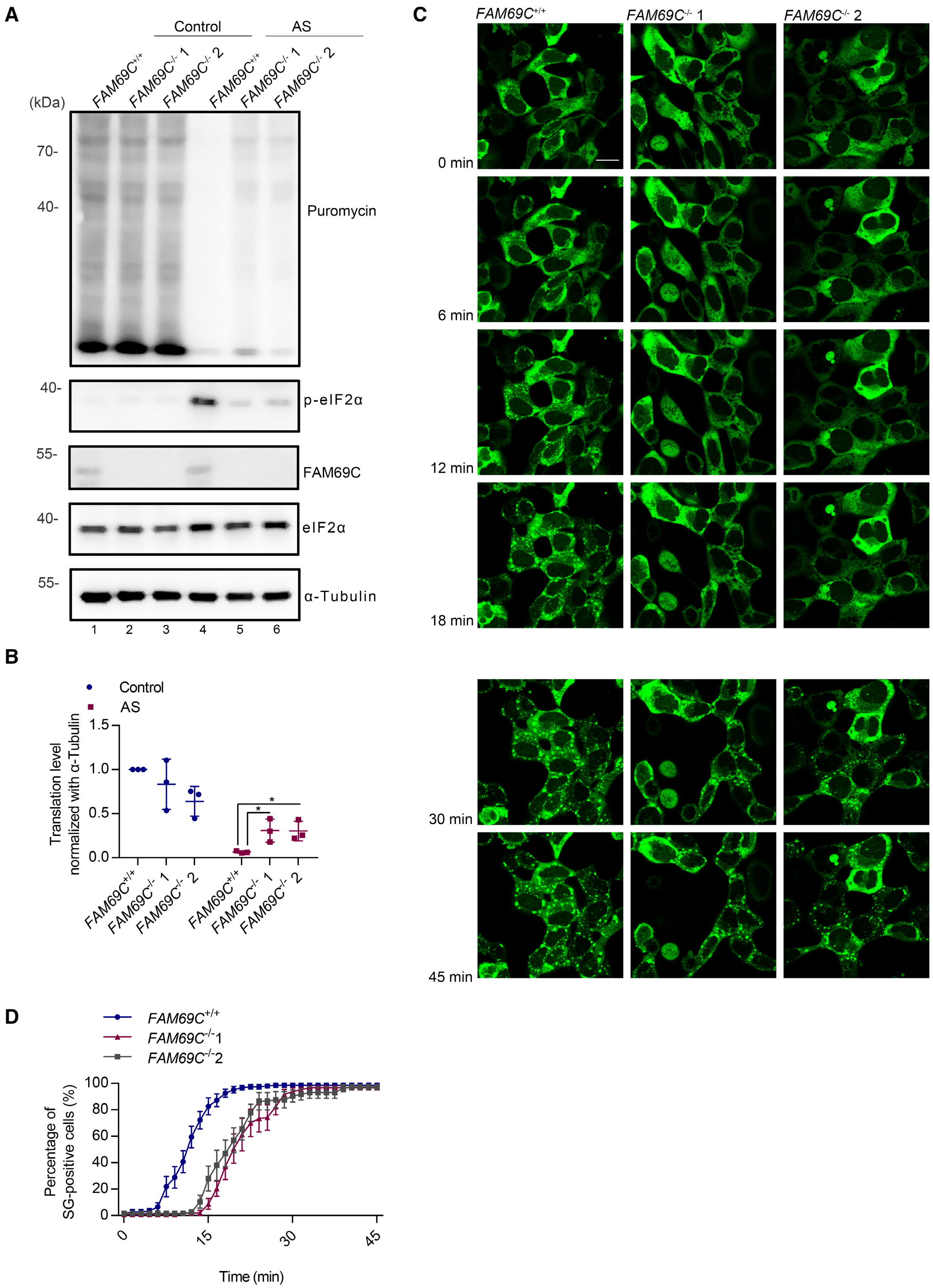
A
Control
AS
FAM69C+/+
FAM69C-/- 1
FAM69C-/- 2
(kDa)
70-
40-
Puromycin
p-eIF2α
55-
FAM69C
eIF2α
α-Tubulin
1 2 3 4 5 6
B
Control
AS
Translation level normalized with α-Tubulin
1.5
1.0
0.5
0.0
C
0 min
6 min
12 min
18 min
30 min
45 min
D
FAM69C-/-1
FAM69C-/-2
Percentage of SG-positive cells (%)
100
80
60
40
20
0
15
30
45
Time (min)

Figure 2.

Figure 2. FAM69C mediates stress-induced translation arrest and promotes stress granule assembly.

A Immunoblot analysis of *de novo* synthesized proteins in SH-SY5Y cells. Puromycin incorporation assay of *FAM69C*$^{+/+}$ and 2 *FAM69C*$^{-/-}$ SH-SY5Y cell lines treated with 0.5 mM AS for 30 min followed by 50 µg/ml puromycin for 15 min. An anti-puromycin antibody (Puro) was used to visualize *de novo* synthesized proteins. A representative image is shown, $n = 3$ biologically independent experiments.

B Quantitative analysis of puromycin labeled *de novo* synthesized proteins. *P value < 0.05, paired two-tailed t-test, $n = 3$ biologically independent experiments. Mean ± SD. For *FAM69C*$^{+/+}$ versus *FAM69C*$^{-/-}$ 1, P value = 0.0416; For *FAM69C*$^{+/+}$ versus *FAM69C*$^{-/-}$ 2, P value = 0.0346.

C Live-cell imaging showing the delayed stress granule assembly in *FAM69C*$^{-/-}$ SH-SY5Y cells. Time-lapse images of *FAM69C*$^{+/+}$ and *FAM69C*$^{-/-}$ SH-SY5Y cells stably expressing GFP-tagged G3BP1 were acquired after cells were exposed to 0.1 mM AS for 45 min. Representative images, $n > 3$ biologically independent experiments. Scale bar = 10 µm.

D Quantification of cells with stress granules at different times. For *FAM69C*$^{+/+}$, $n = 8$ experiments; *FAM69C*$^{-/-}$ 1, $n = 8$ experiments; *FAM69C*$^{-/-}$ 2, $n = 9$ experiments. Mean ± SEM.

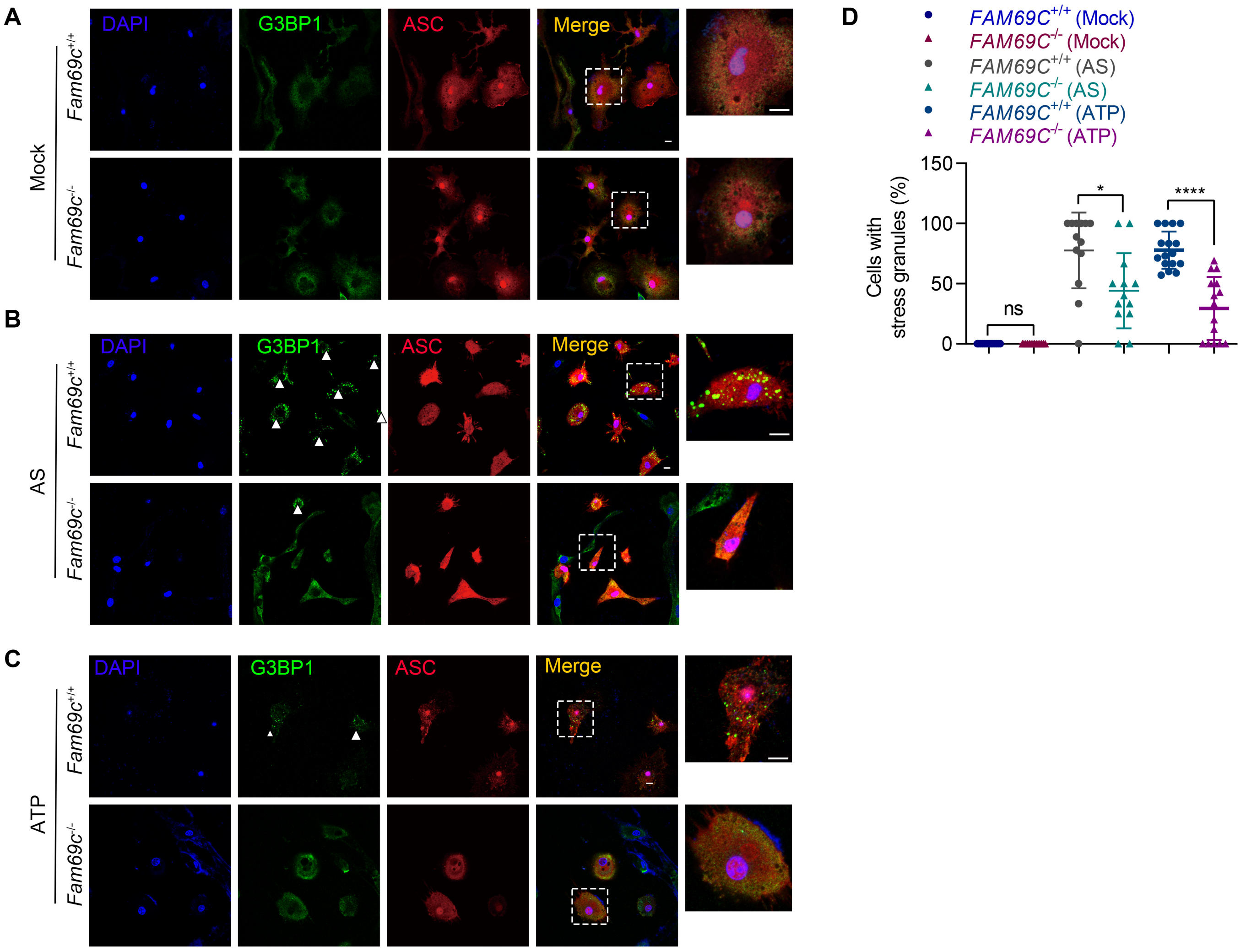

Figure 3. FAM69C facilitates stress granule assembly in microglia.

A–C Confocal imaging of primary cultured *Fam69c*$^{+/+}$ and *Fam69c*$^{-/-}$ microglia treated with mock, 0.5 mM AS (20 min), and 5 mM ATP (40 min) to visualize the formation of stress granules. *Fam69c*$^{-/-}$ microglia showed impaired stress granule (indicated by arrowheads) assembly compared with *Fam69c*$^{+/+}$ microglia when treated with AS and ATP. Representative images, $n = 3$ biologically independent experiments. Scale bar = 10 µm.

D Quantitative analysis of microglia with stress granules. For AS, *P value = 0.0118, unpaired two-tailed t-test, $n = 13$ fields from three biologically independent experiments. For ATP, ****P value < 0.0001, unpaired two-tailed t-test, $n = 15$ fields from three biologically independent experiments. Mean ± SD.

DDX3X in stress granule prevents its interaction with NLRP3 and inflammasome activation (Samir *et al*, 2019), we determined the status of DDX3X sequestration in *Fam69c*$^{+/+}$ and *Fam69c*$^{-/-}$ microglia. As expected, ATP exposure induces SG assembly and DDX3X sequestration in SG in *Fam69c*$^{+/+}$ microglia, as compared with control (Fig 4D). By contrast, loss of *FAM69C* failed to sequester DDX3X.

 © 2023 The Authors

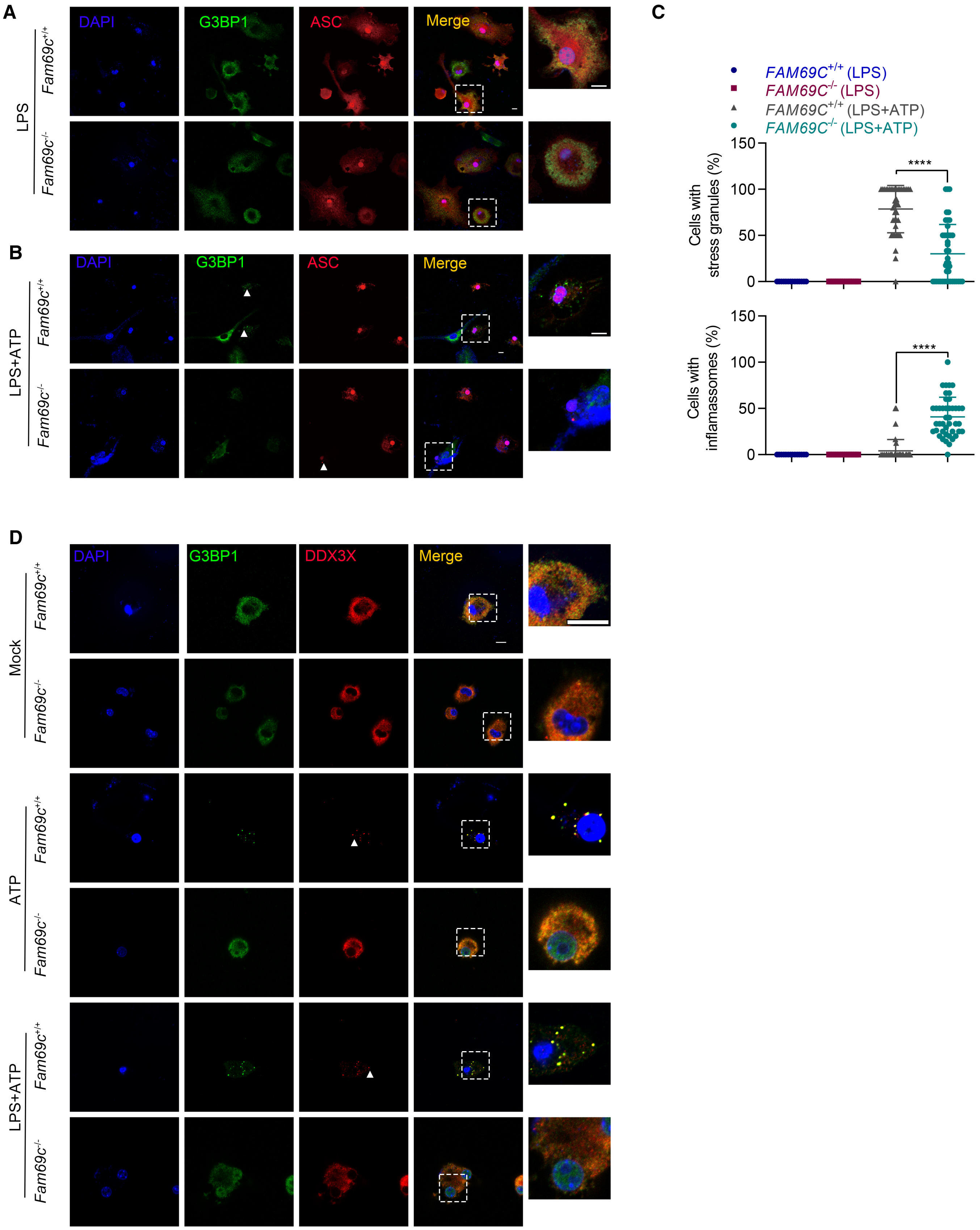
A
LPS
Fam69c+/+
Fam69c-/-
DAPI
G3BP1
ASC
Merge
B
LPS+ATP
C
FAM69C+/+ (LPS)
FAM69C-/- (LPS)
FAM69C+/+ (LPS+ATP)
FAM69C-/- (LPS+ATP)
Cells with stress granules (%)
Cells with inflamassomes (%)
150
100
50
0
****
D
Mock
ATP
DDX3X

Figure 4.

Figure 4. Stress granule assembly in microglia is correlated with reduced NLRP3 inflammasome activation.

A, B Confocal imaging of LPS (1 μg/ml, 3 h) primed primary cultured *Fam69c*$^{+/+}$ and *Fam69c*$^{-/-}$ microglia treated with or without 5 mM ATP for 40 min to visualize the formation of stress granules (green, indicated by arrowheads) and inflammasomes (red, indicated by arrowheads). LPS-primed *Fam69c*$^{-/-}$ microglia showed impaired ATP-induced stress granule assembly compared with *Fam69c*$^{+/+}$ microglia when treated with ATP. Meanwhile, more cells with ASC specks were found in *Fam69c*$^{-/-}$ microglia than that in *Fam69c*$^{+/+}$ microglia. Representative images, $n > 3$ biologically independent experiments. Scale bar = 10 μm.

C Quantitative analysis of microglia with stress granules and inflammasomes treated with LPS, LPS and ATP. ****P value < 0.0001, unpaired two-tailed t-test. For LPS, $n = 15$ fields from three biologically independent experiments; For LPS + ATP, $n = 40$ fields from six biologically independent experiments. Mean ± SD.

D Confocal imaging of primary cultured *Fam69c*$^{+/+}$ and *Fam69c*$^{-/-}$ microglia treated with mock, 5 mM ATP (40 min), 1 μg/ml LPS (3 h) and 5 mM ATP (40 min) to visualize DDX3X (red, indicated by arrowhead) localization. G3BP1 was used as a marker of stress granules. *Fam69c*$^{+/+}$ microglia form stress granules and lead to the sequestration of DDX3X when treated with ATP. Scale bar = 10 μm.

Consistently, LPS-primed *Fam69c*$^{+/+}$ microglia forms SGs with complete sequestration of DDX3X, whereas *Fam69c*$^{-/-}$ microglia showed no signs of such sequestration (Fig 4D). Thus, we speculate that FAM69C facilitates SG assembly, which sequesters DDX3X to prevent NLRP3 inflammasome activation.

FAM69C-independent inflammasome activation

It has been well characterized that NLRP3 inflammasome is induced by various inducers, through potassium-dependent and potassium-independent mechanisms (Mangan *et al*, 2018). Based on the findings that FAM69C regulates ATP-induced SGs in microglia and correlated with reduced inflammasome activation, we examined the role of FAM69C in other induction conditions. Nigericin is one of the most commonly used inflammasome inducers, which permeabilizes the cell membrane specifically to potassium. Without priming, *Fam69c*$^{+/+}$ and *Fam69c*$^{-/-}$ microglia did not show inflammasome activation upon nigericin treatment (Fig 5A). After LPS priming, nigericin induced a comparable number of ASC specks in *Fam69c*$^{+/+}$ and *Fam69c*$^{-/-}$ microglia, in the absence of SG formation (Fig 5B and C). To corroborate these findings, we employed the lysosomotropic reagent L-leucyl-L-leucine methyl ester (Leu-Leu-OMe) to disrupt lysosomes. Likewise, Leu-Leu-OMe did not induce SGs, but activated comparable number of NLRP3 inflammasome independent of *FAM69C* genotype both in the primed and unprimed microglia (Fig 5D–F). Taken together, we proposed that FAM69C-associated inflammasome inhibition in microglia is dependent on conditions that induce SG assembly. To this end, ATP exposure, but not other inflammasome inducers, is related to the crosstalk between FAM69C-dependent SG assembly and inflammasome activation in microglia.

SG assembly precludes NLRP3 inflammasome activation

To examine whether the correlation between SG assembly and inflammasome inhibition is a general observation, we induced SG assembly in microglia and followed by treatment with the inflammasome inducer. To this end, microglia were primed with LPS, pretreated with AS to induce SG assembly, and followed by nigericin treatment (Fig 6A). Remarkably, SG assembly did preclude NLRP3 inflammasome activation (Fig 6B). Sorbitol, like AS, was able to induce SG assembly in primed microglia (Fig EV3E). Consistently, we found that the presence of sorbitol-induced SGs inhibited nigericin-induced inflammasome formation (Fig 6C and D).

Next, we tried to rescue exacerbated inflammasome activation in *Fam69c*$^{-/-}$ microglia. We prolonged the AS treatment from 20 to 40 min and followed by ATP treatment. Prolonged AS treatment was sufficient for the finalization of delayed SG assembly in *Fam69c*$^{-/-}$ microglia (Fig 6E). With the presence of SGs, inflammasome activation represented by ASC specks was completely suppressed in *Fam69c*$^{-/-}$ microglia (Fig 6F). Taken together, we proposed that SG assembly precludes NLRP3 inflammasome formation.

Aged FAM69C-deficient mice show inflammasome activation

As the predominant type of innate immune cells in the brain, microglia are the main source of proinflammatory cytokines. Cytokines released by microglia are key mediators of neuroinflammation, which can induce and regulate a wide range of cellular responses (Colonna & Butovsky, 2017). The homeostasis of microglia is thus critical for normal brain function. Moreover, proinflammatory cytokines lead to dysfunctional microglia and dampen the clearance of misfolded proteins in neurodegenerative disease.

Based on these findings in primary microglia derived from *Fam69c*$^{+/+}$ and *Fam69c*$^{-/-}$ mice, we determined the effect of FAM69C in microglia-mediated inflammation *in vivo*. Given that aging parallels the induction of inflammation, we classified mice into 3-month-old young group and 12-month-old aged group. Cortical tissues from the aged *Fam69c*$^{-/-}$ mice showed significantly increased NLRP3 levels and caspase-1 cleavage, indicating inflammasome activation (Fig 7A and B). Moreover, we examined caspase-1-mediated proteolytic activation of cytokines. qRT-PCR demonstrated that the expression of *Il18* and its receptor *Il18r1* were significantly upregulated in the aged *Fam69c*$^{-/-}$ mice (Fig 7C). Further, immunofluorescence revealed an increased number of ASC-expression microglia in the medial prefrontal cortex (mPFC) of aged *Fam69c*$^{-/-}$ mice than that from *Fam69c*$^{+/+}$ mice (Fig 7D and E). Consistently, we observed a greater number of eosinophilic neurons in the cortex of aged *Fam69c*$^{-/-}$ mice, as compared with the *Fam69c*$^{+/+}$ mice (Fig 7F). By contrast, no inflammasome activation was observed in the 3-month-old young *Fam69c*$^{-/-}$ mice (Fig EV4A). HE staining showed no evident difference between *Fam69c*$^{-/-}$ mice and age-matched young *Fam69c*$^{+/+}$ mice (Fig EV4B). These data indicated that FAM69C deficiency together with aging parallels microglia-associated inflammasome activation.

Taken together, these data suggested that aged FAM69C-deficient mice show inflammasome activation.

Discussion

In our previous study, we identified that FAM69C is a brain-enriched kinase linked to neurodegenerative diseases (Mei

 © 2023 The Authors

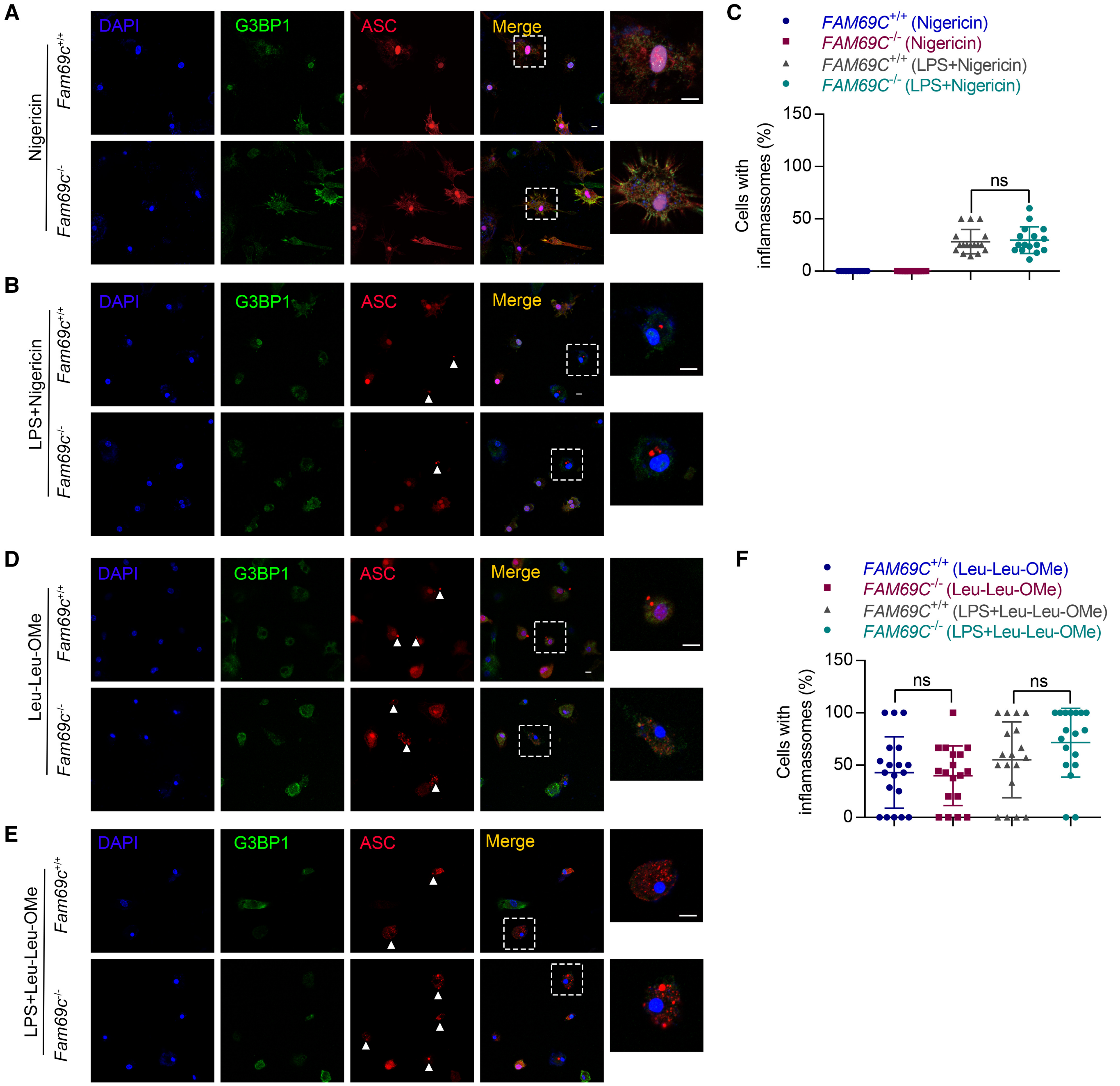

Figure 5. FAM69C-independent inflammasome activation.

A, B Confocal imaging of primary cultured *Fam69c*$^{+/+}$ and *Fam69c*$^{-/-}$ microglia treated with 20 μM nigericin (40 min), 1 μg/ml LPS (3 h) and 20 μM nigericin (40 min), to visualize the formation of stress granules and inflammasomes. Microglia form inflammasomes (indicated by arrowheads) and did not form stress granules when treated with nigericin. Representative images, $n = 3$ biologically independent experiments. Scale bar = 10 μm.

C Quantification of cells with inflammasomes. Primary cultured *Fam69c*$^{+/+}$ and *Fam69c*$^{-/-}$ microglia were treated with 20 μM nigericin (40 min), 1 μg/ml LPS (3 h) and 20 μM nigericin (40 min). ns = no significant, unpaired two-tailed *t*-test, $n = 16$ fields from three biologically independent experiments. Mean ± SD.

D, E Confocal imaging of primary cultured *Fam69c*$^{+/+}$ and *Fam69c*$^{-/-}$ microglia treated with 1 mM Leu-Leu-Ome (3 h), 1 μg/ml LPS (3 h) and 1 mM Leu-Leu-Ome (3 h), to visualize the formation of stress granules and inflammasomes. Microglia form inflammasomes (indicated by arrowheads) and did not form stress granules when treated with Leu-Leu-Ome. Representative images, $n = 3$ biologically independent experiments. Scale bar = 10 μm.

F Quantification of cells with stress granules and inflammasomes. Primary cultured *Fam69c*$^{+/+}$ and *Fam69c*$^{-/-}$ microglia were treated with 1 mM Leu-Leu-Ome (3 h), 1 μg/ml LPS (3 h) and 1 mM Leu-Leu-Ome (3 h). ns = no significant, unpaired two-tailed *t*-test, $n = 18$ fields from three biologically independent experiments. Mean ± SD.

© 2023 The Authors

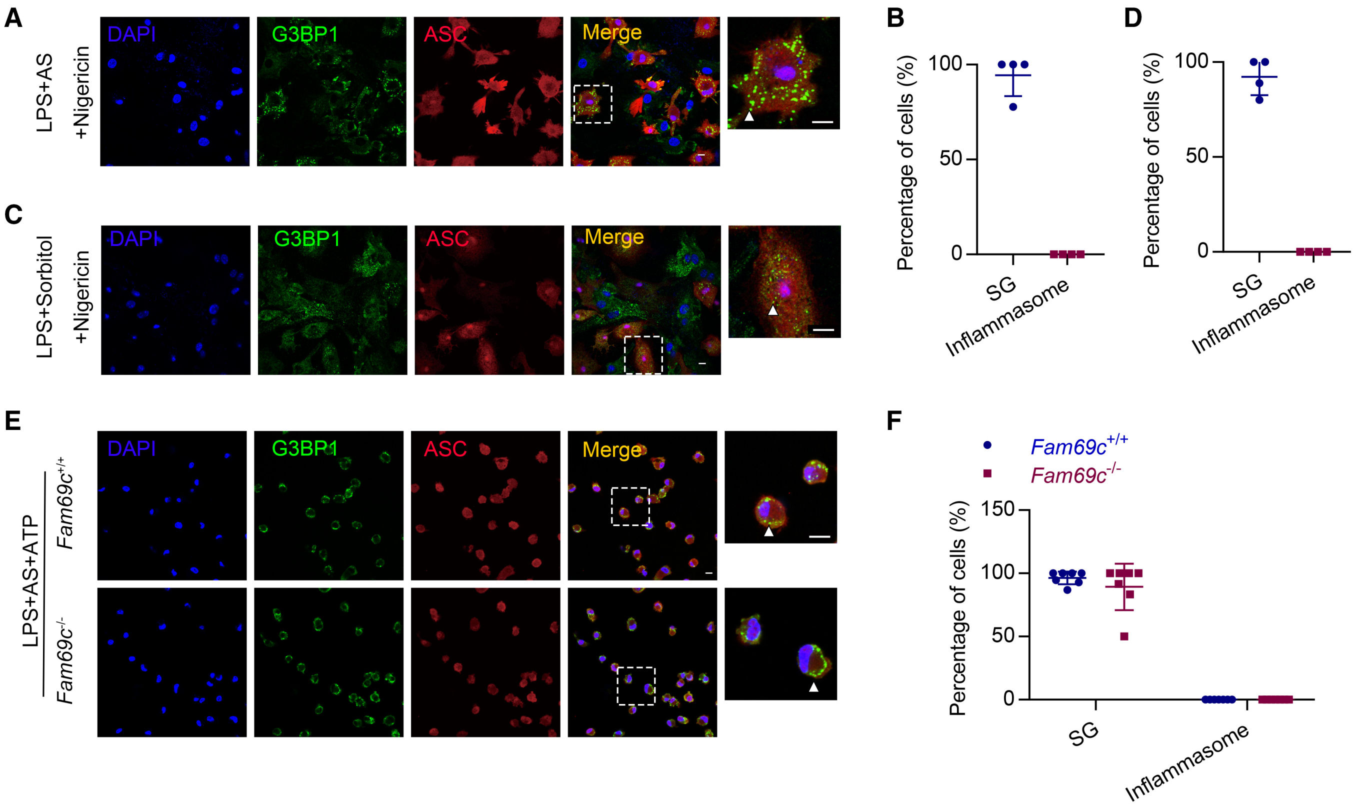

Figure 6. SG assembly precludes NLRP3 inflammasome activation.

A–D AS- and Sorbitol-induced stress granules suppressed the formation of nigericin-induced inflammasomes in microglia. (A, C) Confocal imaging of primary cultured microglia treated with 1 μg/ml LPS (3 h), followed by 0.5 mM AS (40 min) or 0.4 M sorbitol (1 h) and 20 μM nigericin (40 min), to visualize the formation of stress granules (indicated by arrowheads) and inflammasomes. Representative images, $n = 3$ biologically independent experiments. Scale bar = 10 μm. (B, D) Quantification of cells with stress granules and inflammasomes. Primary cultured microglia were treated with LPS + AS + Nigericin (B) and LPS + Sorbitol + Nigericin (D), $n = 4$ fields from two biologically independent experiments. Mean ± SD.

E Confocal imaging of primary cultured *Fam69c*$^{+/+}$ and *Fam69c*$^{-/-}$ microglia treated with 1 μg/ml LPS (3 h), followed by 0.5 mM AS (40 min) and 5 mM ATP (40 min), to visualize the formation of stress granules and inflammasomes. AS-induced stress granules (indicated by arrowheads) suppressed the formation of ATP-induced inflammasomes in *Fam69c*$^{-/-}$ microglia. Representative images, $n = 2$ biologically independent experiments. Scale bar = 10 μm.

F Quantification of cells with stress granules and inflammasomes. Primary cultured *Fam69c*$^{+/+}$ and *Fam69c*$^{-/-}$ microglia were treated with LPS + AS+ATP. ns = no significant, unpaired two-tailed *t*-test, $n = 7$ fields from two biologically independent experiments. Mean ± SD.

et al, 2022). The substrates of FAM69C and its biological function are largely unknown. Here, we found that FAM69C functions as an eIF2α kinase and promotes stress granule assembly. Specifically, we characterized the impact of FAM69C-dependent stress granule assembly on microglia function. This study suggests the potential protective role of FAM69C in aging and neurodegenerative diseases.

The cellular stress response has a vital role in the regulation of brain homeostasis (Farley & Watkins, 2018). eIF2α is a critical molecule in stress responses, related to integrated stress response and stress granule assembly. Phosphorylation of eIF2α suppresses general protein translation and subsequently promotes SG assembly, which is composed of condensates of RNA-binding proteins and untranslated messenger RNAs. Here, we found that ATP is able to induce SG assembly in microglia. We provided evidence that ATP treatment leads to increased levels of phosphorylated eIF2α. By contrast, the inflammasome inducer nigericin was not able to induce phosphorylation of eIF2α (Fig EV5A and B). Previous studies have employed both ATP and nigericin as inflammasome inducers in macrophages (Iyer *et al*, 2009; Samir *et al*, 2019). Here, we showed the difference between ATP and nigericin in the activation of eIF2α signaling and induction of SG assembly in microglia. Further, we provided evidence that FAM69C functions as a novel stress-specific kinase for eIF2α, in addition to the four known eIF2α kinases. We delineate the role of FAM69C in the regulation of SG assembly through eIF2α signaling.

Previous studies have shown that SG assembly and inflammasome activation determine contrasting live-or-die fates of stressed macrophages (Samir *et al*, 2019). As for the molecular mechanisms underlying the interplay between SG assembly and inflammasome activation, two molecules have been well studied. HSP 90 and DDX3X are conserved components of SGs, but also play a direct role in NLRP3 inflammasome activation. Specifically, sequestration of HSP90 and DDX3X in SGs inhibits the formation of NLRP3 inflammasomes (Mayor *et al*, 2007; Samir *et al*, 2019). In our study, FAM69C facilitates ATP-induced stress granule assembly in

© 2023 The Authors

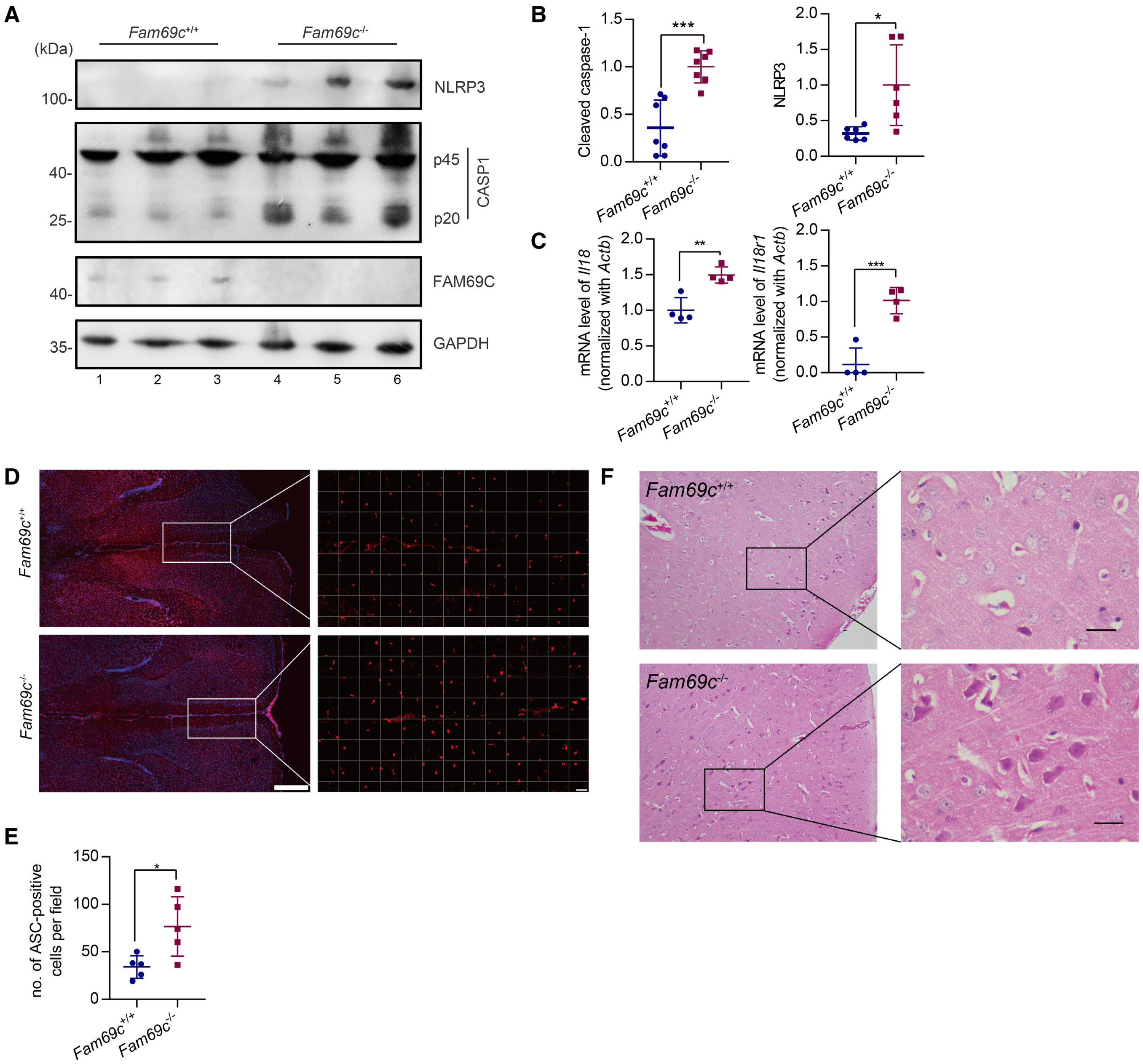

Figure 7. Aged FAM69C-deficient mice show inflammasome activation.

- A Immunoblot analysis of NLRP3 and cleaved caspase1 (p20) in cortex of 12-month-old *Fam69c*$^{+/+}$ and *Fam69c*$^{-/-}$ mice. Data are from three different mice with each genotype.
- B Quantification of NLRP3 and cleaved caspase1 (p20) normalized with GAPDH in the cortex of 12-month-old *Fam69c*$^{+/+}$ and *Fam69c*$^{-/-}$ mice. For caspase1 (p20), ***P value = 0.0003, n = 7 mice/genotype; For NLRP3, *P value = 0.0159, n = 6 mice/genotype. Unpaired two-tailed t-test, Mean ± SD.
- C Quantification of *Il18* and *Il18r1* expression normalized with *Actb* in the cortex of 12-month-old *Fam69c*$^{+/+}$ and *Fam69c*$^{-/-}$ mice. ***P value = 0.0009; **P value = 0.0035, unpaired two-tailed t-test, n = 4 mice/group. Mean ± SD.
- D Representative image of ASC staining of *Fam69c*$^{+/+}$ and *Fam69c*$^{-/-}$ mouse mPFC. 12-month-old *Fam69c*$^{+/+}$ and *Fam69c*$^{-/-}$ mice were sacrificed for Frozen sections and ASC immunofluorescence. The density of ASC-positive cells in the mPFC of *Fam69c*$^{-/-}$ mice was higher than *Fam69c*$^{-/-}$ mice. Scale bars, 500 μm (left), 50 μm (right). Representative images (n = 5).
- E Quantitative analysis of ASC-positive cells in mPFC of *Fam69c*$^{+/+}$ and *Fam69c*$^{-/-}$ mouse with Image J software. *P value = 0.0215, unpaired two-tailed t-test, n = 5 mice/ genotype. Mean ± SD.
- F Hematoxylin and eosin staining of 10-month-old *Fam69c*$^{+/+}$ and *Fam69c*$^{-/-}$ mouse brain. *Fam69c*$^{-/-}$ mice showed more eosinophilic neurons in the cortex than *Fam69c*$^{-/-}$ mice. The image is representative of three pairs of mice. Scale bar = 25 μm.

© 2023 The Authors

microglia, which leads to the sequestration of DDX3X in SG. Meanwhile, we observed decreased inflammasome activation in *Fam69c*$^{+/+}$ microglia as compared with *Fam69c*$^{-/-}$ microglia. These data suggest that FAM69C may indirectly regulate inflammasome activation through DDX3X. Further, we provided evidence that induction of SG assembly in *Fam69c*$^{-/-}$ microglia rescued exacerbated inflammasome activation. Our work illustrates that FAM69C promotes SG assembly in microglia and regulates microglia function, albeit its impact on reduced inflammasome activation is not direct.

Activation of microglia contributes to neuroinflammation (Hammond *et al*, 2019). Pathological misfolded protein aggregates and DAMPs released from dead neurons can activate microglia through inflammasomes. Innate immunity contributes to the development of neurodegenerative diseases. Inflammasome activation in *Fam69c*$^{-/-}$ mice was manifested by increased NLRP3 expression, caspase-1 cleavage, and *Il18* expression. Changes of another key interleukin IL-1β involved in inflammasome activation were not seen in qRT-PCR, which may be due to the relatively lower expression of *Il1b* than *Il18*. In the future study, we may isolate microglia, rather than the whole tissue lysates from the aged *Fam69c*$^{-/-}$ brain, and measure the expression of *Il1b* .

In conclusion, we discovered that the brain-enriched kinase FAM69C promotes SG assembly through the phosphorylation of eIF2a. Stress-induced stress granule assembly in microglia precludes NLRP3 inflammasome activation. These findings reveal a new molecular mechanism for the initiation of stress granules and demonstrate the role of FAM69C in the regulation of microglia function.

Materials and methods

Animals and cell lines

All animals were maintained in a special pathogen-free facility, and the animal study protocols used were approved by the ethics committee of Peking University Health Science Center (approval number LA2017142). The 293T, Hela, and SH-SY5Y cell lines were obtained from the American Type Culture Collection. The BV2 microglial cell line was purchased from Procell Co., Ltd. and has been authenticated recently (#CL-0493, Wuhan, China).

Fam69c$^{-/-}$ mice and *FAM69C*$^{-/-}$ cell line

Fam69c$^{-/-}$ mice and *FAM69C*$^{-/-}$ SH-SY5Y cell line used in this study were generated using the CRISPR design tool, which has been described previously by Mei *et al* (2022).

Antibodies

The following antibodies were used in this study: ASC (#67824, CST), α-tubulin (#RM2007, Ray antibody Biotech), β-actin (#PM053, MBL), Caspase-1 (p20) (AG-20B-0042, AdipoGen), DDX3X (ab271002, Abcam), eIF2α (#5324, CST), Phospho-eIF2α (Ser51) (#3398, CST), FAM69C (home-made), FLAG (#F3165, Sigma-Aldrich), G3BP1 (sc-365338, Santacruz), GAPDH (#RM2002, Ray antibody Biotech), GFAP (#12389, CST), GFP (#RM1008, Ray antibody Biotech), GM130 (#2296, CST), HA (#H3663, Sigma-Aldrich), HRI (sc-365239, Santacruz), Iba1 (019–19741, Wako), PERK (#5683, CST), Puromycin (MABE341, Millipore).

Co-immunoprecipitation

Cells were lysed with Co-IP lysis buffer (150 mM NaCl, 1 mM EDTA, 20 mM Tris–HCl pH 8.0, 0.5% NP40, and 0.1 mM PMSF). Then, the cell lysates were incubated with S-protein agarose (Novagen) for 90 min. The beads were washed with 0.3% NP40 three times and eluted with SDS-PAGE loading buffer (50 mM Tris–HCl pH 6.8, 2% SDS, 0.1% bromophenol blue, 10% glycerol, and 5% 2-mercaptoethanol) by boiling for 10 min, followed by immunoblot analysis.

Establishment of stable knockdown cell lines

To generate stable HRI and PERK-silenced cells, shRNA against HRI and PERK were cloned into pLKO.1 Vector. HEK293T cells were cotransfected with pLKO.1 recombinant construct and the packaging plasmids pMD2.G and psPAX2 for 48 h. Media were collected and filtered with a 0.22 μm filter. The cell culture supernatants containing lentivirus were used to infect *FAM69C*$^{+/+}$ and *FAM69C*$^{-/-}$ SH-SY5Y cell line. After infection for 48 h, the stable silenced cells were screened with 2 μg/ml puromycin. The target sequences by shRNAi are listed below:

shHRI-1, AGCTACTTTGCCAGACGTTTA;
shHRI-2, GGATTGGATAGTCGAGAGAAA;
shPERK-1, CCGTAGTAAGAAATGGATCAT;
shPERK-2, GCACACAGATTACAGTCAGAT.

HE staining and ASC staining

Mice were sacrificed for HE staining and ASC staining. The paraffin-embedded brain was sectioned into 5 μm slices, followed by HE staining. For ASC staining, frozen sections were used. The sections were blocked in 5% BSA, 0.3% Triton X-100 and PBS (blocking buffer) for 30 min followed by 3 h of room temperature incubation with the ASC antibody (#67824, CST) in the blocking buffer. Sections were washed three times in PBS and incubated with Alexa Fluor 555 conjugated secondary antibodies for 60 min, followed by DAPI for 10 min. To quench autofluorescence, the sections were incubated for 20 min in 0.1% Sudan Black B in 75% ethanol and washed extensively in PBS.

Immunoblot

For immunoblot, cultured cells were lysed in 0.5% NP40 lysis buffer, and mouse tissues were lysed in RIPA buffer (50 mM Tris–HCl [pH 7.4], 150 mM NaCl, 2 mM EDTA, 1% NP-40, 0.5% sodium deoxycholate, 0.1% SDS, 100 mM PMSF). For phosphorylated protein detection, PIC inhibitor (Roche) and phosphatase inhibitor (10 mM NaF, 1 mM Na_3VO_4) were added additionally.

Proteins were separated by SDS-PAGE and blotted on PVDF membranes. The membranes were blocked with 5% nonfat milk in PBS-T (1% Tween 20)/TBS-T (20 mM Tris, Ph8.0, 150 mM NaCl with 0.5% Tween 20) for 1 h at RT and incubated with the indicated antibodies overnight at 4°C. After washing with PBS-T/TBS-T buffer three times, membranes were incubated with HRP-conjugated secondary antibodies for 1 h at RT.

© 2023 The Authors

Immunofluorescence

Cells seeded on glass coverslips were fixed with 4% PFA at room temperature for 15 min, followed by blocking in 1% bovine serum albumin containing 0.3% tritonX-100 at room temperature for 1 h, and incubation in the diluted primary antibody at 4°C overnight. After being incubated with secondary antibodies at room temperature for 30 min and stained with 0.5 μg/ml DAPI for 10 min, coverslips were mounted with fluorescence decay resistant medium. Images were acquired with a Nikon TCS A1 microscope.

In vitro phosphorylation assay

10 μg recombinant eIF2α protein was mixed with or without 1 μg FAM69C protein in reaction buffer (40 mM Tris–HCl (pH7.5), 20 mM MgCl2, 1 mM ATP) at 37° for 30 min.

FAM69C was obtained as described by Mei (Mei *et al*, 2022). For recombinant expression of eIF2α, DNA fragments encoding human eIF2α were cloned into a pET-28a (+) vector with His tag on the N terminal. BL21 cells transfected with eIF2α were treated with 0.1 mM IPTG for 20 h and then lysed by ultrasonic. eIF2α was enriched with Ni Beads.

Live-cell imaging

Cells stably expressing GFP-tagged G3BP1 were established for live-cell imaging. *FAM69C*$^{+/+}$ or *FAM69C*$^{-/-}$ cells were seeded on cell culture dishes (Bioptechs Inc) 24 h before imaging. The heater was used to keep the temperature at 37°C. Multipoint images were taken every 90 s with the 488 nm laser after 0.1 mM AS treatment immediately.

Membrane and cytoplasm isolation

HEK 293T Cells expressing C-terminal GFP tagged FAM69C were grown to confluency in a 150 mm dish and treated with or without 0.5 mM sodium arsenite (AS) for 1 h. Membrane and cytoplasm were isolated with Applygene P1201. For density gradient centrifugation, cells were lysed in CER buffer (Applygene P1201) for 15 s. The lysate was then centrifuged for 5 min at 800 *g*. 1 ml Supernatant was layered on a linear sucrose gradient (15–45%, 400 μl/layer, 11 layer), centrifuged at 100,040 *g* in a Beckman sw55Ti rotor at 4°C for 40 min. Fractions 1–10 (500 μl each) were harvested from the top to the bottom. 15 μl from each fraction was analyzed by western blot. Fraction 1 represents the lowest density fraction, and fraction 10 represents the highest density.

Primary microglia culture

Newborn mice were sacrificed for mixed glial culture, and primary glial cells were further cultured in DMEM supplemented with 10% FCS, 100 U/ml penicillin/streptomycin, and 20 ng/ml GM-CSF. Microglial cells were used after 14 days of primary cultivation. To assess the formation of ASC specks, LPS-primed microglia were activated with nigericin (20 μM) or ATP (5 mM) for 40 min. Cells were then fixed for further immunofluorescence assays.

qRT-PCR

mRNA from mice brains was reverse transcribed into cDNA with All-In-One RT MasterMix (Abm), and quantitative real-time polymerase chain reaction (qPCR) was carried out on a 7,500 Fast real-time PCR system.

The following primers for the *interleukin-18 (Il18)*, *interleukin-18 receptor 1 (Il18r1)*, *interleukin-1 beta (Il1b)*, *interleukin-1 receptor type 1 (Il1r1)*, and *Beta-actin (Actb)* genes were used:

Il18: sense 5′-GACTCTTGCGTCAACTTCAAGG-3′, antisense 5′- CAG GCTGTCTTTTGTCAACGA-3′;
Il18r1: sense 5′-ACTTTTGCTGTGGAGACGTTAC-3′, antisense 5′- CC GGCTTTTCTCTATCAGTGAAT-3′;
Il1b: sense 5′-GAAATGCCACCTTTTGACAGTG-3′, antisense 5′-TGG ATGCTCTCATCAGGACAG-3′;
Il1r1: sense 5′- GGGAAGCAATATCCGGTCACA-3′, antisense 5′-TGA CGTTGCAGATCAGTTGTATC-3′;
Actb: sense 5′-GGCTGTATTCCCCTCCATCG-3′, antisense 5′-CCA GTTGGTAACAATGCCATGT-3′.

RiboPuromycylation

FAM69C$^{+/+}$ and *FAM69C*$^{-/-}$ cells were treated with or without 0.5 mM AS for 30 min, followed by puromycin (50 μg/ml) treatment for 15 min. Puromycin-labeled proteins were identified with immunoblot.

S-tag pull-down assay

SH-SY5Y cells stably expressing S-HA-tagged FAM69C were grown to confluency in a 150 mm dish for S-tag pull-down. Cells were lysed for 30 min in 0.5% NP40 lysis buffer, and FAM69C was recognized and pulled down by the S-protein Agarose beads. The FAM69C-bounded S-protein Agarose beads were washed 3 times with 0.3% NP40 buffer, followed by a mass spectrometry assay. ClueGO-v2.5.8 (Bindea *et al*, 2009) was used for GO enrichment analysis of potential interacting proteins.

Transfections

Transient transfections were performed with polyethylenimine (PEI) and Opti-MEM. For stable transfection construction, psPAX2, pMD2.G, and pCDH-CMV-MCS-EF1-puro-G3BP1-GFP were cotransfected into HEK293T to produce lentivirus. Cell culture supernatants containing lentivirus were collected, filtered, and then added into *FAM69C*$^{+/+}$ and *FAM69C*$^{-/-}$ SH-SY5Y cell line. After infection for 48 h, the stable cell lines were screened by puromycin.

Data availability

The data set (and computer code) produced in this study is available in the following database: Mass spectrometry data: Proteomics Identification Database PXD037697 (https://www.ebi.ac.uk/pride/archive/projects/PXD037697). Any additional information required to reanalyze the data reported in this paper is available from the lead contact upon request.

© 2023 The Authors

Expanded View for this article is available online.

Acknowledgements

We thank Dr. X. Zhao and L. Liang from Peking University Health Science Center for mass spectrometry and protein purification. This study was supported by grants including the National Key Research and Development Program of China (2021YFA1300601); National Natural Science Foundation of China (grant 82030081, 81874235, 82071207); the Lam Chung Nin Foundation for Systems Biomedicine; and Beijing Municipal Natural Science Foundation (7232099).

Author contributions

Zhongyan Wu: Conceptualization; data curation; software; formal analysis; validation; investigation; visualization; methodology; writing – original draft. **Fan Mei:** Conceptualization; data curation; funding acquisition; writing – original draft. **Yangyang Gan:** Resources; writing – review and editing. **Anhang Liu:** Software. **Jiapan Hu:** Resources; writing – review and editing. **Yan Jin:** Supervision; project administration. **Yuxin Yin:** Conceptualization; funding acquisition; project administration; writing – review and editing.

Disclosure and competing interests statement

The authors declare that they have no conflict of interest.

References

Aulas A, Fay MM, Lyons SM, Achorn CA, Kedersha N, Anderson P, Ivanov P (2017) Stress-specific differences in assembly and composition of stress granules and related foci. *J Cell Sci* 130: 927 – 937

Baron DM, Kaushansky LJ, Ward CL, Sama RR, Chian RJ, Boggio KJ, Quaresma AJ, Nickerson JA, Bosco DA (2013) Amyotrophic lateral sclerosis-linked FUS/TLS alters stress granule assembly and dynamics. *Mol Neurodegener* 8: 30

Bindea G, Mlecnik B, Hackl H, Charoentong P, Tosolini M, Kirilovsky A, Fridman WH, Pagès F, Trajanoski Z, Galon J (2009) ClueGO: a Cytoscape plug-in to decipher functionally grouped gene ontology and pathway annotation networks. *Bioinformatics* 25: 1091 – 1093

Boeynaems S, Bogaert E, Kovacs D, Konijnenberg A, Timmerman E, Volkov A, Guharoy M, De Decker M, Jaspers T, Ryan VH *et al* (2017) Phase separation of C9orf72 dipeptide repeats perturbs stress granule dynamics. *Mol Cell* 65: 1044 – 1055.e5

Bosco DA, Lemay N, Ko HK, Zhou H, Burke C, Kwiatkowski TJ Jr, Sapp P, McKenna-Yasek D, Brown RH Jr, Hayward LJ (2010) Mutant FUS proteins that cause amyotrophic lateral sclerosis incorporate into stress granules. *Hum Mol Genet* 19: 4160 – 4175

Buchan JR, Parker R (2009) Eukaryotic stress granules: the ins and outs of translation. *Mol Cell* 36: 932 – 941

Colonna M, Butovsky O (2017) Microglia function in the central nervous system during health and neurodegeneration. *Annu Rev Immunol* 35: 441 – 468

Dugger BN, Dickson DW (2017) Pathology of neurodegenerative diseases. *Cold Spring Harb Perspect Biol* 9: a028035

Farley MM, Watkins TA (2018) Intrinsic neuronal stress response pathways in injury and disease. *Annu Rev Pathol* 13: 93 – 116

Gasset-Rosa F, Lu S, Yu H, Chen C, Melamed Z, Guo L, Shorter J, Da Cruz S, Cleveland DW (2019) Cytoplasmic TDP-43 De-mixing independent of stress granules drives inhibition of nuclear import, loss of nuclear TDP-43, and cell death. *Neuron* 102: 339 – 357.e7

Ghosh S, Geahlen RL (2015) Stress granules modulate SYK to cause microglial cell dysfunction in Alzheimer's disease. *EBioMedicine* 2: 1785 – 1798

Hammond TR, Marsh SE, Stevens B (2019) Immune signaling in neurodegeneration. *Immunity* 50: 955 – 974

Heneka MT, Carson MJ, El Khoury J, Landreth GE, Brosseron F, Feinstein DL, Jacobs AH, Wyss-Coray T, Vitorica J, Ransohoff RM *et al* (2015) Neuroinflammation in Alzheimer's disease. *Lancet Neurol* 14: 388 – 405

Heneka MT, McManus RM, Latz E (2018) Inflammasome signalling in brain function and neurodegenerative disease. *Nat Rev Neurosci* 19: 610 – 621

Herman AB, Silva Afonso M, Kelemen SE, Ray M, Vrakas CN, Burke AC, Scalia RG, Moore K, Autieri MV (2019) Regulation of stress granule formation by inflammation, vascular injury, and atherosclerosis. *Arterioscler Thromb Vasc Biol* 39: 2014 – 2027

Hu S, Claud EC, Musch MW, Chang EB (2010) Stress granule formation mediates the inhibition of colonic Hsp70 translation by interferon-gamma and tumor necrosis factor-alpha. *Am J Physiol Gastrointest Liver Physiol* 298: G481 – G492

Ising C, Venegas C, Zhang S, Scheiblich H, Schmidt SV, Vieira-Saecker A, Schwartz S, Albasset S, McManus RM, Tejera D *et al* (2019) NLRP3 inflammasome activation drives tau pathology. *Nature* 575: 669 – 673

Ivanov P, Kedersha N, Anderson P (2019) Stress granules and processing bodies in translational control. *Cold Spring Harb Perspect Biol* 11: a032813

Iyer SS, Pulskens WP, Sadler JJ, Butter LM, Teske GJ, Ulland TK, Eisenbarth SC, Florquin S, Flavell RA, Leemans JC *et al* (2009) Necrotic cells trigger a sterile inflammatory response through the Nlrp3 inflammasome. *Proc Natl Acad Sci USA* 106: 20388 – 20393

Jonsson T, Stefansson H, Steinberg S, Jonsdottir I, Jonsson PV, Snaedal J, Bjornsson S, Huttenlocher J, Levey AI, Lah JJ *et al* (2013) Variant of TREM2 associated with the risk of Alzheimer's disease. *N Engl J Med* 368: 107 – 116

Kanneganti TD, Ozören N, Body-Malapel M, Amer A, Park JH, Franchi L, Whitfield J, Barchet W, Colonna M, Vandenabeele P *et al* (2006) Bacterial RNA and small antiviral compounds activate caspase-1 through cryopyrin/Nalp3. *Nature* 440: 233 – 236

Labzin LI, Heneka MT, Latz E (2018) Innate immunity and neurodegeneration. *Annu Rev Med* 69: 437 – 449

Lehnardt S (2010) Innate immunity and neuroinflammation in the CNS: the role of microglia in toll-like receptor-mediated neuronal injury. *Glia* 58: 253 – 263

Li Q, Barres BA (2018) Microglia and macrophages in brain homeostasis and disease. *Nat Rev Immunol* 18: 225 – 242

Mangan MSJ, Olhava EJ, Roush WR, Seidel HM, Glick GD, Latz E (2018) Targeting the NLRP3 inflammasome in inflammatory diseases. *Nat Rev Drug Discov* 17: 688

Marogianni C, Sokratous M, Dardiotis E, Hadjigeorgiou GM, Bogdanos D, Xiromerisiou G (2020) Neurodegeneration and inflammation-an interesting interplay in Parkinson's disease. *Int J Mol Sci* 21: 8421

Mayor A, Martinon F, De Smedt T, Pétrilli V, Tschopp J (2007) A crucial function of SGT1 and HSP90 in inflammasome activity links mammalian and plant innate immune responses. *Nat Immunol* 8: 497 – 503

McCauley ME, Baloh RH (2019) Inflammation in ALS/FTD pathogenesis. *Acta Neuropathol* 137: 715 – 730

McCormick C, Khaperskyy DA (2017) Translation inhibition and stress granules in the antiviral immune response. *Nat Rev Immunol* 17: 647 – 660

Mei F, Hu J, Wu Z, Zhang G, Liu A, Li X, Zhu M, Gan Y, Liang L, Zhao X *et al* (2022) FAM69C, a kinase critical for synaptic function and memory, is defective in neurodegenerative dementia. *Cell Rep* 40: 111101

 © 2023 The Authors

Pakos-Zebrucka K, Koryga I, Mnich K, Ljujic M, Samali A, Gorman AM (2016) The integrated stress response. *EMBO Rep* 17: 1374–1395

Protter DSW, Parker R (2016) Principles and properties of stress granules. *Trends Cell Biol* 26: 668–679

Samir P, Kesavardhana S, Patmore DM, Gingras S, Malireddi RKS, Karki R, Guy CS, Briard B, Place DE, Bhattacharya A *et al* (2019) DDX3X acts as a live-or-die checkpoint in stressed cells by regulating NLRP3 inflammasome. *Nature* 573: 590–594

Sonenberg N, Hinnebusch AG (2009) Regulation of translation initiation in eukaryotes: mechanisms and biological targets. *Cell* 136: 731–745

Song WM, Colonna M (2018) The identity and function of microglia in neurodegeneration. *Nat Immunol* 19: 1048–1058

Thedieck K, Holzwarth B, Prentzell MT, Boehlke C, Kläsener K, Ruf S, Sonntag AG, Maerz L, Grellscheid SN, Kremmer E *et al* (2013) Inhibition of mTORC1 by astrin and stress granules prevents apoptosis in cancer cells. *Cell* 154: 859–874

Vaquer-Alicea J, Diamond MI (2019) Propagation of protein aggregation in neurodegenerative diseases. *Annu Rev Biochem* 88: 785–810

Venegas C, Kumar S, Franklin BS, Dierkes T, Brinkschulte R, Tejera D, Vieira-Saecker A, Schwartz S, Santarelli F, Kummer MP *et al* (2017) Microglia-derived ASC specks cross-seed amyloid-β in Alzheimer's disease. *Nature* 552: 355–361

Voet S, Srinivasan S, Lamkanfi M, van Loo G (2019) Inflammasomes in neuroinflammatory and neurodegenerative diseases. *EMBO Mol Med* 11: e10248

Wolozin B, Ivanov P (2019) Stress granules and neurodegeneration. *Nat Rev Neurosci* 20: 649–666

Zhang J, Liu X, Wan C, Liu Y, Wang Y, Meng C, Zhang Y, Jiang C (2020) NLRP3 inflammasome mediates M1 macrophage polarization and IL-1β production in inflammatory root resorption. *J Clin Periodontol* 47: 451–460

License: This is an open access article under the terms of the Creative Commons Attribution License, which permits use, distribution and reproduction in any medium, provided the original work is properly cited.

© 2023 The Authors

Expanded View Figures

Figure EV1. FAM69C is a stress-specific kinase for eIF2α.

A Immunoblot analysis of phosphorylated eIF2α in SH-SY5Y cells. FAM69C$^{-/-}$ SH-SY5Y cells were transfected to express wildtype FAM69C and FAM69C D279N. Cells were treated with 0.5 mM AS for 30 min, and lysates were immunoblotted with the p-eIF2α (S51) antibody.

B Coomassie Blue Staining showed the protein level of BSA, FAM69C and D279N proteins used in the ADP-Glo kinase assay.

C Immunoblot analysis of phosphorylated eIF2α in HRI-knockdown SH-SY5Y cells. Cells were treated with or without 0.5 mM AS for 30 min, and lysates were immunoblotted with p-eIF2α (S51), eIF2α, HRI, and α-Tubulin antibody.

D Quantitative analysis of the phosphorylation level of eIF2α normalized with total eIF2α under AS treatment. **P* value < 0.05, paired *t*-test, *n* = 3 biologically independent experiments. Mean ± SD. For *FAM69C*$^{-/-}$ shHRI-1, *P* value = 0.0431; For *FAM69C*$^{-/-}$ shHRI-2, *P* value = 0.0337.

E Immunoblot analysis of phosphorylated eIF2α in PERK-knockdown SH-SY5Y cells. Cells were treated with 42°C heat shock (30 min), 4 μM TG (6 h). The cell lysates were immunoblotted with p-eIF2α (S51), eIF2α, PERK, and α-Tubulin antibody.

 © 2023 The Authors

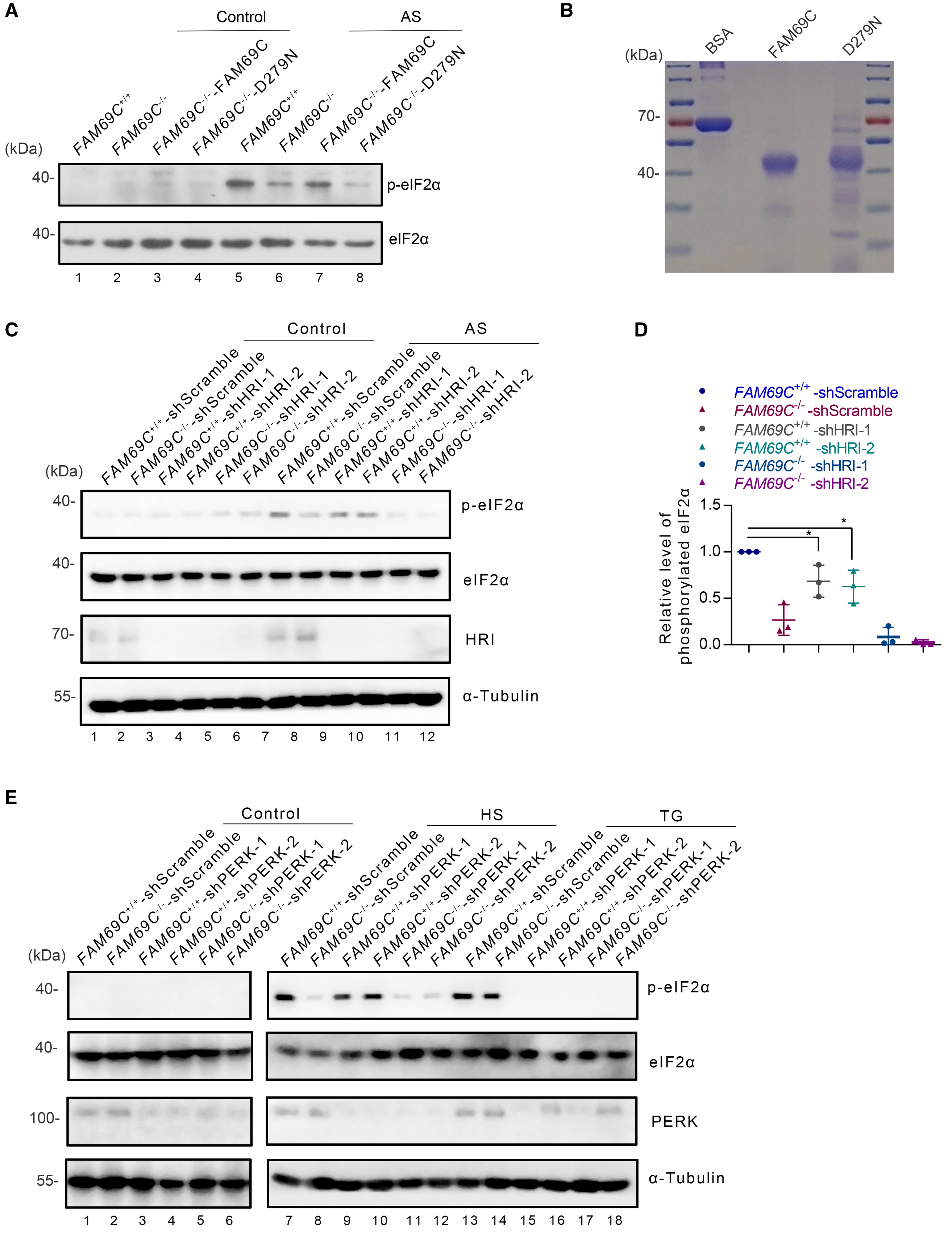
A
Control
AS
FAM69C+/+
FAM69C-/-
FAM69C-/--FAM69C
FAM69C-/--D279N
(kDa)
40-
p-eIF2α
eIF2α
1 2 3 4 5 6 7 8
B
(kDa)
BSA
FAM69C
D279N
70-
40-
C
Control
AS
FAM69C+/+-shScramble
FAM69C-/--shScramble
FAM69C+/+-shHRI-1
FAM69C+/+-shHRI-2
FAM69C-/--shHRI-1
FAM69C-/--shHRI-2
p-eIF2α
eIF2α
HRI
α-Tubulin
70-
55-
1 2 3 4 5 6 7 8 9 10 11 12
D
FAM69C+/+ -shScramble
FAM69C-/- -shScramble
FAM69C+/+ -shHRI-1
FAM69C+/+ -shHRI-2
FAM69C-/- -shHRI-1
FAM69C-/- -shHRI-2
Relative level of phosphorylated eIF2α
1.5
1.0
0.5
0.0
E
Control
HS
TG
FAM69C+/+-shScramble
FAM69C-/--shScramble
FAM69C+/+-shPERK-1
FAM69C+/+-shPERK-2
FAM69C-/--shPERK-1
FAM69C-/--shPERK-2
100-
PERK
1 2 3 4 5 6 7 8 9 10 11 12 13 14 15 16 17 18

Figure EV1.

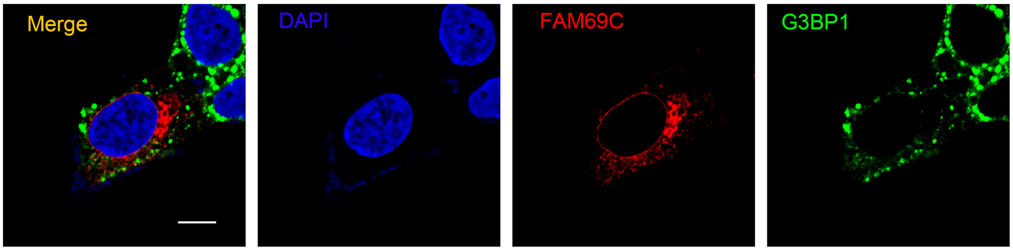

Figure EV2. Subcellular localization of FAM69C.

Confocal imaging of SH-SY5Y cells transfected with FAM69C and G3BP1, to visualize the localization of FAM69C and stress granules. Scale bar = 10 μm.

Figure EV3. Features in primary culture studies.

A Confocal imaging of isolated primary microglia. GFAP (Purple) was used as a marker of astrocyte, and Iba1 (Green) was used as a marker of microglia. All of the Iba1-positive microglia are ASC-positive (Red). Scale bar = 10 μm. Representative images, $n = 8$ fields from three biologically independent experiments.

B Immunoblot analysis of protein levels of core NLRP3 inflammasome components in primary cultured *Fam69c*$^{+/+}$ and *Fam69c*$^{-/-}$ microglia after LPS priming (1 μg/ml LPS for 3 h). Representative blots, $n = 3$ biologically independent experiments.

C Quantitative analysis of protein level of NLRP3, ASC and CASP1 normalized with β-Actin in *Fam69c*$^{+/+}$ and *Fam69c*$^{-/-}$ microglia. $n = 3$ biologically independent experiments. Mean ± SD.

D Confocal microscopy imaging of SH-SY5Y cells transfected with FAM69C and NLRP3, to visualize the localization of FAM69C and NLRP3 specks. Scale bar = 10 μm.

E Confocal imaging of primary cultured microglia treated with 1 μg/ml LPS (3 h), followed by 0.4 M sorbitol (1 h), to visualize the formation of stress granules. Representative images, $n = 3$ biologically independent experiments. Scale bar = 10 μm.

 © 2023 The Authors

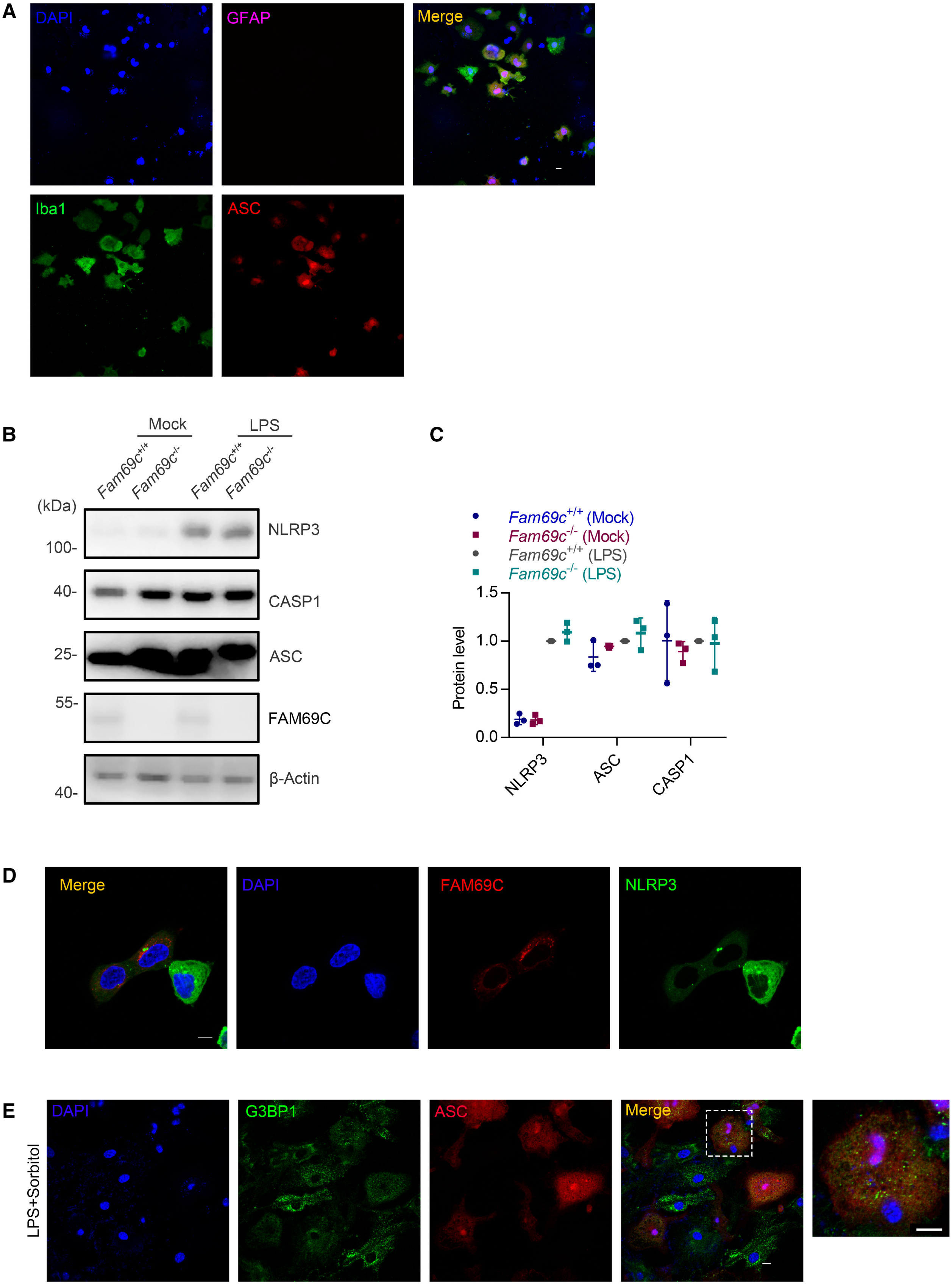
A
DAPI
GFAP
Merge
Iba1
ASC
B
Mock
LPS
Fam69c+/+
Fam69c-/-
Fam69c+/+
Fam69c-/-
(kDa)
100-
NLRP3
40-
CASP1
25-
ASC
55-
FAM69C
40-
β-Actin
C
Fam69c+/+ (Mock)
Fam69c-/- (Mock)
Fam69c+/+ (LPS)
Fam69c-/- (LPS)
Protein level
1.5
1.0
0.5
0.0
NLRP3
ASC
CASP1
D
Merge
DAPI
FAM69C
NLRP3
E
LPS+Sorbitol
DAPI
G3BP1
ASC
Merge

Figure EV3.

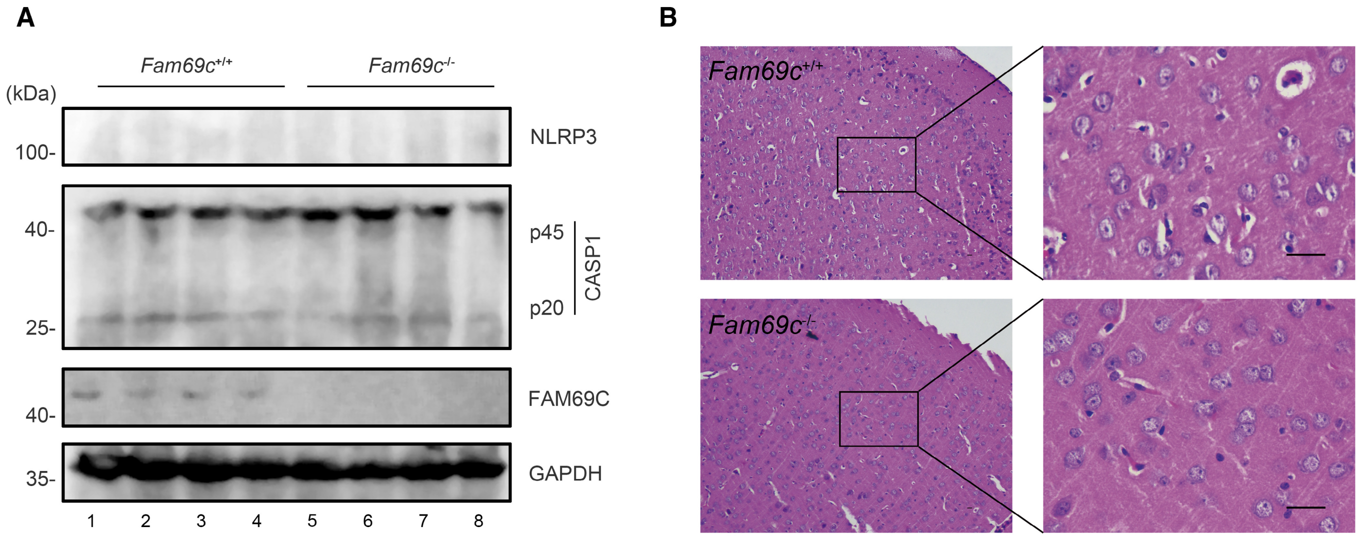

Figure EV4. Features in young mice.

A Immunoblot analysis of NLRP3 and cleaved caspase1 (p20) in cortex of 3-month-old *Fam69c*$^{+/+}$ and *Fam69c*$^{-/-}$ mice. Data are from four different mice with each genotype.

B Hematoxylin and eosin staining of 3-month-old *Fam69c*$^{+/+}$ and *Fam69c*$^{-/-}$ mouse brain. The image is representative of three pairs of mice. Scale bar = 25 μm.

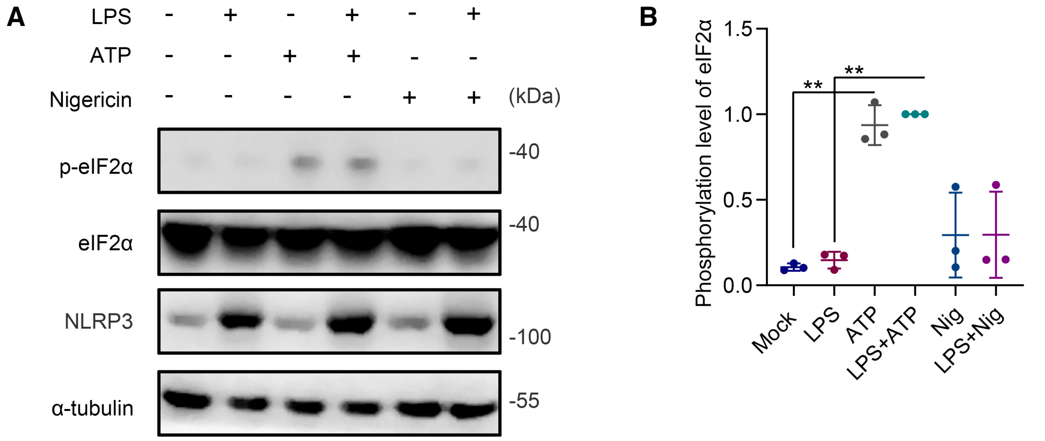

Figure EV5. ATP-induced phosphorylation of eIF2α in BV2 cells.

A Immunoblot analysis of phosphorylated eIF2α in BV2 cells. Primed (1 μg/ml LPS for 3 h) or unprimed BV2 cells were treated with mock, 5 mM ATP or 20 μM nigericin for 40 min, and lysates were immunoblotted with p-eIF2α (S51) antibody. Representative blots, $n = 3$ biologically independent experiments.

B Quantitative analysis of the phosphorylation level of eIF2α normalized with total eIF2α. **P value < 0.01, paired two-tailed t-test, $n = 3$ biologically independent experiments. Mean ± SD. For ATP versus mock, P value = 0.0047; For LPS + ATP versus LPS, P value = 0.0011.

 © 2023 The Authors
