# Supplementary material for: FAM69C functions as a kinase for eIF2α and promotes stress granule assembly
Source: EMBO Rep. 2023 Mar 16;24(5):e55641. doi: 10.15252/embr.202255641 (PMC10157376; doi:10.15252/embr.202255641)
Supplement: Supplementary file 4 [file EMBR-24-e55641-s003.pdf]

EMBO reports

FAM69C functions as a kinase for eIF2α and promotes stress granule assembly

Zhongyan Wu, Fan Mei, Yangyang Gan, Anhang Liu, Jiapan Hu, Yan Jin, and Yuxin Yin
DOI: 10.15252/embr.202255641

Corresponding author(s): Yuxin Yin (yinyuxin@hsc.pku.edu.cn)

Review Timeline:

Submission Date:	4th Jul 22
Editorial Decision:	8th Aug 22
Revision Received:	27th Dec 22
Editorial Decision:	3rd Feb 23
Revision Received:	8th Feb 23
Accepted:	16th Feb 23

Editor: Martina Rembold

Transaction Report:

(Note: With the exception of the correction of typographical or spelling errors that could be a source of ambiguity, letters and reports are not edited. Depending on transfer agreements, referee reports obtained elsewhere may or may not be included in this compilation. Referee reports are anonymous unless the Referee chooses to sign their reports.)

Dear Prof. Yin

Thank you for the submission of your research manuscript to our journal. We have now received the full set of referee reports that is copied below.

As you will see, all three referees acknowledge that the findings are potentially interesting. However, they also raise a number of largely overlapping concerns regarding missing quantification, statistical analysis, controls and the description of materials and methods. Please note that the deposition of RNA-seq and proteomics data in a public repository is mandatory as per our editorial policies.

The referees further point out that the role of FAM69C in stress granule biology seems overall convincing but the link to the inflammasome is less clear. I think that the referee suggestions are all relevant and should be addressed. It will important to substantiate the role of FAM69C as eIF2 kinase, its role in SG assembly and the link to inflammasome formation. In case the role in NLRP3 activation turns out to be indirect, the study should focus on the SG aspect and conclusions on the inflammasome should be toned down.

From the referee comments it is clear that a significant amount of work will be required to substantiate your conclusions. However, given the potential interest of your findings, I would like to give you the opportunity to address these concerns and would be willing to consider a revised manuscript with the understanding that the referee concerns must be fully addressed and their suggestions taken on board. Please address all referee concerns in a complete point-by-point response.

I am happy to discuss the revisions and the time required further in a Zoom call at your convenience, if you wish. Please contact me so that we can arrange a call.

Acceptance of the manuscript will depend on a positive outcome of a second round of review. I should add that that it is EMBO reports policy to allow a single round of revision only and acceptance or rejection of the manuscript will therefore depend on the completeness of your responses included in the next, final version of the manuscript.

We realize that it is difficult to revise to a specific deadline. In the interest of protecting the conceptual advance provided by the work, we recommend a revision within 3 months (November 8th). Having said so, I understand that the revision might take longer than that. Therefore, please contact me if you require more time.

*****IMPORTANT NOTE:
We perform an initial quality control of all revised manuscripts before re-review. Your manuscript will FAIL this control and the handling will be DELAYED if the following APPLIES:

1) A data availability section providing access to data deposited in public databases is missing. If you have not deposited any data, please add a sentence to the data availability section that explains that.

2) Your manuscript contains statistics and error bars based on n=2. Please use scatter blots in these cases. No statistics should be calculated if n=2.

When submitting your revised manuscript, please carefully review the instructions that follow below. Failure to include requested items will delay the evaluation of your revision.*****

When submitting your revised manuscript, we will require:

1) a .docx formatted version of the manuscript text (including legends for main figures, EV figures and tables). Please make sure that the changes are highlighted to be clearly visible.

2) individual production quality figure files as .eps, .tif, .jpg (one file per figure).
Please download our Figure Preparation Guidelines (figure preparation pdf) from our Author Guidelines pages https://www.embopress.org/page/journal/14693178/authorguide for more info on how to prepare your figures.

3) a .docx formatted letter INCLUDING the reviewers' reports and your detailed point-by-point responses to their comments. As part of the EMBO Press transparent editorial process, the point-by-point response is part of the Review Process File (RPF), which will be published alongside your paper.

4) a complete author checklist, which you can download from our author guidelines (). Please insert information in the checklist

that is also reflected in the manuscript. The completed author checklist will also be part of the RPF.

5) Please note that all corresponding authors are required to supply an ORCID ID for their name upon submission of a revised manuscript (). Please find instructions on how to link your ORCID ID to your account in our manuscript tracking system in our Author guidelines
()

6) We replaced Supplementary Information with Expanded View (EV) Figures and Tables that are collapsible/expandable online. A maximum of 5 EV Figures can be typeset. EV Figures should be cited as 'Figure EV1, Figure EV2" etc... in the text and their respective legends should be included in the main text after the legends of regular figures.

- For the figures that you do NOT wish to display as Expanded View figures, they should be bundled together with their legends in a single PDF file called *Appendix*, which should start with a short Table of Content. Appendix figures should be referred to in the main text as: "Appendix Figure S1, Appendix Figure S2" etc. See detailed instructions regarding expanded view here:

- Additional Tables/Datasets should be labeled and referred to as Table EV1, Dataset EV1, etc. Legends have to be provided in a separate tab in case of .xls files. Alternatively, the legend can be supplied as a separate text file (README) and zipped together with the Table/Dataset file.

7) Before submitting your revision, the primary datasets (RNA-seq, mass spec proteomics) produced in this study need to be deposited in an appropriate public database (see <
https://www.embopress.org/page/journal/14693178/authorguide#dataavailability>).

Please remember to provide a reviewer password if the datasets are not yet public.

The accession numbers and database should be listed in a formal "Data Availability " section (placed after Materials & Method) that follows the model below (see also < https://www.embopress.org/page/journal/14693178/authorguide#dataavailability>). Please note that the Data Availability Section is restricted to new primary data that are part of this study.

Data availability

The datasets (and computer code) produced in this study are available in the following databases:

- RNA-Seq data: Gene Expression Omnibus GSE46843 (https://www.ncbi.nlm.nih.gov/geo/query/acc.cgi?acc=GSE46843)
- [data type]: [name of the resource] [accession number/identifier/doi] ([URL or identifiers.org/DATABASE:ACCESSION])

*** Note - All links should resolve to a page where the data can be accessed. ***

8) The journal requires a statement specifying whether or not authors have competing interests (defined as all potential or actual interests that could be perceived to influence the presentation or interpretation of an article). In case of competing interests, this must be specified in your disclosure statement. Further information: https://www.embopress.org/competing-interests

9) Figure legends and data quantification:
The following points must be specified in each figure legend:

- the name of the statistical test used to generate error bars and P values,
- the number (n) of independent experiments (please specify technical or biological replicates) underlying each data point,
- the nature of the bars and error bars (s.d., s.e.m.)
- If the data are obtained from n {less than or equal to} 2, use scatter blots showing the individual data points.

Discussion of statistical methodology can be reported in the materials and methods section, but figure legends should contain a basic description of n, P and the test applied.

See also the guidelines for figure legend preparation:
https://www.embopress.org/page/journal/14693178/authorguide#figureformat

- Please also include scale bars in all microscopy images.

10) We would also encourage you to include the source data for figure panels that show essential data. Numerical data should be provided as individual .xls or .csv files (including a tab describing the data). For blots or microscopy, uncropped images should be submitted (using a zip archive if multiple images need to be supplied for one panel). Additional information on source data and instruction on how to label the files are available .

11) Our journal encourages inclusion of *data citations in the reference list* to directly cite datasets that were re-used and obtained from public databases. Data citations in the article text are distinct from normal bibliographical citations and should directly link to the database records from which the data can be accessed. In the main text, data citations are formatted as follows: "Data ref: Smith et al, 2001" or "Data ref: NCBI Sequence Read Archive PRJNA342805, 2017". In the Reference list, data citations must be labeled with "[DATASET]". A data reference must provide the database name, accession number/identifiers and a resolvable link to the landing page from which the data can be accessed at the end of the reference. Further instructions are available at .

12) As part of the EMBO publication's Transparent Editorial Process, EMBO reports publishes online a Review Process File to accompany accepted manuscripts. This File will be published in conjunction with your paper and will include the referee reports, your point-by-point response and all pertinent correspondence relating to the manuscript.

You are able to opt out of this by letting the editorial office know (emboreports@embo.org). If you do opt out, the Review Process File link will point to the following statement: "No Review Process File is available with this article, as the authors have chosen not to make the review process public in this case."

We would also welcome the submission of cover suggestions, or motifs to be used by our Graphics Illustrator in designing a cover.

I look forward to seeing a revised form of your manuscript when it is ready.

Please use this link to submit your revision: https://embor.msubmit.net/cgi-bin/main.plex

Yours sincerely,

Martina Rembold, PhD
Senior Editor
EMBO reports

****

Referee #1:

The manuscript by Wu and co-authors describes a very interesting finding in the cellular response to stress. The authors identified the brain-enriched kinase FAM69C as a suppressor of the NLRP3 inflammasome pathway activation in aged mice. They further propose that FAM69C regulates the major translation initiation factor eIF2α, thereby promoting stress granule (SG) assembly. Finally, they link SG assembly to suppression of the inflammasome pathway activation.
Although the results are novel and highlight a new and very interesting aspect of the regulation of the cellular response in the context of inflammatory responses, the authors fail to demonstrate the direct link between inflammasome and stress responses in the cell system used for the molecular characterization. In general, most conclusions are based on observations that have not been systematically quantified and for which the number of replications is not indicated. This weakens the manuscript considerably.

• The authors validated the up-regulation of candidate transcripts identified by RNA-Seq. Figure 1C and 1D show the relative expression level of Il18 and Il18r1 mRNAs. While Figure 1C represents the qPCR results, Figure 1D shows only an agarose gel. It is stated in the Materials and Methods section that this is a quantitative analysis. Results should be analyzed and presented in the same manner as in Figure 1C.

• Figure EV2A: It would be useful for the understanding of the non-specialist reader to have some additional explanations on the choice and function of markers, e.g. Iba1 or GFAP, the use of the latter not being mentioned in the main text. Here the authors conclude that all ASC-positive cells were Iba1-positive microglia. However, the representative image shows only one cell and no quantification is provided.

• The authors cultivate primary glial cells from Fam69+/+ and Fam69-/- mice to analyze ASC-speck and SG formation after priming with LPS and subsequent stimulation with ATP or nigericin (Figure 3A). The use of this model system is definitely relevant and important, however, the results are not supported by quantifications, which makes the conclusions questionable. To support their conclusions, the authors should provide analysis of unprimed cells, a control, and missing quantifications. For example, Figure 3B corresponds to the quantification of cells treated with LPS+ATP. The LPS and LPS+nigericin data sets are not quantified. Similarly, the authors conclude from Figure 3C that ASC-specks do not form in response to arsenite and ATP. Again, controls with arsenite and ATP only should be analyzed.

• The use of the term inflammasome in the label of the x-axis of the bar graph shown in Figure 3B is misleading and appears

incorrect to this reviewer. The authors mention that ATP and nigericin are common activators of the inflammasome, however SGs form in Fam69+/+ cells after addition of ATP but not nigericin. While the authors propose in the discussion that the impact of FAM69C is eIF2α-independent in the case of nigericin, no experimental evidence supports this conclusion. This calls into question the more general link made between SGs and the NLRP3 inflammasome.

• Figure 4A and 4B. The authors describe that LPS-primed BV2 cells treated with ATP or not form SGs, while SH-SY5Y cells do not. Here also quantifications should be provided. There is no direct evidence that these model systems recapitulate NRLP3 inflammasome pathways activation. The authors should provide ASC-speck and caspase-1 activity analysis. There is also no evidence that the observed SG formation in BV2 cells is linked to FAM69C expression. What is the level of expression FAM69C in these cells? We would expect BV2 cells to express less FAM69C than the SH-SY5Y cells.

• Figure 4C. The number of repeats and quantification of the immunoblot are missing.

• It is unclear what "mixed" Fam69-/- cells are.

• The live cell imaging experiment in SH-SY5Y Fam69-/- cells is very interesting and supports a role for FAM69C in the initiation phase of the integrated stress response, with delayed formation of SGs. Importantly, it also shows that it does not reduce cell responsiveness to stress. At 30 minutes after arsenite treatment, time at which global translation was measured by puromycin incorporation, all cells had mounted an SG response comparable to Fam69+/+ cells. However, the authors conclude that FAM69C depletion is accompanied by a partial block of translation. This conclusion is neither consistent with the live cell SG analysis at this time point, nor supported by the data presented in Figure 5C, which lacks quantification and number of repeats.

• The interaction of FAM69C with eIF2α is clearly novel and very interesting. This interaction is validated with endogenous eIF2α by IP. The authors propose that arsenite treatment increases this interaction but the system used is different and relies on cells that overexpress a tagged version of eIF2α whose levels seem to vary upon arsenite treatment. To reinforce their conclusion, the authors should repeat this experiment with endogenous eIF2α since they demonstrate that the IP is extremely effective. This avoids any problem of normalization.

• The authors analyze the subcellular localization of FAM69C by immunofluorescence analysis and show FAM69C colocalization with the medial Golgi marker Giantin (Fig. EV3A), but also with the cis-Golgi marker GM130, though this is not indicated in the main text. While panel EV3B seems to display colocalization fluorescence profiles of the analysis shown in EV3A, this panel is describe in the main text as a the distribution of a GFP fusion constructs. This data is not shown. The same confusion applies for panel 3C are these GFP fusion constructs? This is not explained in the legend. Why was Giantin co-expressed in the HeLa cells? The GFP without FAM69C peptide is not shown as control. Furthermore the authors do not describe the AA44-419 construct in the text. This section of the manuscript is not conclusive and related to the translation aspect.

• Phosphorylation levels of eIF2α in response to various ISR activators, including arsenite, heat shock, and thapsigarin are measured (Figure 6F). Beyond the fact that the results are not quantified and the number of replicates is not indicated, the authors do not comment on the absence of eIF2α phosphorylation in Fam69+/+ cells treated with thapsigargin (Figure 6F).

• Figure 6H: The level of FAM69C wild type and kinase-dead proteins used in the in vitro phosphorylation assay should be measured by Western blotting or gel Silver staining to confirm that lower luminescence in the kinase-dead samples is not resulting from a lower protein amount.

Minor comments:
• The use of SEMs instead of SDs is not justified for most analyses
• Fig EV1C volcano plot. It is impossible to guess the dot to which the names of the candidates shown are associated. It would be helpful to circle them and place the names outside the dot area. Candidates like cbp5 are listed but not mentioned in the text. Why are they interesting?
• Fig2B should show the individual data points as all other bar graphs.
• Figures 2A and EV1F: Labeling of immunoblot images is unclear. Do the different lanes correspond to individual mice? Repeats?
• "Interestingly, we observed a remarkable difference in inflammasome activation between Fam69c+/+ and Fam69c-/- microglia (ASC-positive) when treated with LPS and ATP, whereas no difference was observed when treated with LPS and nigericin (Fig. EV2B)." The authors likely refers to Fig. 3B since Fig. EV2B show SH-SY5Y cells.
• C57/BL6 murine replace for C57/BL6 mice
• In general, the figure legends are really poorly descriptive

Referee #2:

This manuscript attempts to assign a molecular function to the FAM69C kinase, a poorly characterized protein that plays a role in neurodegeneration. In their accompanying manuscript, the authors demonstrate that FAM69C is a serine/threonine kinase and present potential target proteins by phosphoproteomics and complementary approaches. Here, investigations into the role of FAM69C is extended to include the regulation of stress granule formation. The data demonstrating that stress granule formation is impaired in FAM69C-deficient cells is compelling. However, there are multiple concerns with the data presentation and analysis that should be addressed. Additionally, in the absence of more experimentation in eIF2-kinase deficient lines, the conclusion that FAM69C operates as a physiologically-relevant eIF2 kinase is an overstatement. While this manuscript covers and important topic that would be of interest to a broad readership, as it currently stands, there are multiple issues that should be addressed as follows:

- The underlying data for the sequencing experiment does not appear to be deposited in a public repository. Additionally, no mention was made of a supplementary table of the output from a differential expression analysis of RNAseq data. This is contrary to normal practice in the field and renders it impossible to assess these experiments
- One-tailed t-tests are used throughout the data analysis (for example Fig 1C). This is only appropriate if a directionality to changes in gene expression are pre-supposed. A two-tailed test is the norm. This would have impact on the reported significance of certain findings. For example: Fig2D would not reach significance of $P<0.05$ with a two-tailed test.
- Fig1 A-B: no RNAseq data available to asses
- Fig1C: there are two Pvalues in the figure legend and the *** annotation do not match between legend and figure
- Fig1D: why semi-quantitative RT-PCR? Why not RT-qPCR as performed for C?
- Fig 2B: there are two Pvalues in the figure legend with different numbers attributed to them
- Fig2D: the y axis is unclear. Is this percent or a in a certain number of fields of view?
- Fig2E: an arrowhead would be helpful to show the ASC speck so that it isn't erroneously compared to non-specific staining.
- Fig 5C: densitometry of the blot normalized against the GAPDH standard should be performed for three biological replicates. It is stated in the text that FAM69C-KO cells show a recovery of puromycin labeling over the parental line. It seems like it might be the case for clone1, but no so for clone2
- Fig 6A: the underlying data for the pulldown does not appear to be deposited in a public repository or reported as a supplementary table in the manuscript as is common practice. As such it renders it impossible to assess the experiment. However, since it is argued that FAM69C and eIF2alpha (EIF2S1) directly interact by immunoprecipitation, it was surprising not to see EIF2S1 come up in the figure. Without seeing evidence of EIF2S1 come down in this figure, it's unclear why a jump was made to assess direct interaction between eIF2alpha and FAM69C by IP.
- Fig 6B: the legend should mention that this analysis was based on RNAseq data (and the data should be accessible)
- Fig 6C: eIF2 is present in the negative control of the IP blot at about a quarter of the level as the actual IP, making the interpretation of this experiment less convincing.
- Fig 6D-E: which protein was pulled down? It isn't apparent from the labeling or the figure legend (both entries in the figure legend are identical even though the experiments are not). In either case, reciprocal pulldowns should be performed. There appears to be a mislabeling of Fig 6E - mistakenly shows that full length FAM69C is present. Also, these experiments rely on over-expression. An experiment using endogenous proteins should be warranted.
- Fig 6H: for clarity, y axis should include the word "relative". How does the sequence surrounding eIF2-Ser51 compare to the preferred substrate for FAM69C identified in the companion paper? Assessing eIF2 phosphorylation status when complementation of the knockout cell line with kinase-dead FAM69C should also be explored.
- Fig 6F: There appears to be an effect of FAM69C for arsenite and heatshock but not for thapsigargin (although TG did not elicit eIF2-P - which is strange). Is the thought that FAM69C is a stress-specific kinase that phosphorylates eIF2alpha? If this is the contention, then the role of FAM69C under these stimuli need to be assessed in the absence of the kinases that are currently known to be involved in the processes (HRI knockout for oxidative stress, PERK knockout for heatshock PMID: 32013014, and given that FAM69C interacts with PKR as shown in Fig 6A further complicates matters).
- Fig EV1C: although the source data isn't available, there appears to be a mis-interpretation regarding the expression of il18. It seems like it hasn't met any fold change cutoff (although it did meet the Padj<0.05 cutoff). If it lies between log2FC+/-1, then it isn't differentially expressed in this experiment. The figure legend states that all log2FC>0 is considered, which supports my concern in the interpretation of the data. Highlighting the points for each gene of interest on the volcano plot would also be helpful. Also, the Pvalue stated in the text (p-value<0.05) does not match with the description in the figure legend (p-value < 0.1). Also, the actual measure of significance should mentioned in the text and the figure legend: the adjusted P value (Padj = q-value) instead of p-value (which is before the differential gene expression calling software made corrections).
- Fig EV3A: why aren't all the golgi markers present in every cell? Where these transfected? Are antibodies to these native proteins unavailable? They are not listed in the antibody section of the materials and methods section.
- Typo: page 7. severer instead of severe
- Typo: it is stated: "whereas no difference was observed when treated with LPS and
- nigericin (Fig. EV2B)." it looks like EV2B only assesses stress granule formation in SY5Y cells upon nigericin (+/-LPS) treatment, not ASC specks and therefore doesn't comment on the inflammasome. It was probably meant to direct to the bottom panel of figure 3A, although the underlying assertion should be quantified
- in the text: "In addition, we also detected a significantly increased phosphorylation level of eIF2α in BV-2 cells when treated with ATP, which may responsible for SG assembly. (Figure. 4C)." The word significantly should be removed unless a statistical analysis of blot densitometry was performed and added to the manuscript.
- In the text: "western blotting revealed that protein translation was completely inhibited in response to AS treatment". Completely is much too strong, suggest change to "severely"
- In the text: "Western blotting revealed that levels of phosphorylated eIF2α at S51 were significantly lower in FAM69C-/- SH5Y

cells, as compared with FAM69C+/+ SH5Y cells (Figure. 6F)." Please strike "significantly" unless statistical analysis is being performed.
- Materials and methods should indicate how transfections were performed
- The description of bulk RNAseq analysis is insufficient. What were the sequencing conditions and what mapper was used?

Referee #3:

FAM69C is a putative kinase protein for which the cellular and molecular functions are not well defined. In this manuscript, Wu et al., demonstrate the role of FAM69C in stress granule formation and inflammasome regulation. The authors first demonstrated the inflammasome activation signals in aged mice lacking FAM69C expression, which encouraged them to test its role in microglial inflammasome activation and stress granule (SG) formation. The authors conclude that FAM69C interacts with eIF2a to promote its phosphorylation and SG formation. Also, the authors have tried to provide some clues showing in FAM69C-dependent regulation of inflammasome activation in microglial cells. FAM69C role in phosphorylating eIF2a and SGs formation is interesting, and the experimental data in this manuscript supports this concept and stands out as an important and only function of FAM69C. However, FAM69C role in inflammasome regulation is poor and the experimental data is not convincing. The authors tried to show that FAM69C mediated formation of SGs abolishes inflammasome activation (similar to the recent demonstration of DDX3X role in SGs and NLRP3 inflammasome regulation), however, there is no direct evidence for this claim. Please see my specific comments below.

1. Does FAM69C colocalize/recruit in SGs and inflammasome complexes? Is FAM69C a kinase to trigger SGs assembly, like PKR, but not the integral component of SGs? Why do authors show FAM69C localization to Golgi without any premise? Unlike the DDX3X role shown in previous studies, the FAM69C has an indirect role in inflammasome activation and do not participate in inflammasome activation. FAM69C mediated SG formation might sequester DDX3X and inhibits its role in NLRP3 inflammasome activation.
2. Based on the experimental data, it appears that FAM69C is predominantly a regulator of the SGs but not inflammasome activation. Does FAM69C regulate the priming or activation step of NLRP3 inflammasome activation? If FAM69C is primarily a kinase working at the level of translation, it affects the expression of NLRP3 inflammasome components (priming step) instead of the activation and assembly of the NLRP3 inflammasome. There is no data showing i) NLRP3, IL-1b and CASP1 expression levels in FAM69C-knockout microglia ii) NLRP3 ubiquitination and oligomerization in the presence and absence of FAM69C cells.
3. Figure-1: Authors considered the expression of IL-18 and its associated genes as a correlation to indicate inflammasome activation, which is incorrect. IL-18 expression is regulated by many inflammatory stimuli (PMID: 28468974).
4. Figure-2: In a recent study by the same authors (Cell Reports, 2022), memory impairment was observed in 3-6 months old mice deficient in FAM69C expression. This suggests the role of FAM69C in the early stages of the mice causing neurodegeneration or neuronal abnormalities. However, in this study, all the inflammasome activation signals reported are in 10-12-month old mice. 3-month old mice did not show any CASP1 cleavage signals. These experiments suggest that inflammasome activation is a mere consequence of established neurodegeneration or loss of neuronal homeostasis in the brain of the 3-6-month old mice. FAM69C may not be the trigger for micrgolia-mediated inflammation and subsequent neurodegeneration as it was previously observed in Alzheimer's and Parkinson's mouse models. Fig 2A- provide FAM69C expression blots. Fig 2E- This imaging data need ASC specks quantification to support the authors' conclusions. In addition, SG staining (G3BP1) in these sections is critical to understanding FAM69C role in coordinating stress granules and inflammasome activation in the cortex.
5. Figure-3: The experimental data and the conclusions in this figure are confusing and inconclusive. This data indicates that lack of FAM69C does not affect NLRP3 inflammasome activation but abolishes SGs formation. This suggests FAM69C role in SG activation but not in inflammasome regulation. It is known that LPS or LPS+ATP stimulation do not induce SG formation in BMDMs despite expressing the P2X4 receptor but induces robust inflammasome activation. The authors observed no inflammasome activation/ASC specks in WT-microglia (Fam69C+/+) but the presence of SGs after LPS+ATP treatment. Based on these observations, the authors concluded that ATP is a bonafide trigger for SGs in microglia, which is not convincing. Multiple triggers of NLRP3 inflammasome (imiquimod, silica or polyI:C+Nig) need to be tested to substantiate this claim. Also, in Fig-3C, FAM69C knock-out cells still show SG formation (in fact, more than WT cells) after arsenite and ATP treatment, contradicting the authors own conclusions.
6. Figure-4: Although authors claim that ATP induces SGs in microglial cells, SGs in BV-2 cells after ATP treatment is barely minimal and not appealing. Fig 4E- It is surprising to see the ASC speck count in cells treated with LPS and arsenite. Arsenite treatment abolishes inflammasome activation and ASC speck formation. Fig 4D- To substantiate the role of FAM69C in SG formation, authors need to use multiple triggers of SGs to prove this concept.
7. Figure-5: Do primary microglial cells lacking FAM69C expression show delay in SGs assembly or defects in SGs formation?? This is not clear in the manuscript. Also, in Fig 5C, the authors claim that FAM69C knock-out cells show partial differences in protein translation despite a significant difference similar to WT- cells. eIF2a phosphorylation blot is critical to understanding the blots in Fig 5C. Overall, FAM69C role in SG activation in neuronal cells appears to be critical and can be linked to its function in memory loss instead of its role in inflammasome activation.
8. "With the development of sequencing technology, the microglial-specific gene TREM2 R47H mutation identified with GWASs completely changed our understanding of the relationship between immunity and neurodegeneration". This sentence is exactly the same as the sentence in the introduction. Also, "SG and inflammasome assembly determine contrasting live-or-die fates of

stressed macrophages" is repeated.
9. Figure EV3: The figure panel citations and the representations are improper in the manuscript text.
10. Figure legends: Figures legends miss essential experimental information: mice age, time of arsenite treatment, time of collection of images.

Referee #1:

The manuscript by Wu and co-authors describes a very interesting finding in the cellular response to stress. The authors identified the brain-enriched kinase FAM69C as a suppressor of the NLRP3 inflammasome pathway activation in aged mice. They further propose that FAM69C regulates the major translation initiation factor eIF2α, thereby promoting stress granule (SG) assembly. Finally, they link SG assembly to suppression of the inflammasome pathway activation. Although the results are novel and highlight a new and very interesting aspect of the regulation of the cellular response in the context of inflammatory responses, the authors fail to demonstrate the direct link between inflammasome and stress responses in the cell system used for the molecular characterization. In general, most conclusions are based on observations that have not been systematically quantified and for which the number of replications is not indicated. This weakens the manuscript considerably.

We thank the reviewer for his/her critical and constructive comments, which helped us to improve our manuscript. We have systematically quantified the results and notified the number of replications and made the data more solid in the revised manuscript. In addition, the logic flow for this study has been adequately adjusted. First, we identified that FAM69C is a stress-specific kinase for eIF2α. Second, FAM69C promotes stress granule (SG) assembly through the phosphorylation of eIF2α. Third, ATP or arsenite (AS) treatment in microglia induces FAM69C-dependent SG assembly. Forth, we explored a potential mechanism that FAM69C-dependent SG assembly prevents NLRP3 inflammasome activation through the sequestration of DDX3X in SG. To this end, we have substantiated the role of FAM69C in stress granule assembly, toned down its correlation with reduced inflammasome and substantially revised the manuscript. A point-to-point response to the reviewer's comments is as following.

• *The authors validated the up-regulation of candidate transcripts identified by RNA-Seq. Figure 1C and 1D show the relative expression level of Il18 and Il18r1 mRNAs. While Figure 1C represents the qPCR results, Figure 1D shows only an agarose gel. It is stated in the Materials and Methods section that this is a quantitative analysis. Results should be analyzed and presented in the same manner as in Figure 1C.*
[Response] We thank the reviewer for this question. In **Figure 7C** in the revised manuscript, qPCR of both *Il18* and *Il18r1* was measured, suggesting elevated expression of *Il18* and *Il18r1* in aged *Fam69c*$^{-/-}$ mice.

• *Figure EV2A: It would be useful for the understanding of the non-specialist reader to have some additional explanations on the choice and function of markers, e.g. Iba1 or GFAP, the use of the latter not being mentioned in the main text. Here the authors conclude that all ASC-positive cells were Iba1-positive microglia. However, the*

representative image shows only one cell and no quantification is provided.
[Response] As suggested, we have notified that Iba1 is a molecular marker for microglia, and GFAP is a molecular marker for astrocyte in **Response Figure 1**. Moreover, quantification was made to support the claims that all ACS-positive cells were Iba1-positive microglia (**Response Figure 1**). In the revised manuscript, to substantiate the regulatory role of FAM69C in microglia, we did the primary microglia culture for all the immunofluorescence experiments (**Figures 3, 4, 5 and 6** in the revised manuscript).

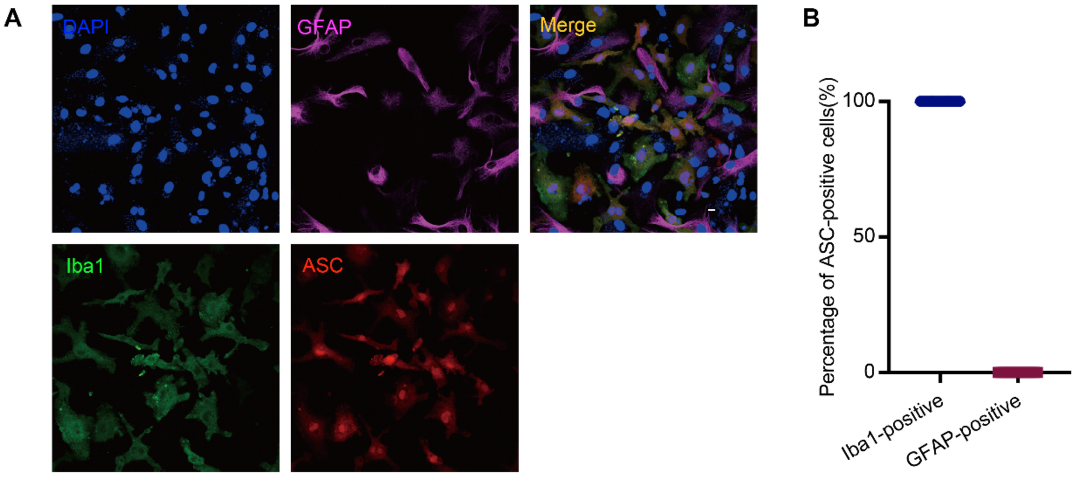

Response Figure 1. Immunostaining for astrocyte and microglia in primary glia culture. GFAP, for astrocyte; Iba1, for microglia.

• *The authors cultivate primary glial cells from Fam69+/+ and Fam69-/- mice to analyze ASC-speck and SG formation after priming with LPS and subsequent stimulation with ATP or nigericin (Figure 3A). The use of this model system is definitely relevant and important, however, the results are not supported by quantifications, which makes the conclusions questionable. To support their conclusions, the authors should provide analysis of unprimed cells, a control, and missing quantifications. For example, Figure 3B corresponds to the quantification of cells treated with LPS+ATP. The LPS and LPS+nigericin data sets are not quantified. Similarly, the authors conclude from Figure 3C that ASC-specks do not form in response to arsenite and ATP. Again, controls with arsenite and ATP only should be analyzed.*
[Response] The reviewer raised a series of questions related to missing controls and quantification. We have added quantification for each treatment (**Figure 3, 4, 5 and 6** in the revised manuscript). Unprimed cell control with vehicle was added (**Figure 3A** in the revised manuscript), in which neither SG formation nor ASC speck was observed. Moreover, *Fam69c*$^{+/+}$ and *Fam69c*$^{-/-}$ microglia treated with AS (**Figure 3B** in the revised manuscript), ATP (**Figure 3C** in the revised manuscript), nigericin (**Figure 5A** in the revised manuscript) were included, analyzed and quantified in the revised manuscript. Together, these data suggested that either AS or ATP elicited

stress granule assembly in the *Fam69c*$^{+/+}$ microglia, which is defected in the *Fam69c*$^{-/-}$ microglia. By contrast, nigericin treatment was not able to induce SG formation and instead triggered inflammasome activation in both *Fam69c*$^{+/+}$ and *Fam69c*$^{-/-}$ microglia.

• *The use of the term inflammasome in the label of the x-axis of the bar graph shown in Figure 3B is misleading and appears incorrect to this reviewer. The authors mention that ATP and nigericin are common activators of the inflammasome, however SGs form in Fam69+/+ cells after addition of ATP but not nigericin. While the authors propose in the discussion that the impact of FAM69C is eIF2α-independent in the case of nigericin, no experimental evidence supports this conclusion. This calls into question the more general link made between SGs and the NLRP3 inflammasome.*
[Response] We apologize for the misleading presentation of the data in the original manuscript. Nigericin is a common activator of inflammasome, but is not capable of inducing SG formation. We observed that nigericin treatment induced inflammasome formation both in LPS-primed *Fam69c*$^{+/+}$ and *Fam69c*$^{-/-}$ microglia (**Figure 5B** in the revised manuscript). By contrast, ATP only induced inflammasome activation in *Fam69c*$^{-/-}$ microglia but not in *Fam69c*$^{+/+}$ microglia. This is due to ATP-elicited SG assembly and restrained inflammasome formation. (**Figure 4B** in the revised manuscript). Given that phosphorylation of eIF2α acts as the trigger for SG assembly, we tested phosphorylated eIF2α levels both under ATP and nigericin treatment. Consistent with immunofluorescence data of SG assembly, we observed increased phosphorylated eIF2α levels upon ATP treatment but not upon nigericin treatment (**Figure EV5** in the revised manuscript). As for the general link between SG and the NLRP3 inflammasome, our study is in consistence with previous findings that assembly of stress granule inhibits NLRP3 inflammasome activation[1]. The underlying mechanism relates to the sequestration of DDX3X in the SG prevents its interaction with NLRP3 and the following inflammasome activation. Consistently, we found that ATP treatment in microglia sequestered DDX3X in the SG, which may explain the restrain of inflammasome formation (**Figure 4D** in the revised manuscript).

• *Figure 4A and 4B. The authors describe that LPS-primed BV2 cells treated with ATP or not form SGs, while SH-SY5Y cells do not. Here also quantifications should be provided. There is no direct evidence that these model systems recapitulate NRLP3 inflammasome pathways activation. The authors should provide ASC-speck and caspase-1 activity analysis. There is also no evidence that the observed SG formation in BV2 cells is linked to FAM69C expression. What is the level of expression FAM69C in these cells? We would expect BV2 cells to express less FAM69C than the SH-SY5Y cells.*
[Response] We thank the reviewer for this question. According to the reviewer's suggestion, we have measured NLRP3 inflammasome activation by western blotting (**Response Figure 2**, see below). Although LPS priming augmented NLRP3 expression in BV2 cells, lack of ASC expression suggested that the measurement of

NLRP3-ASC-caspase 1 activation is impossible (**Response Figure 2**, see below). Thus, we substituted BV2 cells with primary cultured microglia from *Fam69c*$^{+/+}$ and *Fam69c*$^{-/-}$ mice, and demonstrated the role of FAM69C in microglia (**Figure 3, 4, 5 and 6**, **Figure EV3B** in the revised manuscript). Second, SH-SY5Y cell line is not a myeloid cell line and is not suitable for the study of the regulatory effect of FAM69C in microglia. To this end, we applied primary microglia culture for our study.

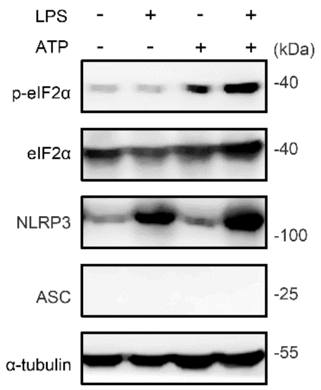

Response Figure 2. Western blotting of NLRP3 and ASC levels in BV2 cells.

• *Figure 4C. The number of repeats and quantification of the immunoblot are missing.*
[Response] We are grateful for this suggestion. The number of repeats and quantification of the immunoblot have been added (**Figure EV5** in the revised manuscript).

• *It is unclear what "mixed" Fam69-/- cells are.*
[Response] We apologize for the misunderstanding. It meant a mixed primary culture of astrocyte and microglia. To specify the role of microglia, we did the primary culture of microglia and detailed it in the material and method section in the revised manuscript. We examined how microglia react to stress and the role of FAM69C in the regulation of stress response. (**Figures 3, 4, 5 and 6** in the revised manuscript).

• *The live cell imaging experiment in SH-SY5Y Fam69-/- cells is very interesting and supports a role for FAM69C in the initiation phase of the integrated stress response, with delayed formation of SGs. Importantly, it also shows that it does not reduce cell responsiveness to stress. At 30 minutes after arsenite treatment, time at which global translation was measured by puromycin incorporation, all cells had mounted an SG response comparable to Fam69+/+ cells. However, the authors conclude that FAM69C depletion is accompanied by a partial block of translation. This conclusion is neither consistent with the live cell SG analysis at this time point, nor supported by the data presented in Figure 5C, which lacks quantification and number of repeats.*
[Response] We are very grateful for the reviewer on this comment. We have quantified this puromycin incorporation experiment and notified number of repeats (**Figure 2B** in the revised manuscript). Moreover, we measured levels of phosphorylated eIF2α together with puromycin incorporation, to show an aberrant

block of protein synthesis in response to arsenite treatment in FAM69C$^{-/-}$ cells (**Figure 2A** in the revised manuscript).

The experimental system used in Figure 5A and Figure 5C in the original manuscript is quite different and thus not comparable in terms of time point. In Figure 5A, we stably overexpressed G3BP1-GFP in *FAM69C*$^{+/+}$ and *FAM69C*$^{-/-}$ cells and treated them with arsenite for live-cell imaging of stress granule formation. The velocity of SG formation in cells with stable expression of G3BP1-GFP in Figure 5A did not resemble the endogenous situation that measured in Figure 5C. We thus thought these are two different experimental settings and not comparable.

• *The interaction of FAM69C with eIF2α is clearly novel and very interesting. This interaction is validated with endogenous eIF2α by IP. The authors propose that arsenite treatment increases this interaction but the system used is different and relies on cells that overexpress a tagged version of eIF2α whose levels seem to vary upon arsenite treatment. To reinforce their conclusion, the authors should repeat this experiment with endogenous eIF2α since they demonstrate that the IP is extremely effective. This avoids any problem of normalization.*

[Response] We agreed with the reviewer that the interaction of FAM69C with eIF2α is strong. As suggested, we have done the IP experiment with endogenous eIF2α and demonstrated that arsenite treatment increased the interaction between FAM69C and eIF2α (**Figure 1B** in the revised manuscript). Quantification of this IP experiment is provided in **Figure 1C** in the revised manuscript.

• *The authors analyze the subcellular localization of FAM69C by immunofluorescence analysis and show FAM69C colocalization with the medial Golgi marker Giantin (Fig. EV3A), but also with the cis-Golgi marker GM130, though this is not indicated in the main text. While panel EV3B seems to display colocalization fluorescence profiles of the analysis shown in EV3A, this panel is describe in the main text as a the distribution of a GFP fusion constructs. This data is not shown. The same confusion applies for panel 3C are these GFP fusion constructs? This is not explained in the legend. Why was Giantin co-expressed in the HeLa cells? The GFP without FAM69C peptide is not shown as control. Furthermore the authors do not describe the AA44-419 construct in the text. This section of the manuscript is not conclusive and related to the translation aspect.*

[Response] We apologize for the missing information of experiments details. In Figure EV3A in the original manuscript, SH-SY5Y cells with stably transfected HA-tagged FAM69C were used to examine the subcellular localization of FAM69C. Figure EV3B is the fluorescence intensity analysis for colocalization of FAM69C and Golgi marker in Figure EV3A. We are sorry for the error of mislabeling. In Figure EV3C in the original manuscript, SH-SY5Y cells were co-transfected of GFP-tagged full-length FAM69C or GFP-tagged FAM69C truncations with Scarlet-Giantin as medial Golgi marker. Co-localization of FAM69C and Giantin was examined. As suggested, the GFP without FAM69C is shown in the **Response Figure 3**. The AA44-

419 construct was the lumenal FAM69C truncation and its localization is unrelated to the study and thus removed in the revised manuscript.

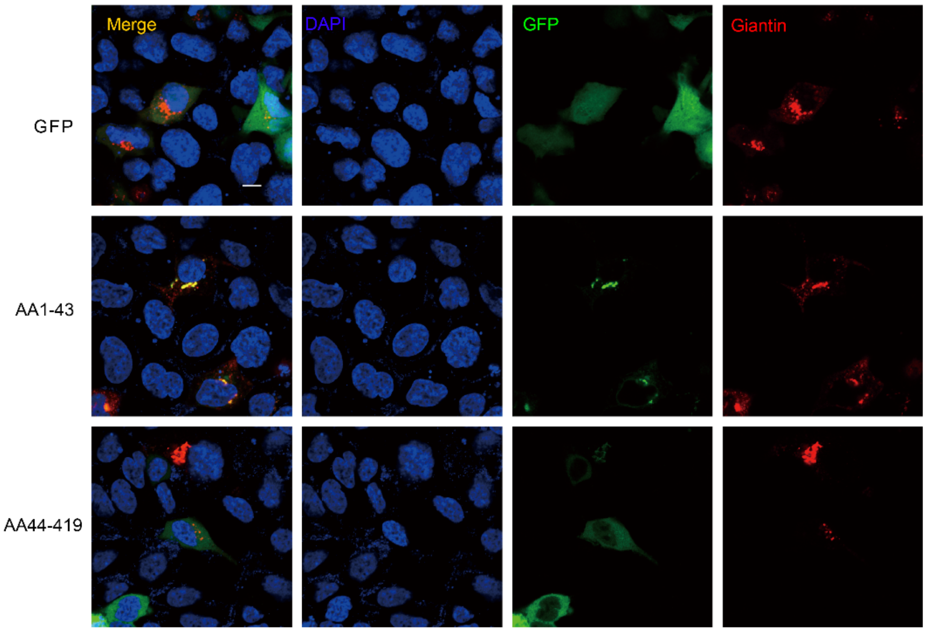

Response Figure 3. Immunofluorescence of SH-SY5Y cells transfected GFP or GFP-tagged FAM69C truncation together with Scarlet-Giantin.

• *Phosphorylation levels of eIF2α in response to various ISR activators, including arsenite, heat shock, and thapsigargin are measured (Figure 6F). Beyond the fact that the results are not quantified and the number of replicates is not indicated, the authors do not comment on the absence of eIF2α phosphorylation in Fam69+/+ cells treated with thapsigargin (Figure 6F).*

[Response] We are very grateful for the reviewer on this comment. We have quantified the results and notified the number of replicates in revision (**Figure 1D and 1E** in the revised manuscript). In response to arsenite and heat shock, we observed increased levels of phosphorylated eIF2α in *FAM69C*$^{+/+}$ cells, but not in *FAM69C*$^{-/-}$ cells. In Figure 6F in the original manuscript, we treated the cells with 1μM thapsigargin for 30 minutes, which is a rather weak stimulation for induction of eIF2α phosphorylation. In the revised manuscript, we treated the cells with 4μM thapsigargin for 6 hours. Although thapsigargin treatment obviously increased phosphorylated eIF2α, no difference was observed between *FAM69C*$^{+/+}$ and *FAM69C*$^{-/-}$ cells (**Figure EV1D**, lane 13 versus 14, in the revised manuscript). To determine the kinase that is responsible for eIF2α phosphorylation upon thapsigargin treatment, we knocked down *PERK*, one of the known eIF2α kinases, in *FAM69C*$^{+/+}$ and *FAM69C*$^{-/-}$ cells, and observed that phosphorylation of eIF2α was completely abolished (**Figure EV1D, lanes 15, 16, 17 and 18 versus lanes 13, 14**, in the revised manuscript). These data suggest that PERK is the kinase responsible for the phosphorylation of eIF2α under thapsigargin treatment. We also examined the effect of PERK on the phosphorylation of eIF2α under heat shock, and found that knockdown of *PERK* did not eliminate phosphorylated eIF2α (**Figure EV1D, lanes 7-**

12, in the revised manuscript). These data indicated that FAM69C, but not PERK, is the kinase that phosphorylates eIF2α under heat shock. Moreover, we knocked down *HRI*, another known kinase for eIF2α, in *FAM69C*$^{+/+}$ and *FAM69C*$^{-/-}$ cells. We found that either downregulation of HRI or FAM69C reduced phosphorylated eIF2α in response to arsenite treatment (**Figure EV1C**, lanes 7 and 8 versus lanes 9-12, in the revised manuscript). Taken together, these data suggest that FAM69C is responsible for eIF2α phosphorylation upon arsenite and heat shock, but not thapsigargin.

• *Figure 6H: The level of FAM69C wild type and kinase-dead proteins used in the in vitro phosphorylation assay should be measured by Western blotting or gel Silver staining to confirm that lower luminescence in the kinase-dead samples is not resulting from a lower protein amount.*
[Response] As suggested, we have measured the levels of wild-type FAM69C and kinase-dead proteins by gel Silver staining (**Figure EV1B** in the revised manuscript)

Minor comments:
• *The use of SEMs instead of SDs is not justified for most analyses*
[Response] We thank the reviewer for this suggestion and have used SDs in the revised manuscript.

• *Fig EV1C volcano plot. It is impossible to guess the dot to which the names of the candidates shown are associated. It would be helpful to circle them and place the names outside the dot area. Candidates like cbp5 are listed but not mentioned in the text. Why are they interesting?*
[Response] We thank the reviewer for this suggestion. We have labeled the genes that are indicative of increased innate immune response in the aged *Fam69c*$^{-/-}$ mice (**Figures for referees not shown**). In the revised manuscript, we substituted the volcano plot with GSEA analyses to more comprehensively represent transcriptional changes (**Figures for referees not shown**).

• *Fig2B should show the individual data points as all other bar graphs.*
[Response] We thank the reviewer for this suggestion and have shown the individual data points as all other bar graphs (**Figures 7A and 7B** in the revised manuscript).

• *Figures 2A and EV1F: Labeling of immunoblot images is unclear. Do the different lanes correspond to individual mice? Repeats?*
[Response] We have relabeled the immunoblot images, delineated each lane corresponding to each individual mouse and notified repeats of experiment in the revised figure legend (**Figure** 7A in the revised manuscript).

• *"Interestingly, we observed a remarkable difference in inflammasome activation between Fam69c+/+ and Fam69c-/- microglia (ASC-positive) when treated with LPS and ATP, whereas no difference was observed when treated with LPS and nigericin*

(Fig. EV2B)." The authors likely refers to Fig. 3B since Fig. EV2B show SH-SY5Y cells.
[Response] We apologize for the error and have corrected it in the revised manuscript (**Figures 4 and 5** in the revised manuscript).

• C57/BL6 murine replace for C57/BL6 mice
[Response] We have removed this description in the revised manuscript.

• In general, the figure legends are really poorly descriptive
[Response] We have rewritten the figure legends and made them explicit.

Referee #2:

This manuscript attempts to assign a molecular function to the FAM69C kinase, a poorly characterized protein that plays a role in neurodegeneration. In their accompanying manuscript, the authors demonstrate that FAM69C is a serine/threonine kinase and present potential target proteins by phosphoproteomics and complementary approaches. Here, investigations into the role of FAM69C is extended to include the regulation of stress granule formation. The data demonstrating that stress granule formation is impaired in FAM69C-deficient cells is compelling. However, there are multiple concerns with the data presentation and analysis that should be addressed. Additionally, in the absence of more experimentation in eIF2-kinase deficient lines, the conclusion that FAM69C operates as a physiologically-relevant eIF2 kinase is an overstatement. While this manuscript covers and important topic that would be of interest to a broad readership, as it currently stands, there are multiple issues that should be addressed as follows:

We thank the reviewer for his/her critical and constructive comments, which helped us to improve our manuscript. As suggested, we have substantially improved data presentation and quantification in the revised manuscript. Moreover, we have knocked down *HRI* and *PERK* in *FAM69C*$^{+/+}$ and *FAM69C*$^{-/-}$ cells and compared the effect of FAM69C, HRI and PERK on phosphorylation of eIF2α under different stress conditions. Based on these data, we suggest that FAM69C is a novel and physiologically-relevant eIF2α kinase under oxidative stress and heat shock, but not related to ER stress. Moreover, as suggested by the editor, we have substantiated the role of FAM69C in stress granule assembly, toned down its indirect correlation with reduced inflammasome and comprehensively revised the manuscript. A point-to-point response to the reviewer’s comments is as following.

- The underlying data for the sequencing experiment does not appear to be deposited in a public repository. Additionally, no mention was made of a supplementary table of

the output from a differential expression analysis of RNAseq data. This is contrary to normal practice in the field and renders it impossible to assess these experiments
[Response] We thank the reviewer for this question. We have deposited the RNA sequencing data in the GEO database with the accession code GSE214646. We also included a supplementary table of the differential expression gene (**Supplementary Table 2** in the revised manuscript).

- One-tailed t-tests are used throughout the data analysis (for example Fig 1C). This is only appropriate if a directionality to changes in gene expression are pre-supposed. A two-tailed test is the norm. This would have impact on the reported significance of certain findings. For example: Fig2D would not reach significance of P<0.05 with a two-tailed test.
[Response] We thank the reviewer for this question. In the revised manuscript, we have applied two-tailed t-tests, instead of one-tailed t-test in the original manuscript. To improve data quality, we have increased the sample number in several experiments (for example, **Figure 7E** in the revised manuscript).

- Fig1 A-B: no RNAseq data available to asses
[Response] We have deposited the RNA sequencing data in GEO database with the accession code GSE214646.

*- Fig1C: there are two Pvalues in the figure legend and the *** annotation do not match between legend and figure*
[Response] We apologize for this mistake and have corrected it in the revised manuscript.

- Fig1D: why semi-quantitative RT-PCR? Why not RT-qPCR as performed for C?
[Response] We thank the reviewer for this question. qPCR was performed for *Il18r1*. The data revealed increased *Il18r1* expression in the aged *Fam69c*$^{-/-}$ mice (**Figure 7C** in the revised manuscript).

- Fig 2B: there are two Pvalues in the figure legend with different numbers attributed to them
[Response] We thank the reviewer for this question and have corrected this error in the revised manuscript.

- Fig2D: the y axis is unclear. Is this percent or a in a certain number of fields of view?
[Response] We apologize for this misunderstanding and have corrected it to "number of ASC positive cells per field" (**Figure 7E** in the revised manuscript).

- Fig2E: an arrowhead would be helpful to show the ASC speck so that it isn't erroneously compared to non-specific staining.

[Response] To better illustrate ASC expression in the microglia from the mPFC of *Fam69*$^{+/+}$ and *Fam69c*$^{-/-}$ mice, we have substituted Figure 2E in the original manuscript with **Figures 7D and 7E** in the revised manuscript.

- Fig 5C: densitometry of the blot normalized against the GAPDH standard should be performed for three biological replicates. It is stated in the text that FAM69C-KO cells show a recovery of puromycin labeling over the parental line. It seems like it might be the case for clone1, but no so for clone2

[Response] We thank the reviewer for this question. We have normalized puromycin incorporation against the internal control (**Figures 2A and 2B** in the revised manuscript). Moreover, we have examined the levels of phosphorylated eIF2α, which was inconsistent with the puromycin incorporation experiment and indicated defected block of protein translation in *FAM69C*$^{-/-}$ clone 1 and clone 2 upon AS treatment. The number of biological replicates was indicated in the figure legend.

- Fig 6A: the underlying data for the pulldown does not appear to be deposited in a public repository or reported as a supplementary table in the manuscript as is common practice. As such it renders it impossible to assess the experiment. However, since it is argued that FAM69C and eIF2alpha (EIF2S1) directly interact by immunoprecipitation, it was surprising not to see EIF2S1 come up in the figure. Without seeing evidence of EIF2S1 come down in this figure, it's unclear why a jump was made to assess direct interaction between eIF2alpha and FAM69C by IP.

[Response] We thank the reviewer for these questions. We have deposited the mass spectrometry data for the pulldown analyses in the ProteomeXchange Consortium via the PRIDE partner repository with the dataset identifiers PXD037697. A supplementary table for the pulldown assay was also provided (**Supplementary Table 1** in the revised manuscript). In **Figure 1A** in the revised manuscript, *EIF2S1* was labeled. Given that *EIF2S1* is involved in stress granule assembly, regulation of translation in response to stress, and served as a regulator of cytoplasmic translation and ribonucleoprotein complex assembly, we selected *EIF2S1*-encoded eIF2α as a candidate and tested its direct interaction with FAM69C by immunoprecipitation.

- Fig 6B: the legend should mention that this analysis was based on RNAseq data (and the data should be accessible)

[Response] According to the reviewer's suggestion, we have mentioned that this GSEA analysis was based on RNA sequencing data, which has been deposited in GEO database with the accession code GSE214646.

- Fig 6C: eIF2 is present in the negative control of the IP blot at about a quarter of the level as the actual IP, making the interpretation of this experiment less convincing.

[Response] We thank the reviewer for this question. In Figure 6C in the original manuscript, we used a wash buffer with low concentration of detergent (150 mM NaCl, 1 mM EDTA, 20 mM Tris-HCl pH 8.0 and 0.1% NP40), which resulted in

unspecific protein binding with S-tag beads. We have applied a more stringent washing condition with 0.3% NP40, which led to much less unspecific protein binding with S-tag beads. As shown in **Figures 1B and 1C** in the revised manuscript, the IP experiment indicated an evident interaction of FAM69C with eIF2α and this physical interaction was strengthened upon AS treatment.

- Fig 6D-E: which protein was pulled down? It isn't apparent from the labeling or the figure legend (both entries in the figure legend are identical even though the experiments are not). In either case, reciprocal pulldowns should be performed. There appears to be a mislabeling of Fig 6E - mistakenly shows that full length FAM69C is present. Also, these experiments rely on over-expression. An experiment using endogenous proteins should be warranted.
[Response] We apologize for this mistake. In Figures 6D and 6E in the original manuscript, we performed S-tag beads pulldown, suggesting S-tagged FAM69C was pulled down and probed for the Flag-tagged eIF2α. In the revised manuscript, we did this immunoprecipitation experiment with endogenous eIF2α (**Figure 1B** in the revised manuscript). Figure 6E relates to a truncated FAM69C, which is unrelated to the study, and thus removed in the revised manuscript.

- Fig 6H: for clarity, y axis should include the word "relative". How does the sequence surrounding eIF2-Ser51 compare to the preferred substrate for FAM69C identified in the companion paper? Assessing eIF2 phosphorylation status when complementation of the knockout cell line with kinase-dead FAM69C should also be explored.
[Response] We have corrected the Y axis for this data as "relative luminescence" (**Figure 1G** in the revised manuscript). In the companion paper, mass spectrometry analyses of the *in vitro* phosphorylation using recombinant FAM69C protein and peptide library generated from human cortical tissue revealed that FAM69C is a serine/threonine kinase. Specifically, FAM69C phosphorylates peptides containing pSer-Pro, pSer-Glu, pSer-Arg, pSer-Leu, pSer-Lys, pSer-Asp, pSer-Asn, pSer-Thr, pSer-Ser, pSer-Gln, pSer-ILE, pSer-Ala, pSer-Leu, pSer-Val and pSer-Gly. Based on these motif analyses, eIF2α-Ser51 belongs to the predicted substrates for FAM69C. Indeed, western analysis showed that FAM69C phosphorylated eIF2α both in the *in vivo* and *in vitro* experimental settings (**Figures 1D, 1E, 1F, and 1G** in the revised manuscript). Moreover, *FAM69C*$^{-/-}$ cells were transfected with wild-type FAM69C or the kinase-dead mutation D279N (**Figure EV1A** in the revised manuscript). Wild-type FAM69C increased levels of phosphorylated eIF2α in *FAM69C*$^{-/-}$ cells upon AS treatment, whereas the D279N mutation did not phosphorylate eIF2α (**Figure EV1A**, lane 7 versus lane 8 in the revised manuscript).

- Fig 6F: There appears to be an effect of FAM69C for arsenite and heatshock but not for thapsigargin (although TG did not elicit eIF2-P - which is strange). Is the thought that FAM69C is a stress-specific kinase that phosphorylates eIF2alpha? If this is the contention, then the role of FAM69C under these stimuli need to be assessed in the

absence of the kinases that are currently known to be involved in the processes (HRI knockout for oxidative stress, PERK knockout for heatshock PMID: 32013014, and given that FAM69C interacts with PKR as shown in Fig 6A further complicates matters).

[Response] We thank the reviewer for this question. The following efforts were made to address this concern. First, we knocked down HRI in the *FAM69C*$^{+/+}$ and *FAM69C*$^{-/-}$ cells and tested the levels of phosphorylated eIF2α (p-eIF2α) (**Figure EV1C** in the revised manuscript). Western blotting showed that lack of FAM69C substantially reduced p-eIf2α, albeit some levels of p-eIf2α remains (**Figure EV1C**, lane 7 versus lane 8, in the revised manuscript). Further reduction of FAM69C abolished p-eIf2α (**Figure EV1C**, lanes 11, 12 versus lanes 9, 10, in the revised manuscript). These data indicated that both FAM69C and HRI are the physiologically-relevant kinases that phosphorylates eIF2α under AS-induced oxidative stress. Second, we knocked down *PERK* in the *FAM69C*$^{+/+}$ and *FAM69C*$^{-/-}$ cells. Upon heat shock, levels of p-eIF2α were significantly increased in *FAM69C*$^{+/+}$ cells, compared with that in *FAM69C*$^{-/-}$ cells (**Figure EV1D**, lane 7 versus lane 8, in the revised manuscript). Further, knockdown of *PERK* did not impose much effect on the levels of p- eIF2α (**Figure EV1D**, lanes 9, 10 versus lanes 11, 12, in the revised manuscript). These data revealed that FAM69C is responsible for phosphorylating eIF2α upon heat shock, whereas PERK was not. Third, we tested the kinase for eIF2α under ER stress. In thapsigargin treatment, levels of p-eIF2α were considerably increased and loss of FAM69C did not make any changes in p- eIF2α levels (**Figure EV1D**, lane 13 versus lane 14, in the revised manuscript). However, knockdown of *PERK* completely inhibited eIF2α phosphorylation (**Figure EV1D**, lanes 15, 16, 17, 18 versus lanes 13, 14, in the revised manuscript). These data indicated that PERK is the responsible kinase for eIF2α under ER stress. Taken together, we propose that FAM69C is a stress-specific kinase that phosphorylates eIF2α under oxidative stress and heat shock, but not related to ER stress.

- Fig EV1C: although the source data isn't available, there appears to be a mis-interpretation regarding the expression of il18. It seems like it hasn't met any fold change cutoff (although it did meet the Padj<0.05 cutoff). If it lies between log2FC+/-1, then it isn't differentially expressed in this experiment. The figure legend states that all log2FC>0 is considered, which supports my concern in the interpretation of the data. Highlighting the points for each gene of interest on the volcano plot would also be helpful. Also, the Pvalue stated in the text (p-value<0.05) does not match with the description in the figure legend (p-value < 0.1). Also, the actual measure of significance should mentioned in the text and the figure legend: the adjusted P value (Padj = q-value) instead of p-value (which is before the differential gene expression calling software made corrections).

[Response] We thank the reviewer for this question. According to the editor's suggestion, we have adjusted the logic flow in the revised manuscript. Specifically, we substantiate the role of FAM69C as eIF2α kinase in SG assembly and toned down its link to inflammasome formation as an accompanying consequence. Following this

logic flow, we observed that FAM69C-dependent SG assembly correlated with reduced inflammasome activation. We thus examined the NLRP3 pathway, including increased NLRP3 expression, cleavage of Caspase 1 and mRNA levels of pro-inflammatory cytokine *Il18*, as well as more ASC specks in the aged *Fam69c*$^{-/-}$ mice (**Figures 7A-7E** in the revised manuscript). These data are in consistent with the GSEA analyses of RNA sequencing data that indicated elevated immune response in the aged *Fam69c*$^{-/-}$ mice (**Figures for referees not shown**).

As for the RNA sequencing data in Figure EV1C in the original manuscript, we have deposited the source data in the GEO database with the accession code GSE214646 and included a table of differentially expressed gene in **Supplementary Table 2** in the revised manuscript. We labeled *Il18* in Response Figure 1 (**Figures for referees not shown**). We considered candidate genes with $log_2FC>0.2$. Moreover, we did qPCR and suggested that mRNA levels of *Il18* were significantly increased in the aged *Fam69c*$^{-/-}$ mice (**Figure 7C** in the revised manuscript).

- Fig EV3A: why aren't all the golgi markers present in every cell? Where these transfected? Are antibodies to these native proteins unavailable? They are not listed in the antibody section of the materials and methods section.

[Response] We thank the reviewer for this question. Limited by the lasers equipped in the confocal microscope, it is difficult to image FAM69C, cis Golgi, medial Golgi, trans Golgi and DAPI simultaneously in each cell. In Figure EV3A in the original manuscript, SH-SY5Y cells with stably transfected HA-tagged FAM69C were used to examine the subcellular localization of FAM69C. For Golgi localization analysis, we used GM130 antibody to label cis Golgi. Either Scarlet-Giantin-C1 plasmid for the labeling of medial Golgi or plasmid YFP-TGN38 for the labeling of trans Golgi were transiently transfected in SH-SY5Y cells with stable expression of HA-tagged FAM69C.

Given that NLRP3 is recruited to the dispersed trans-Golgi network (TGN) for aggregation and activation[2], we examined the localization of FAM69C in Golgi. Figure EV3A in the original manuscript indicated that FAM69C localized in the cis Golgi and medial Golgi but not in the trans-Golgi. Moreover, we directly examined the localization of FAM69C and NLRP3 and found these two molecules did not colocalize (**Figure EV3D** in the revised manuscript). To this end, we thought Figure EV3A in the original manuscript is redundant and thus removed in the revised manuscript.

- Typo: page 7. severer instead of severe

[Response] We have changed it to “severe” in the revised manuscript.

- Typo: it is stated: "whereas no difference was observed when treated with LPS and - nigericin (Fig. EV2B)." it looks like EV2B only assesses stress granule formation in SY5Y cells upon nigericin (+/-LPS) treatment, not ASC specks and therefore doesn't comment on the inflammasome. It was probably meant to direct to the bottom panel of figure 3A, although the underlying assertion should be quantified

[Response] We apologize for the misunderstanding and have corrected it in the revised manuscript.

- in the text: "In addition, we also detected a significantly increased phosphorylation level of eIF2α in BV-2 cells when treated with ATP, which may responsible for SG assembly. (Figure. 4C)." The word significantly should be removed unless a statistical analysis of blot densitometry was performed and added to the manuscript.
[Response] We thank the reviewer for this question. We have done the statistical quantification of this blot (**Figure EV5** in the revised manuscript).

- In the text: "western blotting revealed that protein translation was completely inhibited in response to AS treatment". Completely is much too strong, suggest change to "severely"
[Response] We have changed "completely" to "severely" in the revised manuscript.

- In the text: "Western blotting revealed that levels of phosphorylated eIF2α at S51 were significantly lower in FAM69C-/- SH5Y cells, as compared with FAM69C+/+ SH5Y cells (Figure. 6F)." Please strike "significantly" unless statistical analysis is being performed.
[Response] We agreed with the reviewer and have done the statistical analysis in **Figure 1E** in the revised manuscript.

- Materials and methods should indicate how transfections were performed
[Response] We have described the methods for transient transfection and stable transfection.

- The description of bulk RNAseq analysis is insufficient. What were the sequencing conditions and what mapper was used?
[Response] We have described the methods related to bulk RNA sequencing analysis, sequencing conditions and the mapper used in detail in the revised manuscript.

Referee #3:

FAM69C is a putative kinase protein for which the cellular and molecular functions are not well defined. In this manuscript, Wu et al., demonstrate the role of FAM69C in stress granule formation and inflammasome regulation. The authors first demonstrated the inflammasome activation signals in aged mice lacking FAM69C expression, which encouraged them to test its role in microglial inflammasome activation and stress granule (SG) formation. The authors conclude that FAM69C interacts with eIF2a to promote its phosphorylation and SG formation. Also, the authors have tried to provide some clues showing in FAM69C-dependent regulation of inflammasome activation in microglial cells. FAM69C role in phosphorylating eIF2a and SGs formation is interesting, and the experimental data in this manuscript

supports this concept and stands out as an important and only function of FAM69C. However, FAM69C role in inflammasome regulation is poor and the experimental data is not convincing. The authors tried to show that FAM69C mediated formation of SGs abolishes inflammasome activation (similar to the recent demonstration of DDX3X role in SGs and NLRP3 inflammasome regulation), however, there is no direct evidence for this claim. Please see my specific comments below.

We thank the reviewer for his/her critical and constructive comments, which helped us to improve our manuscript. The logic flow for this study has been adequately adjusted in the revised manuscript. Specifically, we have substantiated the role of FAM69C as eIF2α kinase in stress granule (SG) assembly and toned down its indirect regulation of inflammasome. The following efforts have been made to address the reviewer's concerns. First, we showed that FAM69C-dependent formation of SGs correlated with reduced inflammasome activation through sequestration of DDX3X in SG. Second, sing-cell RNA sequencing data of microglia from human brain provided evidence that microglia with high expression of ATP-sensing purinergic receptor P2RX4, P2RX7 and P2RY12 showing low expression levels of *Il1b*[3], indicating that ATP activation in microglia deviates from inflammasome activation. Indeed, our experiments demonstrated that ATP induces FAM69C-dependent SGs assembly in microglia, which correlated with reduced inflammasome activation. These data suggest that microglia and monocyte-derived macrophage show different features in response to ATP. Third, we have applied multiple inflammsome inducers and showed that ATP, but not other inducers, triggers FAM69C-dependent SGs formation. Based on these data, we agreed with the reviewer that FAM69C is directly involved in SG assembly through eIF2α, whereas restrain of inflammasome activation is paralleled with sequestration of DDX3X in SG. To this end, we have substantially revised the manuscript. A point-to-point response to the reviewer's comments is as following.

1. Does FAM69C colocalize/recruit in SGs and inflammasome complexes? Is FAM69C a kinase to trigger SGs assembly, like PKR, but not the integral component of SGs? Why do authors show FAM69C localization to Golgi without any premise? Unlike the DDX3X role shown in previous studies, the FAM69C has an indirect role in inflammasome activation and do not participate in inflammasome activation. FAM69C mediated SG formation might sequester DDX3X and inhibits its role in NLRP3 inflammasome activation.

[Response] We thank the reviewer for these questions. As shown in **Figure EV2A** in the revised manuscript, FAM69C was not an integral component of SGs. However, FAM69C is a stress-specific kinase for eIF2α that triggers SGs assembly. Given that NLRP3 is recruited to the dispersed trans-Golgi network (TGN) for aggregation and activation[2], we examined the localization of FAM69C in Golgi as indicated in Figures EV3A and 3B in the original manuscript. Immunofluorescence staining showed that FAM69C resided in the cis Golgi and medial Golgi, but not in the trans-Golgi. Further we examined the localization of FAM69C and NLRP3, and found that these two

molecules did not co-localize (**Figure EV3D** in the revised manuscript). Based on these data, we agreed with the reviewer that FAM69C does not directly participate in inflammasome activation. As the reviewer suggested, we tested whether FAM69C-mediated SG formation might sequester DDX3X. Interestingly, we observed that DDX3X was sequestered in SG that was marked by G3BP1 in *Fam69c*$^{+/+}$ microglia upon ATP/LPS+ATP treatment (**Figure 4D** in the revised manuscript). By contrast, FAM69C deficiency led to much less SG formation and DDX3X sequestration in SG. Taken together, we provided additional data on DDX3X, which support that FAM69C mediated SG formation and DDX3X sequestration in SG under ATP treatment in microglia. The sequestration of DDX3X into SG correlated with decreased inflammasome activation. However, we only observed a correlation between SG formation and reduced inflammasome activation, and agree with the reviewer that causal data is lacking in our study. We have discussed about this shortage in the revised manuscript.

2. Based on the experimental data, it appears that FAM69C is predominantly a regulator of the SGs but not inflammasome activation. Does FAM69C regulate the priming or activation step of NLRP3 inflammasome activation? If FAM69C is primarily a kinase working at the level of translation, it affects the expression of NLRP3 inflammasome components (priming step) instead of the activation and assembly of the NLRP3 inflammasome. There is no data showing i) NLRP3, IL-1b and CASP1 expression levels in FAM69C-knockout microglia ii) NLRP3 ubiquitination and oligomerization in the presence and absence of FAM69C cells.
[Response] We thank the reviewer for this question. First, we tested whether FAM69C might regulate the priming step of NLRP3 inflammasome activation. As shown in **Figure EV3B** in the revised manuscript, LPS treatment increased NLRP3 expression in microglia. However, there was no difference in the levels of NLRP3, CASP1 and ASC between *Fam69c*$^{+/+}$ and *Fam69c*$^{-/-}$ microglia. This data indicated that the priming step of the NLRP3 inflammasome is independent of FAM69C. Second, as previously reported, post-translational modification of NLRP3 includes phosphorylation and ubiquitination, which will either potentiates or inhibits NLRP3 oligomerization[4]. For example, PKA, PKD and JNK1 phosphorylate NLRP3, whereas PP2A dephosphorylates NLRP3, followed by changes in NLRP3 activation. To determine whether FAM69C might affect the post-translational modification of NLRP3 and the following NLRP3 activation, we first performed immunoprecipitation experiments and found that FAM69C did not physically interact with NLRP3. We thus premised that FAM69C might not affect the post-translational modification of NLRP3. Given that the quantity of primary culture of microglia was much lower than the BMDM culture for the oligomerization and ubiquitination experiments under LPS and LPS/nigericin treatment, we have tried but failed to complete this experiment. Thus, we suggested that FAM69C is not involved in the priming and activation step of NLRP3 inflammasome in the revised manuscript.

3. Figure-1: Authors considered the expression of IL-18 and its associated genes as a correlation to indicate inflammasome activation, which is incorrect. IL-18 expression is regulated by many inflammatory stimuli (PMID: 28468974).

[Response] We agreed with the reviewer's suggestion and have modified related interpretation of the data in the revised manuscript. It is well demonstrated that the assembly of inflammasomes induces IL-1β and IL-18 secretion[5]. In addition to the quantification of mRNA levels of *Il18*, we examined NLRP3 protein levels as well as cleavage of CASP1 by western blotting. We observed significantly increased levels of NLRP3 and elevated CASP1 cleavage in aged *Fam69c*$^{-/-}$ mice (**Figures 7A and 7B** in the revised manuscript). Moreover, immunofluorescence of cortical slices from *Fam69c*$^{-/-}$ mice revealed more ASC-positive microglia as compared with *Fam69c*$^{+/+}$ mice (**Figures 7D and 7E** in the revised manuscript). Taken together, these data suggested inflammasome activation in aged *Fam69c*$^{-/-}$ mice.

4. Figure-2: In a recent study by the same authors (Cell Reports, 2022), memory impairment was observed in 3-6 months old mice deficient in FAM69C expression. This suggests the role of FAM69C in the early stages of the mice causing neurodegeneration or neuronal abnormalities. However, in this study, all the inflammasome activation signals reported are in 10-12-month old mice. 3-month old mice did not show any CASP1 cleavage signals. These experiments suggest that inflammasome activation is a mere consequence of established neurodegeneration or loss of neuronal homeostasis in the brain of the 3-6-month old mice. FAM69C may not be the trigger for micrgolia-mediated inflammation and subsequent neurodegeneration as it was previously observed in Alzheimer's and Parkinson's mouse models. Fig 2A- provide FAM69C expression blots. Fig 2E- This imaging data need ASC specks quantification to support the authors' conclusions. In addition, SG staining (G3BP1) in these sections is critical to understanding FAM69C role in coordinating stress granules and inflammasome activation in the cortex.

[Response] According to previous studies, aggregated amyloid-β and α-synuclein act as a danger-associated molecular pattern (DAMP) and activate inflammasome, which further promotes propagation of toxic protein aggregates and results in neuronal death[6]. In our mouse model, we observed inflammsome activation in 12-month-old *Fam69c*$^{-/-}$ mice, but not in 3-month-old *Fam69c*$^{-/-}$ mice, which may be a consequence of disturbed neuronal homeostasis initiated from the ages of 3-6 months old. We agreed with the reviewer that FAM69C is not the trigger for microglia-mediated inflammation. According to both the reviewer's and editor's suggestions, we have substantially toned down the role of FAM69C in the regulation of inflammation in the revised manuscript.

In **Figure 7A** in the revised manuscript, we have shown FAM69C expression blots as well as NLRP3 expression blots. In addition, we have done the quantification for ASC-expression microglia in the mPFC from 12-month-old *Fam69c*$^{+/+}$ and *Fam69c*$^{-/-}$ mice (**Figures 7D and 7E** in the revised manuscript).

We have tried to stain G3BP1 in the brain sections of 12-month-old *Fam69c*$^{+/+}$ mice and *Fam69c*$^{-/-}$ mice. However, we did not see SGs *in vivo*, which may be limited

by technique issues (**Response Figure 1**, see below). Given that assembly of SGs is a dynamic process that strictly depends on acute stress stimulation, it is rarely seen *in vivo* without exogenous stimuli. Consistently, a previous study indicated that SGs could only be seen after *in vivo* arsenite treatment[7]. Considering that it is difficult to capture the dynamic process of SG assembly *in vivo*, we did the primary microglia culture from *Fam69c*$^{+/+}$ and *Fam69c*$^{-/-}$ mice. Upon a series of treatments, we provided solid evidence that FAM69C mediates ATP-induced SGs assembly in microglia and sequestration of DDX3X in SGs, which correlated with reduced inflammsome activation (**Figure 4** in the revised manuscript). Further, we demonstrated that induction of stress granule in *Fam69c*$^{-/-}$ microglia rescued exacerbated inflammasome activation (**Figure 6** in the revised manuscript). **Figure 5** in the revised manuscript also illustrates FAM69C-independent inflammasome activation. We thus concluded that FAM69C-mediated stress granule assembly, rather FAM69C, precludes inflammasome formation. Taken together, we agreed with the reviewer that FAM69C is not the direct trigger for microglia-mediated inflammation and has accordingly adjusted the interpretation of data in the revised manuscript.

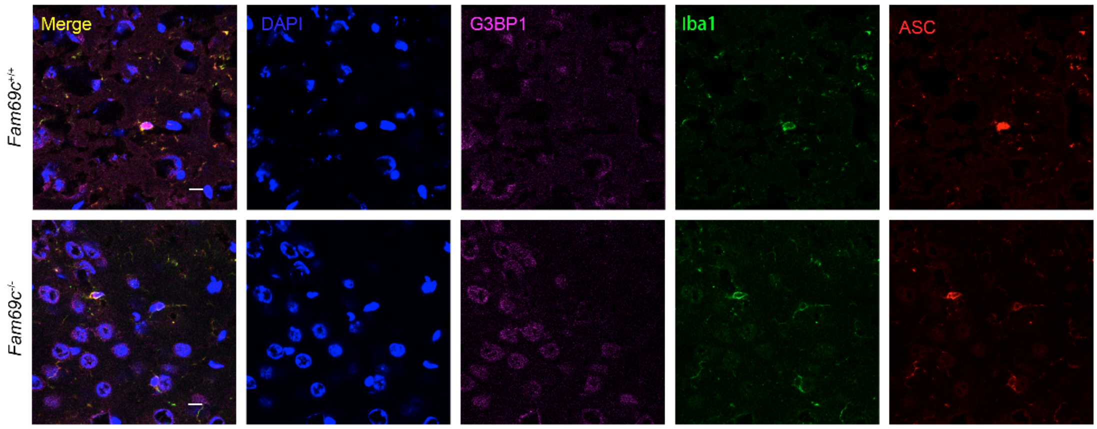

Response Figure 1. Staining of stress granule (G3BP1) and inflammasome (ASC) in 12-month-old *Fam69c*$^{+/+}$ and *Fam69c*$^{-/-}$ cortical tissues. Iba1 indicates microglia.

5. Figure-3: The experimental data and the conclusions in this figure are confusing and inconclusive. This data indicates that lack of FAM69C does not affect NLRP3 inflammasome activation but abolishes SGs formation. This suggests FAM69C role in SG activation but not in inflammasome regulation. It is known that LPS or LPS+ATP stimulation do not induce SG formation in BMDMs despite expressing the P2X4 receptor but induces robust inflammasome activation. The authors observed no inflammasome activation/ASC specks in WT-microglia (Fam69C+/+) but the presence of SGs after LPS+ATP treatment. Based on these observations, the authors concluded that ATP is a bona fide trigger for SGs in microglia, which is not convincing. Multiple triggers of NLRP3 inflammasome (imiquimod, silica or polyI:C+Nig) need to be tested to substantiate this claim. Also, in Fig-3C, FAM69C knock-out cells still show SG formation (in fact, more than WT cells) after arsenite and ATP treatment, contradicting the authors own conclusions.

[Response] We apologize for the confusing data presentation. We have reorganized the data and added new data in the revised manuscript. The following efforts were made to address the reviewer's concerns. First, we treated microglia or BMDM with LPS, LPS+ATP in parallel and observed that microglia form SGs (**Figures 4A and 4B** in the revised manuscript), whereas BMDM forms inflammasome (**Response Figure 2**, see below). In addition, we treated microglia or BMDM with LPS, LPs+nigericin and found that both microglia (**Figures 5A and 5B** in the revised manuscript) and BMDM forms inflammasome, but not SGs (**Response Figure 2**, see below). These data indicated that microglia, but not BMDM forms SGs in response to ATP. Second, we treated microglia with ATP, nigericin and Leu-Leu-OMe. We only observed SG assembly upon ATP treatment, and the other classical NLRP3 inducers did not induce SGs (**Figures 4A-4C, 5A-5F** in the revised manuscript). Third, we showed that only ATP but not nigericin increased the phosphorylation of eIF2α, which is essential for the induction of SG assembly (**Figure EV5** in the revised manuscript). Forth, single-nucleus sequencing data from human microglia reported that microglia expressing P2RX4, P2RX7 and P2RY12, the purinergic receptors that sense ATP, showed reduced expression of *Il1B* and *NLRP3* (**Response Figure 3**, see below)[3]. These data indicate that ATP activation in microglia deviates from inflammasome activation. Fifth, western blotting revealed microglia but not BMDM expresses FAM69C, which may partially explain different aspects of microglia and BMDM in SG assembly under ATP (**Response Figure 4**, see below). Based on these experiments, we demonstrated that ATP elicits SG assembly in FAM69C-expressing microglia, but not in FAM69C-null BMDM.

Moreover, we applied multiple triggers of inflammasome, including nigericin and Leu-Leu-OMe[8]. We found that only ATP triggers SG in microglia (**Figures 4A-4C** in the revised manuscript), whereas nigericin and Leu-Leu-OMe activate inflammasome in microglia (**Figures 5A-5F** in the revised manuscript).

To support the finding that SG assembly could precldue ASC speck formation, we applied arsenite and sorbitol as SG inducers. As shown in **Figures 5B-5C** in the revised manuscript, LPS/nigericin treatment induced NLRP3 inflammasome activation in the microglia. Remarkably, pretreatment with arsenite triggered SG assembly, which abolished ASC specks (**Figures 6A and 6B** in the revised manuscript). Consistently, we applied another SG inducer Sorbitol and found that SG assembly prevented NLRP3 inflammasome activation (**Figures 6C and 6D** in the revised manuscript).

In Figures 5A and 5B in the original manuscript, we demonstrated that loss of FAM69C resulted in a remarkable delay of stress granule assembly in response to arsenite treatment. Based on this observation, we hypothesized that a prolonged treatment of arsenite for 40 min may finalize stress granule assembly even in the $FAM69C^{-/-}$ cells. Thus, in Figure 3C of the original manuscript, $FAM69C^{+/+}$ and $FAM69C^{-/-}$ were primed with 1 μg/ml LPS for 3h and then treated with 0.5 mM arsenite for 20 min, and followed with 5 mM ATP for 40 min in a mixed culture of astrocyte and microglia. After this combined treatment, we observed SG assembly in $FAM69C^{-/-}$ cells, with absence of ASC specks. We thus proposed that induction of SG

assembly precludes inflammasome activation. To strengthen this data, we treated *FAM69C*$^{+/+}$ and *FAM69C*$^{-/-}$ with 1 μg/ml LPS for 3h and then with 0.5 mM AS for 40 min, and followed with 5 mM ATP for 40 min in primary microglia. Consistently, we observed that prolonged treatment with AS finalized SG assembly in *FAM69C*$^{-/-}$cells and inhibited ASC specks (**Figures 6E and 6F** in the revised manuscript).

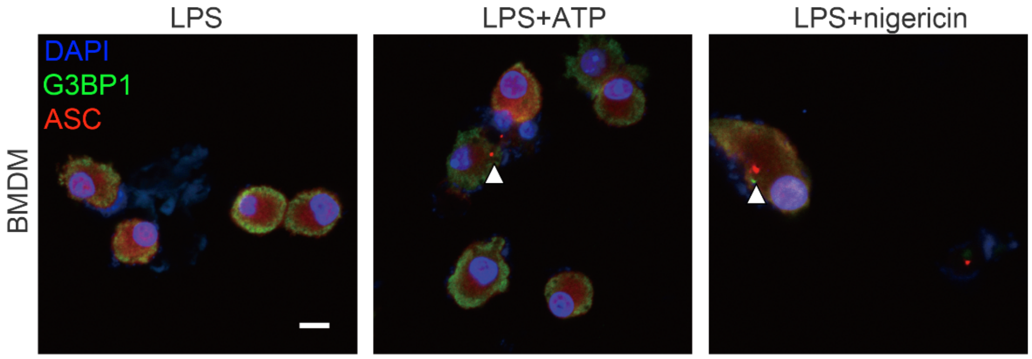

Response Figure 2. Immunofluorescence for stress granule (G3BP1) and inflammasome (ASC) in BMDM upon LPS, LPS/ATP or LPS/nigericin treatment.

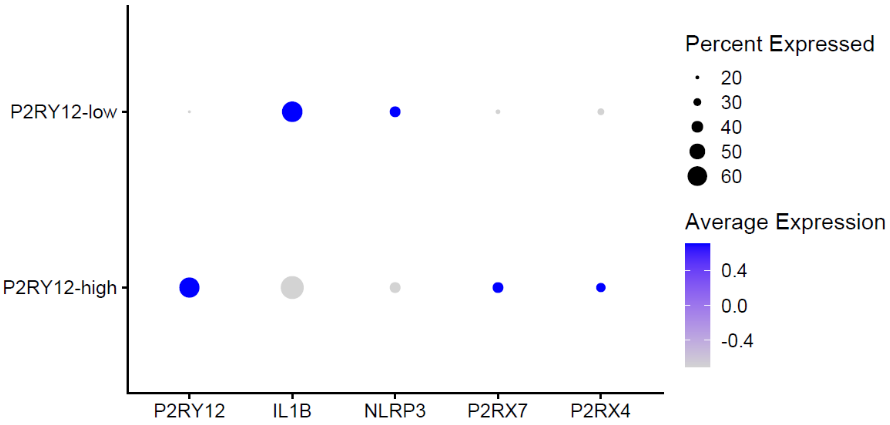

Response Figure 3. Expression of indicated genes from single-nucleus sequencing data from human microglia.

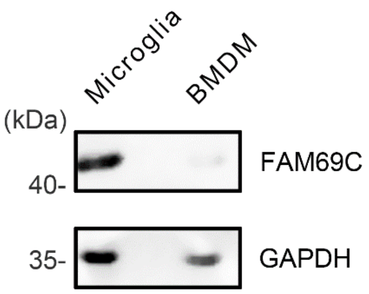

Response Figure 4. Western blotting for FAM69C in microglia and BMDM from wild-type mice.

6. Figure-4: Although authors claim that ATP induces SGs in microglial cells, SGs in BV-2 cells after ATP treatment is barely minimal and not appealing. Fig 4E- It is surprising to see the ASC speck count in cells treated with LPS and arsenite. Arsenite treatment abolishes inflammasome activation and ASC speck formation. Fig 4D- To substantiate the role of FAM69C in SG formation, authors need to use multiple triggers of SGs to prove this concept.

[Response] We thank the reviewer for these questions. We repeated the experiment in Figure 4B in the original manuscript and agreed with the reviewer that SGs in BV-2 after ATP treatment is not as appealing as that in the primary microglia culture (**Figures 3A-3D, 4A-4D** in the revised manuscript). We thus applied all ATP and LPS/ATP treatment on primary microglia and removed the redundant data on BV-2 cells in the revised manuscript.

We apologize for the misleading presentation of the data in Figures 4D and 4E in the original manuscript. There were no ASC specks in the primary glia culture after LPS and arsenite treatment, as clearly seen in Figure 4D in the original manuscript. Figure 4E is the quantification of Figure 4D. Given that this experiment is done in a mixture of astrocyte and glia culture, we stained ASC and used it as a marker for microglia. ASC-negative stands for astrocyte, whereas ASC-positive represents microglia. ASC is not meaning ASC specks in this context. We compared the ratio of cells that forms SG in astrocyte and microglia separately. As this data presentation causes misunderstanding, we did primary microglia culture, and applied the ATP treatment in primary microglia in the revised manuscript. We observed a greater number of SGs assembly in *Fam69c*$^{+/+}$ microglia, which is correlated with reduced ASC specks. On the other hand, there were much less SGs in *Fam69c*$^{-/-}$ microglia, correlating with more ASC specks (**Figures 4A-4C** in the revised manuscript).

To substantiate the role of FAM69C in SG formation in Figure 4D in the original manuscript, we applied both arsenite and ATP (**Figures 3A-3D** in the revised manuscript). Consistently, these data showed that lack of FAM69C leads to defected SG assembly.

7. Figure-5: Do primary microglial cells lacking FAM69C expression show delay in SGs assembly or defects in SGs formation?? This is not clear in the manuscript. Also, in Fig 5C, the authors claim that FAM69C knock-out cells show partial differences in protein translation despite a significant difference similar to WT- cells. eIF2a phosphorylation blot is critical to understanding the blots in Fig 5C. Overall, FAM69C role in SG activation in neuronal cells appears to be critical and can be linked to its function in memory loss instead of its role in inflammasome activation.

[Response] We thank the reviewer for these comments. As shown in **Figures 2C and 2D** in the revised manuscript**,** we provided evidence that loss of FAM69C in SH-SY5Y cells with stably expression of G3BP1-GFP resulted in delayed SGs assembly by live cell imaging. In primary microglia, although live cell imaging for G3BP1-GFP is not applicable, we found that prolonged treatment of AS from 20 min to 40 min successfully finalized SGs assembly in *Fam69c*$^{-/-}$ microglia (**Figures 6E and 6F** in

the revised manuscript). Based on these data, we suggest that lacking FAM69C expression in microglia showed delay in SGs assembly.

In addition, we have added the blot for eIF2α phosphorylation, which show consistency with the puromycin incorporation data (**Figure 2A and 2B** in the revised manuscript). These data suggested that FAM69C deficiency leads to aberrant arrest of protein translation through eIF2α under arsenite treatment. We agreed with the reviewer that we have provided solid data showing taht FAM69C directly regulates SG assembly. We have adequately adjusted the logic flow of this study and comprehensively revised the manuscript. Specifically, we substantiate the role of FAM69C in regulation of SG and tone down its indirect role in inflammasome activation in the revised manuscript.

8. "With the development of sequencing technology, the microglial-specific gene TREM2 R47H mutation identified with GWASs completely changed our understanding of the relationship between immunity and neurodegeneration". This sentence is exactly the same as the sentence in the introduction. Also, "SG and inflammasome assembly determine contrasting live-or-die fates of stressed macrophages" is repeated.

[Response] We apologize for these errors and have corrected them in the revised manuscript.

9. Figure EV3: The figure panel citations and the representations are improper in the manuscript text.

[Response] Figure EV3 in the original manuscript is redundant and has been removed in the revised manuscript.

10. Figure legends: Figures legends miss essential experimental information: mice age, time of arsenite treatment, time of collection of images.

[Response] We thank the reviewer for these suggestions and included essential experimental information in the revised figure legends.

Reference

1 Samir, P. *et al.* DDX3X acts as a live-or-die checkpoint in stressed cells by regulating NLRP3 inflammasome. *Nature* **573**, 590-594, doi:10.1038/s41586-019-1551-2 (2019).

2 Chen, J. & Chen, Z. J. PtdIns4P on dispersed trans-Golgi network mediates NLRP3 inflammasome activation. *Nature* **564**, 71-76, doi:10.1038/s41586-018-0761-3 (2018).

3 Kumar, P. *et al.* Single-cell transcriptomics and surface epitope detection in human brain epileptic lesions identifies pro-inflammatory signaling. *Nat Neurosci* **25**, 956-966, doi:10.1038/s41593-022-01095-5 (2022).

4 Mangan, M. S. J. *et al.* Targeting the NLRP3 inflammasome in inflammatory diseases. *Nat Rev Drug Discov* **17**, 588-606, doi:10.1038/nrd.2018.97 (2018).

5 Gong, T., Liu, L., Jiang, W. & Zhou, R. DAMP-sensing receptors in sterile inflammation

and inflammatory diseases. *Nat Rev Immunol* **20**, 95-112, doi:10.1038/s41577-019-0215-7 (2020).

6 Heneka, M. T., McManus, R. M. & Latz, E. Inflammasome signalling in brain function and neurodegenerative disease. *Nat Rev Neurosci* **19**, 610-621, doi:10.1038/s41583-018-0055-7 (2018).

7 Zhang, X. *et al.* In vivo stress granule misprocessing evidenced in a FUS knock-in ALS mouse model. *Brain* **143**, 1350-1367, doi:10.1093/brain/awaa076 (2020).

8 Hornung, V. *et al.* Silica crystals and aluminum salts activate the NALP3 inflammasome through phagosomal destabilization. *Nat Immunol* **9**, 847-856, doi:10.1038/ni.1631 (2008).

2nd Revision - Editorial Decision **3rd Feb 2023**

Dear Prof. Yin

Thank you for the submission of your revised manuscript to EMBO reports. We have now received the full set of referee reports that is copied below.

As you will see, all three referees are positive about the revised version and support publication after a minor revision to clarify figures, text and conclusions on the contribution of HRI. In addition to these minor concerns, referee 2 notes that the RNAseq data do not meet the standards needed for publication, showing high variability between the replicates and inadequate statistical analysis. The referee suggests to either repeat the experiment or to remove that data from the manuscript. I overall feel that the data are not essential to support the conclusions and could therefore indeed be removed. You might want to consider replacing it with qRT-PCR experiments for a handful of candidates or omit it all together. I am also happy to discuss this point further via e-mail or video chat, if you wish.

From the editorial side, there are also a few things that we need before we can proceed with the official acceptance of your study.

- Please update the 'Conflict of interest' paragraph to our new 'Disclosure and competing interests statement'. For more information see
https://www.embopress.org/page/journal/14693178/authorguide#conflictsofinterest

- Regarding the Author Contributions, we now use CRediT to specify the contributions of each author in the journal submission system. CRediT replaces the author contribution section, which therefore needs to be removed from the manuscript text. You can use the free text box in our system if you wish to provide more detailed descriptions. See also guide to authors https://www.embopress.org/page/journal/14693178/authorguide#authorshipguidelines.

- References: If there are more than 10 authors, please only list the first 10 followed by et al.

- We noticed that you specified different grant numbers in the manuscript file (2021YFA1300601) and in our online submission system (2016YFA0500302) for the same funder National Key Research and Development Program of China. Please double-check and correct this information if necessary.

- In Figure 6C, the zoomed image does not match the highlight box. Please check.

- Supplementary table 1 should be renamed to Dataset EV1 with its legend in a separate tab of the .xls sheet. Please also correct the callouts in the text.
- The same applies to Supplementary table 2, in case it will remain part of the manuscript.

- I attach to this email a related manuscript file with comments by our data editors. Please address all comments and upload a revised file with tracked changes with your final manuscript submission.

- Finally, EMBO reports papers are accompanied online by A) a short (1-2 sentences) summary of the findings and their significance, B) 2-3 bullet points highlighting key results and C) a synopsis image that is 550x300-600 pixels large (width x height) in PNG for JPG format. You can either show a model or key data in the synopsis image. Please note that the size is rather small and that text needs to be readable at the final size. Please send us this information along with the revised manuscript.

We look forward to seeing a final version of your manuscript as soon as possible.

Kind regards,

Martina Rembold, PhD
Senior Editor
EMBO reports

****

Referee #1:

Wu and colleagues have reorganized the manuscript by focusing on the role of FAM69C as an eIF2alpha kinase promoting stress granule assembly. They responded in detail to all questions and suggestions from each reviewer. With this additional work and reframing of the manuscript, my remaining concerns were addressed. The overall quality of the manuscript has been

substantially improved. This work will be of great interest to the field.

Referee #2:

This resubmission addresses the role of the FAM69C kinase in regulating the stress response. The authors show that FAM69C is an eIF2 kinase and that contributes to stress granule formation and examine the anti-correlation between stress granule and inflammasome formation. The authors have addressed most of the concerns raised in the previous round of review, however there remain major concerns regarding the RNAseq data. Specific concerns follow:
- Fig 1B: the labels on the side of the blots are confusing. Suggest putting the IP/Input labels on separate side of blot than the eIF2alpha/FAM69C labels. This would allow the reader to intuit that FAM69C was pulled down and then the purified sample was probed for either eIF2alpha/FAM69C (as stated in figure legend).
- Fig EV1C: This is a nice experiment. Regarding lanes 7 to 9 & 10, it looks like they have phospho-eIF2alpha at about the same levels, suggesting that HRI is dispensable for eIF2alpha phosphorylation in this system which should be noted in the text. Contrary to this interpretation, on page 7 the authors state that "Further reduction in HRI abolished eIf2α phosphorylation" and "These data indicated that both FAM69C and HRI are the physiologically-relevant kinases that phosphorylate eIF2α under AS-induced oxidative stress." Quantitating the blot by densitometry would help clarify the relative contribution of HRI vs FAM69C to eIF2alpha phosphorylation.
- Fig 7G-H: The RNAseq data is not of publication quality due to variability in the replicates. This is best illustrated when looking at the data in Table S2. For their interpretation, the authors use Pvalue as a statistical measure, but the corrected Pvalue (Padj=FDR) is the essential value to use for this measure given the assumptions behind how the differential expression algorithms work. If you were to limit analysis to FDR<0.05, only 3 genes (of over 30,000) reach statistical significance. Also, the authors state in their response that they selected a cutoff of log2FC= +/- 0.2 (which is a ~15% change in gene expression - the standard is a twofold change: log2FC = +/-1). Selecting genes that vary by 15% and do not meet a moderately robust statistical cutoff is going to compromise downstream analyses like GSEA, rendering them likely to report variations due to noise. Given the issues the reliability of the RNAseq data, it would be best to repeat the experiment or remove it from the manuscript.
- Typo: "complete sequestration of DDX3X, whereas Fam69c-/+-microglia showed no signs of" genotype incorrect.
- Sentence fragment: "To this end, ATP exposure, but not other inflammasome inducers related to the crosstalk between FAM69C-dependnet SG assembly and inflammasome activation in microglia."

Referee #3:

In this revised manuscript, Wu et al., have considered comments raised by this reviewer seriously and have done experiments thoroughly to substantiate the conclusion of this manuscript. The newly revised data shows that FAM69C preferably involves in promoting stress granules via eIF2a and has an indirect role in inflammasome activation in macrophages/microglial cells. The authors' revised conclusions suggesting FAM69C is not the trigger for microglia-mediated inflammation are important, and they have attempted to justify the conclusions with the data. I do not have any further comments.

Referee #2:

This resubmission addresses the role of the FAM69C kinase in regulating the stress response. The authors show that FAM69C is an eIF2 kinase and that contributes to stress granule formation and examine the anti-correlation between stress granule and inflammasome formation. The authors have addressed most of the concerns raised in the previous round of review, however there remain major concerns regarding the RNAseq data. Specific concerns follow:
We thank the referee for his/her critical and constructive comments, which help us to significantly improve the quality of our manuscript.

- Fig 1B: the labels on the side of the blots are confusing. Suggest putting the IP/Input labels on separate side of blot than the eIF2alpha/FAM69C labels. This would allow the reader to intuit that FAM69C was pulled down and then the purified sample was probed for either eIF2alpha/FAM69C (as stated in figure legend).
[Response] We have corrected the IP/Input labels in the revised manuscript.

- Fig EV1C: This is a nice experiment. Regarding lanes 7 to 9 & 10, it looks like they have phospho-eIF2alpha at about the same levels, suggesting that HRI is dispensable for eIF2alpha phosphorylation in this system which should be noted in the text. Contrary to this interpretation, on page 7 the authors state that "Further reduction in HRI abolished eIf2α phosphorylation" and "These data indicated that both FAM69C and HRI are the physiologically-relevant kinases that phosphorylate eIF2α under AS-induced oxidative stress." Quantitating the blot by densitometry would help clarify the relative contribution of HRI vs FAM69C to eIF2alpha phosphorylation.
[Response] We apologize for the missing statistical quantitation in Fig EV1C and the confusion. As shown in Figure EV1D in the revised manuscript, knockdown of HRI significantly reduced the phosphorylation of eIF2α in *FAM69C*$^{+/+}$ cells. Accordingly, we suggest that both FAM69C and HRI are indispensable for the phosphorylation of eIF2α under AS-induced oxidative stress.

- Fig 7G-H: The RNAseq data is not of publication quality due to variability in the replicates. This is best illustrated when looking at the data in Table S2. For their interpretation, the authors use Pvalue as a statistical measure, but the corrected Pvalue (Padj=FDR) is the essential value to use for this measure given the assumptions behind how the differential expression algorithms work. If you were to limit analysis to FDR<0.05, only 3 genes (of over 30,000) reach statistical significance. Also, the authors state in their response that they selected a cutoff of log2FC= +/- 0.2 (which is a ~15% change in gene expression - the standard is a twofold change: log2FC = +/-1). Selecting genes that vary by 15% and do not meet a moderately robust statistical cutoff is going to compromise downstream analyses like GSEA, rendering them likely to report variations due to noise. Given the issues the reliability of the RNAseq data, it would be best to repeat the experiment or remove it from the manuscript.

[Response] We thank the referee for this comment. We agree that the high variability between the replicates and inadequate statistical analysis in RNAseq data made it not meet the standards for publication. Considering that the data are not essential to support our conclusion, we removed the RNAseq data from the manuscript.

- Typo: "complete sequestration of DDX3X, whereas Fam69c-/+-microglia showed no signs of" genotype incorrect.
[Response] We have corrected it to "*Fam69c*$^{-/-}$ microglia" in the revised manuscript.

- Sentence fragment: "To this end, ATP exposure, but not other inflammasome inducers related to the crosstalk between FAM69C-dependnet SG assembly and inflammasome activation in microglia."
[Response] We have corrected the sentence fragment in the revised manuscript.

Prof. Yuxin Yin
Peking University Health Science Center
Department of Pathology
38 Xue Yuan Road
Beijing 100191
China

Dear Prof. Yin,

I am very pleased to accept your manuscript for publication in the next available issue of EMBO reports. Thank you for your contribution to our journal.

At the end of this email I include important information about how to proceed. Please ensure that you take the time to read the information and complete and return the necessary forms to allow us to publish your manuscript as quickly as possible.

As part of the EMBO publication's Transparent Editorial Process, EMBO reports publishes online a Review Process File to accompany accepted manuscripts. As you are aware, this File will be published in conjunction with your paper and will include the referee reports, your point-by-point response and all pertinent correspondence relating to the manuscript.

If you do NOT want this File to be published, please inform the editorial office within 2 days, if you have not done so already, otherwise the File will be published by default [contact: emboreports@embo.org]. If you do opt out, the Review Process File link will point to the following statement: "No Review Process File is available with this article, as the authors have chosen not to make the review process public in this case." Please note that the author checklist will still be published even if you opt out of the transparent process.

Thank you again for your contribution to EMBO reports and congratulations on a successful publication. Please consider us again in the future for your most exciting work.

Yours sincerely,

Martina Rembold, PhD
Senior Editor
EMBO reports

****

THINGS TO DO NOW:

Please note that you will be contacted by Wiley Author Services to complete licensing and payment information. The required 'Page Charges Authorization Form' is available here: https://www.embopress.org/pb-assets/embo-site/er_apc.pdf - please download and complete the form and return to embopressproduction@wiley.com

EMBO Press participates in many Publish and Read agreements that allow authors to publish Open Access with reduced/no publication charges. Check your eligibility: https://authorservices.wiley.com/author-resources/Journal-Authors/open-access/affiliation-policies-payments/index.html

You will receive proofs by e-mail approximately 2-3 weeks after all relevant files have been sent to our Production Office; you should return your corrections within 2 days of receiving the proofs.

Please inform us if there is likely to be any difficulty in reaching you at the above address at that time. Failure to meet our deadlines may result in a delay of publication, or publication without your corrections.

All further communications concerning your paper should quote reference number EMBOR-2022-55641V3 and be addressed to emboreports@wiley.com.

Should you be planning a Press Release on your article, please get in contact with emboreports@wiley.com as early as possible, in order to coordinate publication and release dates.

EMBO Press Author Checklist

Corresponding Author Name: Yuxin Yin	
Journal Submitted to: EMBO reports	
Manuscript Number: EMBOR-2022-55641V2	

USEFUL LINKS FOR COMPLETING THIS FORM
The EMBO Journal - Author Guidelines
EMBO Reports - Author Guidelines
Molecular Systems Biology - Author Guidelines
EMBO Molecular Medicine - Author Guidelines

Reporting Checklist for Life Science Articles (updated January 2022)
This checklist is adapted from Materials Design Analysis Reporting (MDAR) Checklist for Authors. MDAR establishes a minimum set of requirements in transparent reporting in the life sciences (see Statement of Task: 10.31222/osf.io/9sm4x). Please follow the journal's guidelines in preparing your manuscript.
Please note that a copy of this checklist will be published alongside your article.

Abridged guidelines for figures
1. Data
The data shown in figures should satisfy the following conditions:
- the data were obtained and processed according to the field’s best practice and are presented to reflect the results of the experiments in an accurate and unbiased manner.
- ideally, figure panels should include only measurements that are directly comparable to each other and obtained with the same assay.
- plots include clearly labeled error bars for independent experiments and sample sizes. Unless justified, error bars should not be shown for technical replicates.
- if n<5, the individual data points from each experiment should be plotted. Any statistical test employed should be justified.
- Source Data should be included to report the data underlying figures according to the guidelines set out in the authorship guidelines on Data Presentation.

2. Captions
Each figure caption should contain the following information, for each panel where they are relevant:
- a specification of the experimental system investigated (eg cell line, species name).
- the assay(s) and method(s) used to carry out the reported observations and measurements.
- an explicit mention of the biological and chemical entity(ies) that are being measured.
- an explicit mention of the biological and chemical entity(ies) that are altered/varied/perturbed in a controlled manner.
- the exact sample size (n) for each experimental group/condition, given as a number, not a range;
- a description of the sample collection allowing the reader to understand whether the samples represent technical or biological replicates (including how many animals, litters, cultures, etc.).
- a statement of how many times the experiment shown was independently replicated in the laboratory.
- definitions of statistical methods and measures:
  - common tests, such as t-test (please specify whether paired vs. unpaired), simple χ2 tests, Wilcoxon and Mann-Whitney tests, can be unambiguously identified by name only, but more complex techniques should be described in the methods section;
  - are tests one-sided or two-sided?
  - are there adjustments for multiple comparisons?
  - exact statistical test results, e.g., P values = x but not P values < x;
  - definition of ‘center values’ as median or average;
  - definition of error bars as s.d. or s.e.m.

Please complete ALL of the questions below.
Select "Not Applicable" only when the requested information is not relevant for your study.

Materials

Newly Created Materials	Information included in the manuscript?	In which section is the information available? (Reagents and Tools Table, Materials and Methods, Figures, Data Availability Section)
New materials and reagents need to be available; do any restrictions apply?	Not Applicable	

Antibodies	Information included in the manuscript?	In which section is the information available? (Reagents and Tools Table, Materials and Methods, Figures, Data Availability Section)
For **antibodies** provide the following information:<br>- Commercial antibodies: RRID (if possible) or supplier name, catalogue number and or/clone number<br>- Non-commercial: RRID or citation	Yes	Materials and Methods

DNA and RNA sequences	Information included in the manuscript?	In which section is the information available? (Reagents and Tools Table, Materials and Methods, Figures, Data Availability Section)
**Short novel DNA or RNA including primers, probes**: provide the sequences.	Yes	Materials and Methods

Cell materials	Information included in the manuscript?	In which section is the information available? (Reagents and Tools Table, Materials and Methods, Figures, Data Availability Section)
**Cell lines:** Provide species information, strain. Provide accession number in repository **OR** supplier name, catalog number, clone number, and/**OR** RRID.	Yes	Materials and Methods
**Primary cultures:** Provide species, strain, sex of origin, genetic modification status.	Yes	Materials and Methods
Report if the cell lines were recently **authenticated** (e.g., by STR profiling) and tested for mycoplasma contamination.	Yes	Materials and Methods

Experimental animals	Information included in the manuscript?	In which section is the information available? (Reagents and Tools Table, Materials and Methods, Figures, Data Availability Section)
**Laboratory animals or Model organisms:** Provide species, strain, sex, age, genetic modification status. Provide accession number in repository **OR** supplier name, catalog number, clone number, **OR** RRID.	Yes	Materials and Methods
**Animal observed in or captured from the field:** Provide species, sex, and age where possible.	Yes	Materials and Methods
Please detail **housing and husbandry conditions**.	Yes	Materials and Methods

Plants and microbes	Information included in the manuscript?	In which section is the information available? (Reagents and Tools Table, Materials and Methods, Figures, Data Availability Section)
**Plants:** provide species and strain, ecotype and cultivar where relevant, unique accession number if available, and source (including location for collected wild specimens).	Not Applicable	
**Microbes:** provide species and strain, unique accession number if available, and source.	Not Applicable	

Human research participants	Information included in the manuscript?	In which section is the information available? (Reagents and Tools Table, Materials and Methods, Figures, Data Availability Section)
If collected and within the bounds of privacy constraints report on age, sex and gender or ethnicity for all study participants.	Not Applicable	

Core facilities	Information included in the manuscript?	In which section is the information available? (Reagents and Tools Table, Materials and Methods, Figures, Data Availability Section)
If your work benefited from core facilities, was their service mentioned in the acknowledgments section?	Not Applicable	

Design

Study protocol	Information included in the manuscript?	In which section is the information available? (Reagents and Tools Table, Materials and Methods, Figures, Data Availability Section)
If study protocol has been **pre-registered, provide DOI in the manuscript**. For clinical trials, provide the trial registration number **OR** cite DOI.	Not Applicable	
Report the **clinical trial registration number** (at ClinicalTrials.gov or equivalent), where applicable.	Not Applicable	

Laboratory protocol	Information included in the manuscript?	In which section is the information available? (Reagents and Tools Table, Materials and Methods, Figures, Data Availability Section)
Provide DOI OR other citation details if **external detailed step-by-step protocols** are available.	Not Applicable	

Experimental study design and statistics	Information included in the manuscript?	In which section is the information available? (Reagents and Tools Table, Materials and Methods, Figures, Data Availability Section)
Include a statement about **sample size** estimate even if no statistical methods were used.	Not Applicable	
Were any steps taken to minimize the effects of subjective bias when allocating animals/samples to treatment (e.g. **randomization procedure**)? If yes, have they been described?	Not Applicable	
Include a statement about **blinding** even if no blinding was done.	Not Applicable	
Describe **inclusion/exclusion criteria** if samples or animals were excluded from the analysis. Were the criteria pre-established?<br><br>If sample or data points were omitted from analysis, report if this was due to attrition or intentional exclusion and provide justification.	Not Applicable	
For every figure, are **statistical tests** justified as appropriate? Do the data meet the assumptions of the tests (e.g., normal distribution)? Describe any methods used to assess it. Is there an estimate of variation within each group of data? Is the variance similar between the groups that are being statistically compared?	Yes	Figures

Sample definition and in-laboratory replication	Information included in the manuscript?	In which section is the information available? (Reagents and Tools Table, Materials and Methods, Figures, Data Availability Section)
In the figure legends: state number of times the experiment was **replicated** in laboratory.	Yes	Figures
In the figure legends: define whether data describe **technical or biological replicates**.	Yes	Figures

Ethics

Ethics	Information included in the manuscript?	In which section is the information available? (Reagents and Tools Table, Materials and Methods, Figures, Data Availability Section)
Studies involving **human participants**: State details of **authority granting ethics approval** (IRB or equivalent committee(s), provide reference number for approval.	Not Applicable	
Studies involving **human participants**: Include a statement confirming that **informed consent** was obtained from all subjects and that the experiments conformed to the principles set out in the WMA Declaration of Helsinki and the Department of Health and Human Services Belmont Report.	Not Applicable	
Studies involving **human participants:** For publication of **patient photos**, include a statement confirming that consent to publish was obtained.	Not Applicable	
Studies involving experimental **animals**: State details of **authority granting ethics approval** (IRB or equivalent committee(s), provide reference number for approval. Include a statement of compliance with ethical regulations.	Yes	Materials and Methods
Studies involving **specimen and field samples:** State if relevant **permits** obtained, provide details of authority approving study; if none were required, explain why.	Not Applicable	

Dual Use Research of Concern (DURC)	Information included in the manuscript?	In which section is the information available? (Reagents and Tools Table, Materials and Methods, Figures, Data Availability Section)
Could your study fall under dual use research restrictions? Please check biosecurity documents and list of **select agents and toxins** (CDC): https://www.selectagents.gov/sat/list.htm	Not Applicable	
If you used a select agent, is the security level of the lab appropriate and reported in the manuscript?	Not Applicable	
If a study is subject to dual use research of concern regulations, is the name of the **authority granting approval and reference number** for the regulatory approval provided in the manuscript?	Not Applicable	

Reporting

The MDAR framework recommends adoption of discipline-specific guidelines, established and endorsed through community initiatives. Journals have their own policy about requiring specific guidelines and recommendations to complement MDAR.

Adherence to community standards	Information included in the manuscript?	In which section is the information available? (Reagents and Tools Table, Materials and Methods, Figures, Data Availability Section)
State if relevant guidelines or checklists (e.g., **ICMJE, MIBBI, ARRIVE, PRISMA**) have been followed or provided.	Not Applicable	
For **tumor marker prognostic studies**, we recommend that you follow the **REMARK** reporting guidelines (see link list at top right). See author guidelines, under 'Reporting Guidelines'. Please confirm you have followed these guidelines.	Not Applicable	
For **phase II and III randomized controlled trials**, please refer to the **CONSORT** flow diagram (see link list at top right) and submit the CONSORT checklist (see link list at top right) with your submission. See author guidelines, under 'Reporting Guidelines'. Please confirm you have submitted this list.	Not Applicable	

Data Availability

Data availability	Information included in the manuscript?	In which section is the information available? (Reagents and Tools Table, Materials and Methods, Figures, Data Availability Section)
Have **primary datasets** been deposited according to the journal's guidelines (see 'Data Deposition' section) and the respective accession numbers provided in the Data Availability Section?	Yes	Data Availability
Were **human clinical and genomic datasets** deposited in a public access-controlled repository in accordance to ethical obligations to the patients and to the applicable consent agreement?	Not Applicable	
Are **computational models** that are central and integral to a study available without restrictions in a machine-readable form? Were the relevant accession numbers or links provided?	Not Applicable	
If publicly available data were reused, provide the respective **data citations in the reference list**.	Not Applicable	
